# Bacterial Contribution to Hydrogen Peroxide Decomposition in Lake Erie Cyanobacterial Harmful Algal Bloom Communities Is Dependent on Bloom Stage

**DOI:** 10.1111/1758-2229.70405

**Published:** 2026-09-22

**Authors:** Chelsea E. Donovan, Gabriel Padilla, Madison Ashbaugh, Sophie Bencivenga, Jordan Cibellis, Wilson Ritterpusch, Cameron Tierney, Justin D. Chaffin, Louie L. Wurch, Morgan M. Steffen

**Affiliations:** ^1^ Department of Biology James Madison University Harrisonburg Virginia USA; ^2^ F.T. Stone Laboratory and Ohio Sea Grant The Ohio State University Put‐in‐Bay Ohio USA

## Abstract

Cyanobacterial harmful algal blooms (cHABs) are expanding globally and often involve toxin‐producing species such as *Microcystis aeruginosa* that pose ecological and public health risks. Some cyanobacteria, including some strains of *M. aeruginosa* , lack antioxidant enzymes such as catalase, making them more reliant on associated bacteria for reactive oxygen species (ROS) decomposition. To evaluate bacterial contribution to oxidative stress defence in *Microcystis* blooms across stages of bloom development, we conducted a time‐series incubation after a pulse of elevated hydrogen peroxide spanning the 2023 Lake Erie bloom season during pre‐bloom, peak bloom, and bloom decline. Metatranscriptomics was used to assess bacterial gene expression for catalase, superoxide dismutase, and alkyl hydroperoxide reductase across 23 bacterial genera. Bacterial transcription of these enzymes was highest during peak bloom, with catalase eliciting the most robust transcriptional response. Bacterial taxa exhibited unique transcription patterns that fell into four categories: first responders, delayed responders, downregulators, or non‐responders, with the largest and most diversified response during peak bloom. These results support the hypothesis that *Microcystis* partially outsources oxidative stress defence to bacterial partners and provide new evidence that bacterial contribution depends on bloom stage.

## Graphical abstract

A seasonal time series metatranscriptome dataset suggests that neutralising hydrogen peroxide in *Microcystis* blooms requires a complex division of labour that is further complicated by seasonality and bloom development.

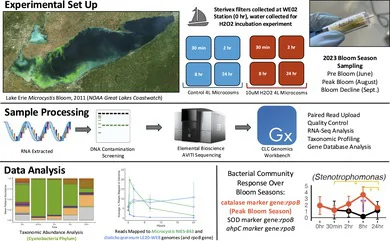

## 1 Introduction

Global freshwater resources are under increasing pressure due to rising temperatures, eutrophication, regional droughts, and the occurrence of toxic cyanobacterial harmful algal blooms (cHABs). Annual bloom events caused by organisms such as *Microcystis aeruginosa* have only increased in surface area and duration in globally important lakes such as Lake Erie (North America), Taihu (China), and Lake Victoria (Africa) over the last two decades (Roegner et al. 2020; Song, Zhang, et al. 2024; Watson et al. 2016). The ecological success of *M. aeruginosa* is driven by a complex suite of abiotic and biotic factors. Environmental variables such as nutrient availability, light intensity, temperature fluctuations, and water column stratification play a critical role in promoting bloom formation, persistence, and toxicity (Chaffin et al. 2018; Harke et al. 2016; Martin et al. 2020; Paerl et al. 2014; Paerl and Huisman 2009). In addition to these abiotic drivers, biotic interactions, particularly with microbes inhabiting the *Microcystis* phycosphere, are also now understood to contribute to bloom dynamics (Baylous et al. 2024; Große et al. 2025; Hoke et al. 2021; Zhao et al. 2023).

The colonial physiology of *Microcystis* spp. creates a carbon‐rich environment in which bacteria are tightly embedded and have been observed for decades (Dziallas and Grossart 2011, 2012; Kim et al. 2019; Komárek and Komárková 2002; Paerl 1982). This microenvironment surrounding algal cells (including cyanobacteria), termed the phycosphere, serves as a critical interface for supporting chemical currency exchange and interaction between algae and heterotrophic bacteria (Bell and Mitchell 1972; Seymour et al. 2017). Such interactions within cHAB systems such as *Microcystis* blooms play a pivotal role in supporting algal survival by facilitating nutrient cycling (Dickey et al. 2025; Seymour et al. 2017; Song, Li, et al. 2024; Zhao et al. 2023) and offering protection from environmental stressors like exogenous hydrogen peroxide exposure (Park et al. 2024; Smith et al. 2022). Hydrogen peroxide is a ubiquitous toxic stressor in aquatic environments, and it is well‐documented that certain marine autotrophs, such as the cyanobacterium *Prochlorococcus*, rely on heterotrophic bacteria to decompose hydrogen peroxide, as they lack the necessary enzymatic machinery (Morris et al. 2011, 2012). Like *Prochlorococcus*, *Microcystis* spp. often lack the capability to produce catalases but possess other reactive oxygen species (ROS) defence machinery, including peroxiredoxins and thioredoxins (Baylous et al. 2024; Kaneko et al. 2007; Kim et al. 2024). The role of bacteria in hydrogen peroxide decomposition may be more nuanced for *Microcystis* than for *Prochlorococcus*, however. At ambient concentrations, microcystin appears to bind to enzymes and provides protection from hydrogen peroxide stress, although this protective effect is lost at higher concentrations of hydrogen peroxide (Schuurmans et al. 2018; Zilliges et al. 2011). Therefore, *Microcystis* spp. may be forced to rely in part on the bacterial microbiome as well as non‐toxigenic cells within the bloom community for hydrogen peroxide decomposition under elevated conditions (Park et al. 2024; Smith et al. 2022). Cellular modelling indicates that there may be a balance between toxigenic and non‐toxigenic strains and hydrogen peroxide decomposition that both complicates and explains bloom community division of labour in *Microcystis*‐dominated systems (Hellweger et al. 2022). Our work extends this perspective by considering the contribution of catalase activity from associated heterotrophic bacteria as an additional ROS‐mitigation pathway. The impact of bacterial catalase activity on *Microcystis* varies among strains, yet does not appear to be associated with strain toxicity (Smith et al. 2022). In freshwater systems dominated by *Microcystis*, hydrogen peroxide levels > 9 μM have been reported, although concentrations vary depending on site and/or conditions and are generally in the nanomolar range (Marsico et al. 2015; Ndungu et al. 2019; Smith et al. 2022).

Bacterial contributions to *Microcystis* bloom microbiomes are increasingly understood to be seasonally regulated and closely linked to the growth dynamics, strain, and colony morphology of the *Microcystis* host (Hoke et al. 2021; Qin et al. 2023; Wu et al. 2019). Environmental fluctuations in temperature, nutrient availability, and light throughout the bloom season influence not only *Microcystis* dominance and toxicity but also the structure and metabolic activity of associated bacterial consortia (Hellweger et al. 2022; Lezcano et al. 2018; Li et al. 2011). During peak bloom conditions, these communities often become more functionally specialised, performing roles such as oxidative stress mitigation and nutrient recycling that support *Microcystis* persistence under chemically and physiologically challenging conditions (Le et al. 2023; Li et al. 2011; Mankiewicz‐Boczek and Font‐Nájera 2022). In contrast, pre‐ and post‐bloom bacterial communities tend to be more diverse and functionally flexible, possibly reflecting reduced host dependency and a greater responsiveness to external environmental pressures (Kim et al. 2020). Together, these seasonal dynamics suggest that microbial support for bloom development and resilience is temporally structured, with cooperative interactions peaking during periods of maximum *Microcystis* activity and stress. However, we do not yet fully understand how bacterial contribution to essential bloom functions like hydrogen peroxide decomposition is impacted by bloom stage and seasonality. To address this, we aimed to characterise the transcriptional responses of individual bacterial genera to an exogenous pulse of elevated hydrogen peroxide during three stages of bloom development during the 2023 bloom season in Lake Erie: early bloom (June), peak bloom (August) and bloom decline (late September). We exposed the Lake Erie cHAB community to a single pulse of elevated hydrogen peroxide, incubated samples in in situ conditions, and generated metatranscriptomes over a time series to assess bacterial expression of enzyme marker genes involved in oxidative stress response: catalase, superoxide dismutase (SOD), and alkyl hydroperoxide reductase (Smith et al. 2022). The results of this study targeting bacterial contribution to *Microcystis* bloom antioxidant defence suggest the function and interaction dynamics of members of the bloom holobiont are dependent on bloom stage and seasonality.

## 2 Experimental Procedures

### 2.1 Lake Erie Incubation Experiments

In situ hydrogen peroxide microcosm experiments were independently conducted three times during the 2023 Lake Erie bloom season. Surface water was collected from the NOAA GLERL station WE02 (41.76403, −83.32917) in the western basin of Lake Erie in 8 20‐L acid‐washed, sterile carboys on 28 June 2023 (pre‐bloom—no cyanobacteria detected via satellite in western Lake Erie) (NOAA 2023a), 8 August 2023 (peak bloom—mixed cyanobacterial bloom of 220 square miles) (NOAA 2023b), and 19 September 2023 (bloom decline—mixed cyanobacterial bloom of 120 square miles) (NOAA 2023c). Water column profiles (surface to lake bottom) of water temperature, pH, specific conductivity, turbidity (FNU), dissolved oxygen, and phycocyanin and chlorophyll (Chl) fluorescence were recorded with a calibrated Yellow Springs Instruments EXO2 sonde. The Secchi disk depth was measured. Water samples collected and returned to the laboratory were analysed for concentrations of total P and N (Patton and Kryskalla 2003), dissolved concentrations of nitrate, nitrite, ammonium, dissolved reactive P, and silicate on a SEAL Analytical QuAAtro39 using standard methods (Chaffin et al. 2019). The phytoplankton community was determined with a bbe++ Moldanke FluoroProbe (Beutler et al. 2002) with a cuvette bench top adaptor to measure the Chl *a* concentrations associated with four algal groups: (1) chlorophytes, (2) cyanobacteria, (3) diatoms and (4) cryptophytes and other algae. Total microcystins were quantified using the ELISA method, following the Ohio EPA protocol (Ohio EPA 2021) with Gold Standard Diagnostics (previously Eurofins Abraxis) ELISA kits (#520011‐OH) on the Abraxis automated CAAS Cube.

Water was transported to the Ohio State University Stone Lab in Put‐in‐Bay, OH, for in situ incubation experiments. Homogenised water was aliquoted into 24 4‐L acid rinsed Cubitainers (United States Plastic Corp, #76932). A triplicate set of 0 h samples was collected from homogenised water used to construct the microcosms prior to addition of the hydrogen peroxide pulse in microcosms. Half of the microcosms were unamended controls and half received a treatment of a nominal target of 10 μM molecular grade hydrogen peroxide at the onset of the time‐series. The 10 μM concentration reflects high‐end environmentally observed hydrogen peroxide concentrations in natural waters (> 9 μM) and aligns with prior preliminary experiments demonstrating efficacy of 10 μM against *Microcystis* populations collected from a small retention pond in Harrisonburg, VA, USA (Marsico et al. 2015; Ndungu et al. 2019; Smith et al. 2022; Puma 2023). This is representative of a single pulse of hydrogen peroxide and subsequent oxidative stress on the cHAB communities. All samples were incubated at in situ lake conditions in floating corrals located at the dock at the Ohio State University Stone Laboratory. Incubations were started at approximately 5:30 PM local time (EST) and microcosms were destructively sampled in triplicate for biomass for RNA‐Seq, chlorophyll *a* (Chl *a*) and fluorescence measurements across a timeseries of 30 min, 2 h, 8 h, and 24 h. For RNA samples, 180 mL of water was filtered through 0.22 μm Sterivex filters (EMD Millipore Corporation) to collect biomass. Filters were flash frozen in liquid nitrogen for transport from the field station and then stored at −80°C until extraction. Chl *a* concentration was obtained using an AquaFlash Handheld Fluorometer (Turner Designs Inc.). Pigments were evaluated using unpaired *t*‐tests (via the ‘stats’ package in R) for comparison between treatments at each timepoint (hydrogen peroxide vs. control) with a significance cutoff of *p* < 0.05. Results were visualised in GraphPad Prism (v10.5.0).

### 2.2 RNA Extraction and Sequencing

Total RNA was extracted from Sterivex filters using the RNeasy PowerWater kit (QIAGEN, Germantown, MD, USA) following manufacturer guidelines and modified to remove the outer casing prior to extraction (Cruaud et al. 2017). Extracted total RNA was stored at −80°C prior to DNase treatment and sequencing. Samples were screened for DNA contamination using 16S universal PCR amplification and visualised with gel electrophoresis (Baylous et al. 2024; Steffen et al. 2015). Samples containing residual DNA were treated with Invitrogen TURBO Dnase kit using the rigorous decontamination protocol to remove all contaminating DNA (Thermo Fisher Scientific, Waltham, MA, USA).

### 2.3 Gene Expression Analysis and Statistics

RNA quantification and integrity were assessed using an Agilent TapeStation. rRNA was depleted using the Ribo‐off rRNA Depletion Kit V2, and libraries were prepared with the VAHTS Universal V10 RNA‐seq Library Prep Kit for Illumina. Library quality was verified with Qubit, qPCR, and TapeStation analysis. Paired‐end sequencing (2 × 150 bp) was performed on the Element Biosciences AVITI platform by AmpSeq (Gaithersburg, MD) (Arslan et al. 2024).

Raw sequence files are available via the National Center for Biotechnology Information Sequence Read Archive in BioProject PRJNA1282059. Sequence libraries were imported and paired using the standard import for Element Bioscience reads in CLC Genomics Workbench (v.24.0.1) with low quality reads discarded upon import. Following import into CLC Genomics Workbench, quality control screening was performed using the QC for Sequencing Reads function within the Prepare Sequencing Data module to assess read quality prior to downstream analysis. Expression analysis for *M. aeruginosa* NIES 843 (NC_010296) and *Dolichospermum* strain LE20‐WE8 (GCA_046449505.1) was performed using RNASeq Analysis under the RNASeq & Small RNA Module in CLC Genomics Workbench. Default settings were used with the exception of length fraction and similarity fraction, which were both increased to 0.9 to increase stringency. Expression values were calculated as transcripts per million (TPM) and unmapped reads were collected for downstream analyses. Reads mapped to the *Microcystis aeruginosa* NIES‐843 and *Dolichospermum* LE20‐WE8 reference genomes were used to calculate genome‐level relative abundance, expressed as the proportion of mapped reads relative to total RNA‐Seq reads per sample. Reads mapped to the *rpoB* gene within each reference genome were similarly used to calculate gene‐level relative abundance. Statistical comparisons were performed using unpaired *t*‐tests (via the ‘stats’ package in R) with a significance cutoff of *p* < 0.05 following outlier screening with the Tukey interquartile range (IQR) method; no data points met the criteria for exclusion. For both genome‐ and gene‐level datasets, three comparison types were conducted: (1) between treatments at each timepoint (hydrogen peroxide vs. control), (2) across timepoints within hydrogen peroxide treated samples, and (3) across timepoints within control samples. Results were visualised in R (v4.3.1) using the ‘ggplot2’ and ‘patchwork’ packages for time series plotting and multi‐panel figure assembly.

The Taxonomic Profiling tool in the Microbial Genomics Module was used to classify and quantify relative abundance of taxonomic groups within each sample. Taxonomic profiles were generated using the full short read libraries. Changes in relative abundance over the time series were evaluated at both the phylum and genus levels using unpaired *t*‐tests (via the ‘stats’ package in R), with a minimum *p* value of < 0.05 for significance. Three comparison types were conducted: (1) between treatments at each timepoint (hydrogen peroxide vs. control), (2) across timepoints within hydrogen peroxide treated samples, and (3) across timepoints within control samples. Outlier detection was performed using the Tukey interquartile range (IQR) method; no outliers were identified, and all samples were retained for analysis. Taxonomic profiling data were visualised in R (v4.3.1) using the ‘ggplot2’ and ‘patchwork’ packages for time series plotting and multi‐panel figure assembly.

To test the functional contribution of bacteria to ROS neutralisation, we targeted specific marker genes associated with antioxidant responses: catalase‐peroxidase/catalase (including *katG*, *katA*, *katE*, *ydbD*), superoxide dismutase (including *sodA*, *sodB*, *sodC*, *sodF*, *sodN*, *sodX*), and alkyl hydroperoxide reductase (including *ahpC‐F*) (Table S2) (Smith et al. 2022). Reads that did not map to the *M. aeruginosa* NIES 843 genome during RNA‐Seq analysis were aligned to a custom database of marker gene sequences that was constructed based on the taxonomic profile of bacteria present in the samples generated in this study and supplemented with sequences from representative strains of genera previously shown to play a role in ROS neutralisation or other functional role during *Microcystis* blooms (Table S1). An *rpoB* database was constructed in parallel for normalisation of functional gene expression (Patole et al. 2021; Smith et al. 2022). Unmapped reads were aligned to the functional gene and housekeeping gene databases in CLC Genomics Workbench using the Map Reads to Reference function under the *de novo* sequencing module. All default settings were maintained with the exception of length fraction and similarity fraction which were set to 0.5 and 0.7, respectively (Krausfeldt et al. 2019). Slightly lowering these values allows for partial reads to be mapped, which can be useful for environmental data where the reference genome may not be a perfect match (Rey‐Campos et al. 2022). Differential expression of oxidative stress response enzymes was assessed by calculating gene‐to‐*rpoB* expression ratios across the experimental time series (0 h, 30 min, 2 h, 8 h, 24 h) (Smith et al. 2022). A standard Tukey interquartile range (IQR) test was used to screen for potential outliers; however, no data points met the criteria for exclusion, and all samples were retained. To evaluate transcriptional responses, unpaired *t*‐tests (using the ‘stats’ and ‘rstatix’ packages in R) were conducted across three comparison types: (1) between treatments at each timepoint (hydrogen peroxide vs. control), (2) within the hydrogen peroxide time series, and (3) within the control time series with a minimum *p* value of < 0.05 to indicate significance. Statistically significant differences that occurred in both hydrogen peroxide treated and control samples were excluded from interpretation to minimise false differential expression not due to exogenous hydrogen peroxide, as these could have been driven by external factors such as diel variability or bottle effects rather than a true treatment effect to the pulse of elevated hydrogen peroxide at the onset of the incubation period. Differential expression data were visualised in R (v4.3.1) using the ‘ggplot2’ and ‘patchwork’ packages for time series plotting and panel figure assembly.

## 3 Results

### 3.1 Environmental Conditions

Water for incubation experiments was collected from NOAA GLERL station WE02 in the western basin of Lake Erie on three dates in 2023 representing different stages of bloom development: June (pre‐bloom), August (peak bloom), and September (bloom decline). Environmental conditions at station WE02 are summarised in Table 1. Notably, water temperature, Chl *a*, microcystin concentration, and total phosphorus concentration (TP) were all elevated during peak bloom compared to the other sampling periods (Table 1).

**TABLE 1:** Summary of environmental conditions at Lake Erie station WE02 during sampling.

Bloom stage	Date/time	Depth (m)	Secchi depth (m)	1 m water temp (°C)	Chl *a* (μg/L)	Microcystin (μg/L)	TP (μmol/L)	TN (μmol/L)
Pre‐bloom	28 June 2023 11:11 AM	5.3	1.42	22.02	15.24	0.30	0.93	65.52
Peak bloom	09 August 2023 11:00 AM	5.5	0.78	24.08	58.52	2.24	1.56	61.32
Bloom decline	19 September 2023 11:45 AM	5.2	1.46	21.05	12.36	0.190	0.66	29.47

### 3.2 Pigment Response to Hydrogen Peroxide

**FIGURE 1:**
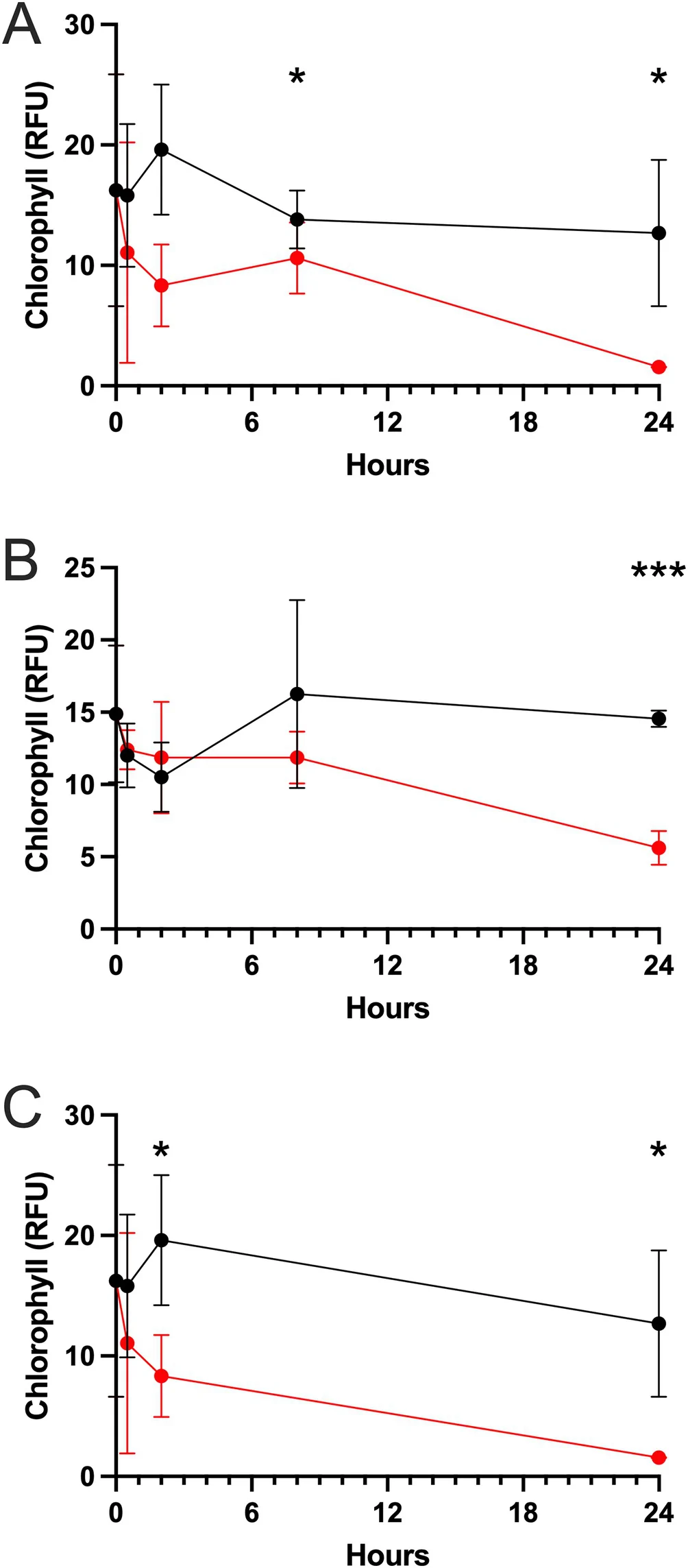
Pigment data (Chl *a*) concentration from pre‐bloom (A—June 2023), peak bloom (B—August 2023), and bloom decline (C—September 2023). Significance between control (black) and hydrogen peroxide (red) is indicated as *p < 0.05, ***p < 0.0005.

Chl *a* was measured as a proxy for *Microcystis* and other cyanobacteria growth response to the addition of hydrogen peroxide. For pre‐bloom samples (June), the treatment group did not immediately diverge from the control following exposure to hydrogen peroxide at 30 min or 2 h (Figure 1). However, at 8 h, Chl *a* was significantly lower in hydrogen peroxide samples than the control (*p* = 0.034; Figure 1 and Table S3). Chl *a* remained significantly lower in pre‐bloom samples at 24 h (*p* = 0.039; Figure 1 and Table S3). The peak bloom (August) hydrogen peroxide samples responded differently than pre‐bloom, as Chl *a* did not significantly decline by 30 min, 2 h, or 8 h after hydrogen peroxide exposure (Figure 1). Chl *a* was significantly lower in peak bloom samples at 24 h after exposure to hydrogen peroxide (*p* = 0.0003; Figure 1 and Table S3). Bloom decline samples (September) followed a similar pattern to the pre‐bloom samples, as 2 h (*p* = 0.034; Table S3) and 24 h (*p* = 0.038; Table S3) Chl *a* values were significantly lower in the treatment group (Figure 1).

### 3.3 Impact of Hydrogen Peroxide on cHAB‐Forming CyanobacteriacHAB‐Forming Cyanobacteria

**FIGURE 2:**
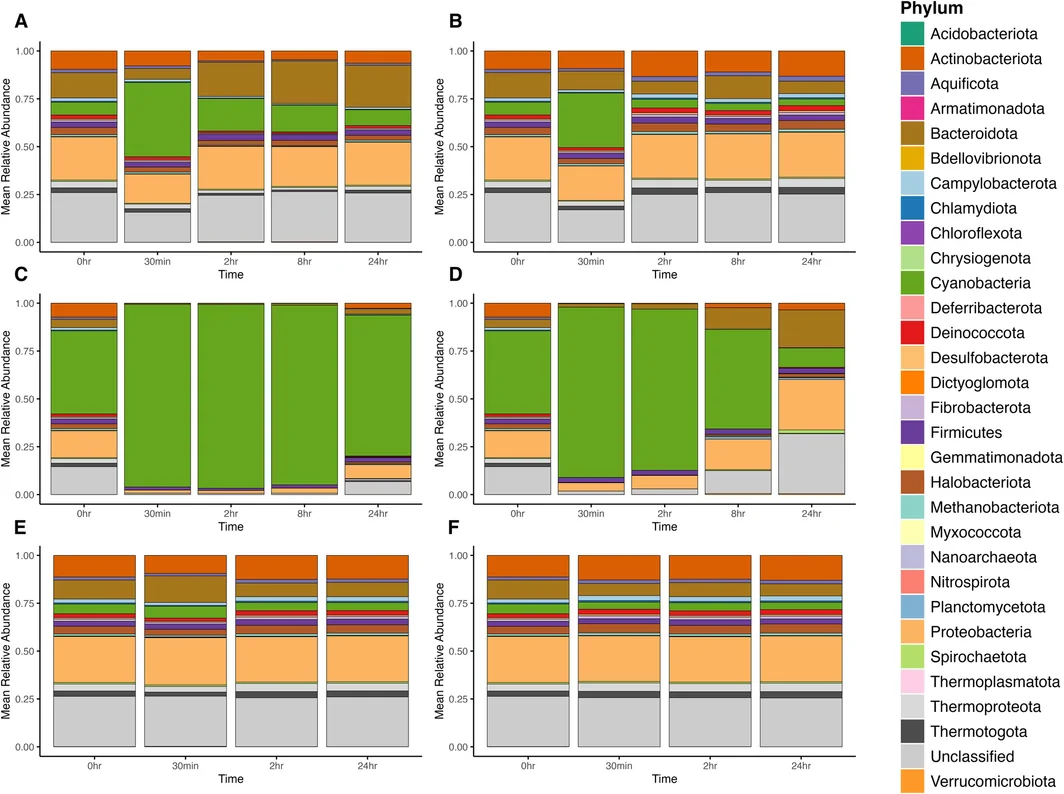
Phylum‐level composition of bacterial communities in control (A, C, E) and hydrogen peroxide treated (10 μM) samples (B, D, F) from pre‐bloom (A, B, June 2023), peak bloom (C, D, August 2023), and bloom decline (E, F, September 2023). Bars represent the mean relative abundance of each phylum across biological replicates at five timepoints (0 h, 30 min, 2 h, 8 h, and 24 h). The 8 h timepoint was not sampled during the September bloom decline (E, F).

**FIGURE 3:**
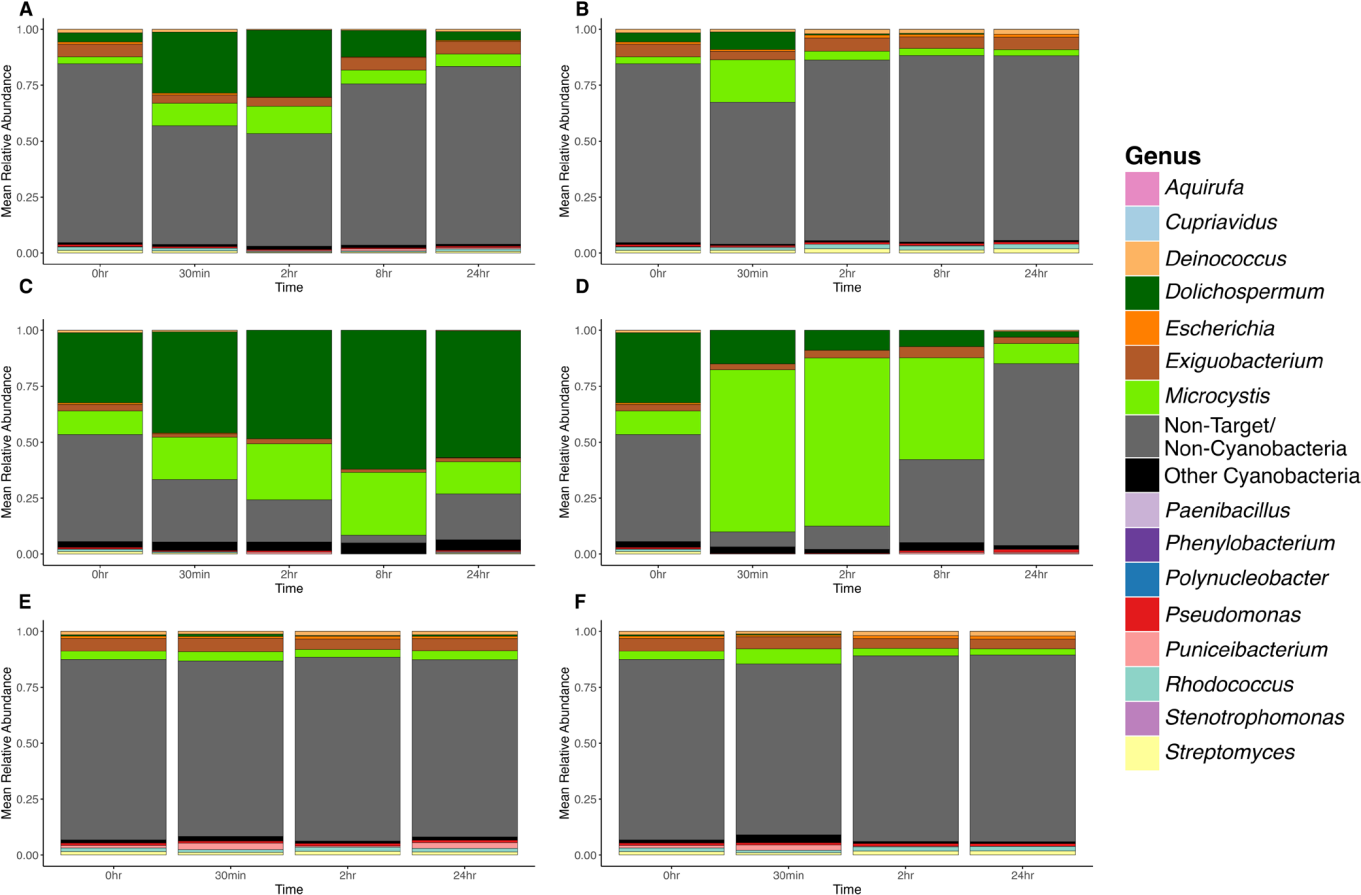
Genus‐level composition of bacterial communities in control (A, C, E) and hydrogen peroxide treated (10 μM) samples (B, D, F) from pre‐bloom (A, B, June 2023), peak bloom (C, D, August 2023), and bloom decline (E, F, September 2023). Bars represent the mean relative abundance of each phylum across biological replicates at five timepoints (0 h, 30 min, 2 h, 8 h, and 24 h). The 8 h timepoint was not sampled during the September bloom decline (E, F).

Taxonomic abundance analysis was conducted to evaluate changes in the relative contribution of microbial community members to the transcript pool under control and treatment conditions. Community composition was assessed at the phylum (Figure 2), order (Figure S1), and genus (Figure 3) levels. In both the pre‐bloom and bloom decline incubations, the relative abundance of transcripts produced by members of the Cyanobacteria phylum was not significantly impacted by exogenous hydrogen peroxide exposure (Figure 2). During the pre‐bloom season, Cyanobacteria relative abundance was low (5.8%) relative to peak bloom abundance (39.5%; Figure 2 and Table S8). After hydrogen peroxide treatment, relative abundance rose at 30 min (25.6%) but did not differ from earlier time points or the control, then declined and stayed below 4% for the remainder of the experiment (Figure 2 and Table S8). During bloom decline, Cyanobacteria relative abundance remained consistently low, ranging from 3%–8% across all time points (Figure 2 and Table S8). The only significant change observed between the treatment samples was a statistically significant decline in relative abundance between the 2 h (3.6%) and 24 h (3.2%) timepoints (*p* = 0.0072).

In contrast, the peak bloom incubation demonstrated a pronounced shift in Cyanobacteria transcript relative abundance compared to the 0 h. At the onset of the experiment, relative abundance of treated samples was 39.5%, increasing sharply to 88.2% within 30 min of hydrogen peroxide exposure (Table S8). Cyanobacteria transcript relative abundance remained above 80% at 2 h (82.8%; Table S8) but was significantly lower than the 30 min timepoint (*p* = 0.0479; Table S8). A further significant reduction in Cyanobacteria was observed between the 2 h and 24 h timepoints (82.8% > 8.5%; *p* = 0.0258; Table S8). By 8 h, relative abundance had already dropped to 49.9%, with final abundance at 24 h declining to below the 0 h Cyanobacteria transcript relative abundance (Figure 2 and Table S8). To account for potential bottle effects and/or transcriptional changes related to the day: night cycle, control samples were also evaluated over time. Cyanobacteria transcript relative abundance increased from 39.5% at 0 h to > 90% at 30 min, 2 h, and 8 h before decreasing to 71.3% at 24 h, with no significant differences among control timepoints (Table S8). Furthermore, at 2 h there was significantly higher relative abundance of Cyanobacteria in the control (96.0%; Table S8) compared to the hydrogen peroxide treatment (82.8%; *p* = 0.008; Table S8).

Taxonomic abundance analysis during the August peak bloom revealed co‐dominance by *Microcystis* and another cyanobacterial genus, *Dolichospermum* (Figure 3C–D; Table S10). During this period, *Microcystis* exhibited dynamic changes in transcript abundance that followed trends observed at the phylum level (Figures 2 and 3; Table S9). In hydrogen peroxide‐treated samples, the relative abundance of *Microcystis* rose sharply from 4.1% at the start of the incubation to 19.8% at 30 min, remaining elevated at 2 h (15.12%). While differences between treated timepoints were not statistically significant, *Microcystis* abundance was significantly higher in treated samples compared to controls at both 30 min (19.8% vs. 7.1%, *p* = 0.0235) and 2 h (15.1% vs. 8.28%, *p* = 0.000175; Table S9 and Figure 3C,D). After 2 h, treated sample abundance declined to 5.1% at 8 h and 1.2% at 24 h. In contrast, control samples remained relatively stable, fluctuating between 4.0% and 10.0% throughout the time series without any significant differences across timepoints (Table S9 and Figure 3A,B,E,F). No significant changes in *Microcystis* abundance were detected during the pre‐bloom or bloom decline periods, indicating oxidative stress was specific to peak bloom conditions.

*Dolichospermum* transcript abundance responded differently to hydrogen peroxide exposure during the same peak bloom period. In control samples, relative abundance increased modestly from 5.8% at 0 h to 7.9% at 30 min and 9.5% at 2 h, with no significant differences when compared to hydrogen peroxide treated samples at those timepoints (5.7%, 1.9%, and 0.9%, respectively; Table S10 and Figure 3C,D). However, by 8 h, control samples exhibited significantly higher *Dolichospermum* abundance than treated samples (8.1% vs. 0.3%, *p* = 0.016), a disparity that continued through 24 h (10.7% vs. 0.2%). Similar patterns of lower *Dolichospermum* abundance in treated samples were observed during the pre‐bloom and bloom decline seasons, though none of these differences were statistically significant (Figure 3 and Table S10).

**FIGURE 4:**
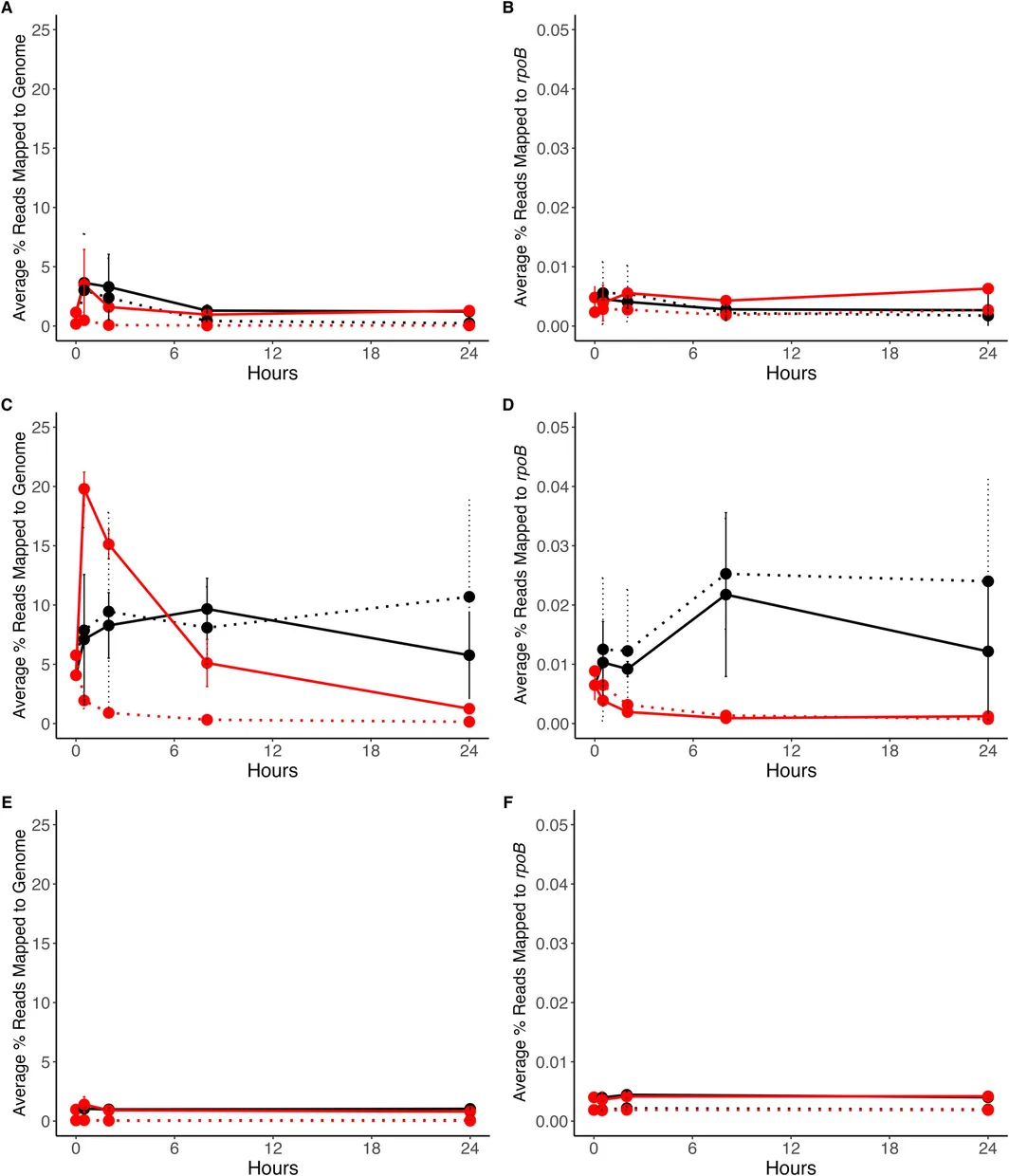
Average percentage of reads mapped to genome (A, C, E) and percentage mapped to *rpoB* gene abundance (B, D, F) from pre‐bloom (A, B, June 2023), peak bloom (C, D, August 2023), and bloom decline (E, F, September 2023). *Microcystis aeruginosa* NIES‐843 is shown with solid lines, and *Dolichospermum* LE20‐WE8 with dotted lines. Control samples are shown in black, and hydrogen peroxide–treated samples (10 μM) are shown in red.

The relative abundance of reads mapped to the *Microcystis aeruginosa* NIES‐843 and *Dolichospermum* sp. LE20‐WE8 genomes was used as a proxy for species‐specific transcriptional responses to a pulse of 10 μM exogenous hydrogen peroxide (Baylous et al. 2024; Den Uyl et al. 2025; Kaneko et al. 2007; Steffen et al. 2015, 2017). During the pre‐bloom and bloom decline seasons, mapped read abundance remained consistently low across timepoints in both control and hydrogen peroxide treated samples, with no statistically significant differences observed (Figure 4; Tables S4 and S5). In the pre‐bloom experiment, *Microcystis* genome mapped reads in treated samples started at 1.1% at 0 h and fluctuated minimally across the time series (Table S4). In bloom decline, total reads mapped to the NIES 843 genome ranged from 0.8% to 1.3% in treated samples, while *Dolichospermum* LE20‐WE8 mapped reads remained below 0.1% throughout (Tables S4 and S5). These patterns indicate minimal transcriptional impact of hydrogen peroxide on these co‐dominant taxa outside peak bloom conditions (Figures 2 and 3).

During peak bloom conditions, *Microcystis* NIES 843 genome mapped read abundance in hydrogen peroxide treated samples increased sharply from 4.1% (0 h) to 19.8% (30 min) and 15.1% at (2 h), then declined to 5.1% (8 h) and 1.3% (24 h) (Figure 4C and Table S4). Despite the pronounced early rise, no significant differences were detected between treatment and control groups or between time points within the treated group, likely due to high variability. Control samples exhibited a smaller increase, peaking at 9.7% (8 h) before decreasing (Table S4). These patterns suggest an early, biologically evident but not statistically supported transcriptional response by *Microcystis* to hydrogen peroxide exposure. For *Dolichospermum* LE20‐WE8, hydrogen peroxide treated samples showed a steady decline in genome‐mapped read abundance from 5.8% (0 h) to 0.9% (2 h) and 0.1% (24 h), whereas control samples remained comparatively stable across the 24 h time series (Figure 4D and Table S5). Reads mapped to the cyanobacterial *rpoB* genes, which encodes the β‐subunit of RNA polymerase and serves as a marker of basal transcription, exhibited similar patterns to global transcriptional patterns with no significant differences observed (Figure 4B,D,F; Tables S6 and S7).

### 3.4 Bacterial Contribution to the Bloom Consortium ROS Response

To determine the bacterial contribution to the bloom microbiome's response to a pulse of exogenous hydrogen peroxide stress, we quantified the relative abundance of genes encoding catalase‐peroxidase/catalase (including *katG*, *katA*, *katE*, *ydbD*), superoxide dismutase (including *sodA*, *sodB*, *sodC*, *sodF*, *sodN*, *sodX*), and alkyl hydroperoxide reductase (including *ahpC‐F*). These genes were selected based on their prior identification in Lake Erie and other freshwater metatranscriptomes, as well as in marine systems (Baylous et al. 2024; Smith et al. 2022).

**FIGURE 5:**
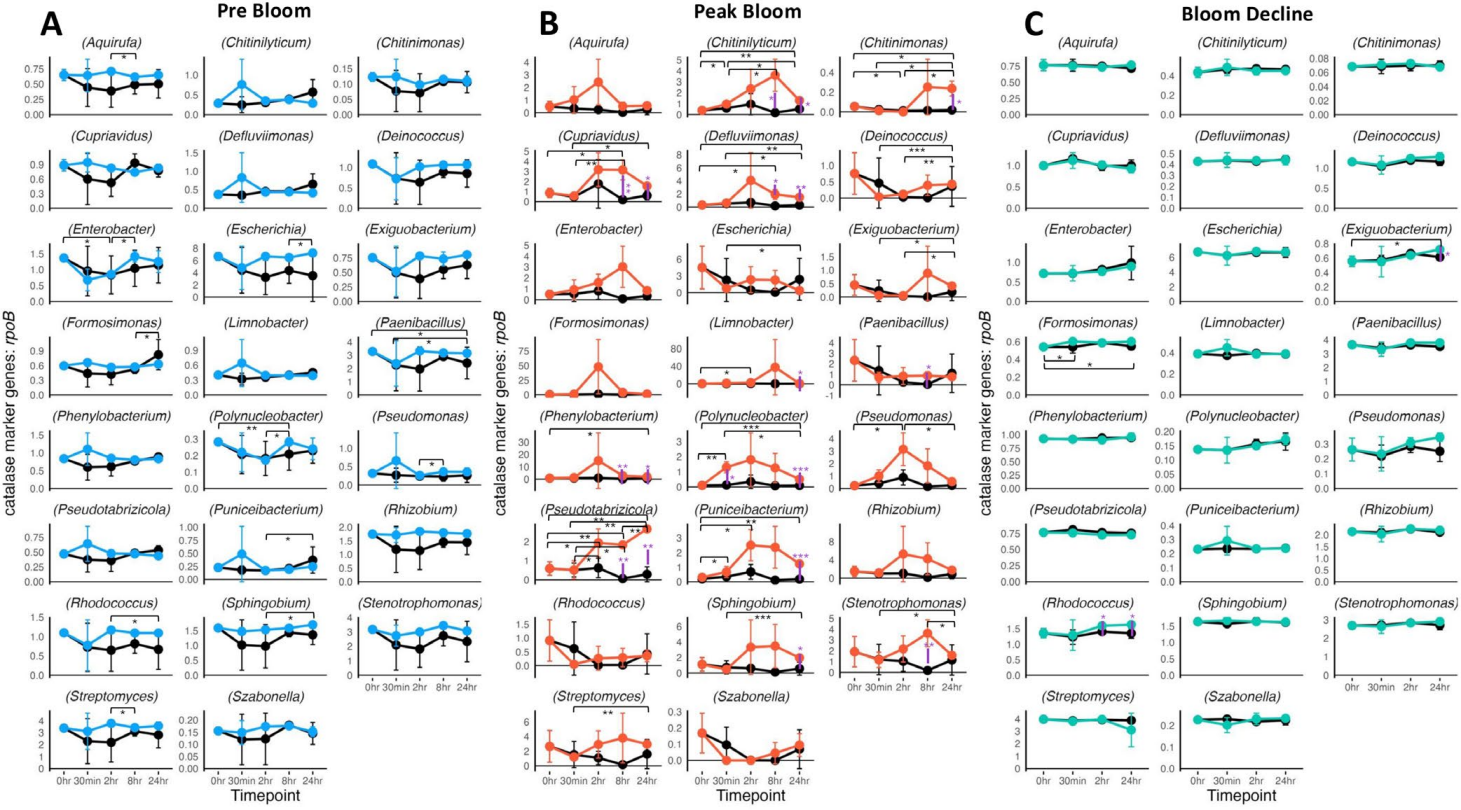
Expression of catalase marker genes over time (*katG*, *katA*, *katE*, *ydbD*). Expression quantified as the ratio of catalase marker gene reads to *rpoB* reads across 23 bacterial genera during the pre‐bloom (A—blue), peak bloom (B—orange) and bloom decline (C—turquoise) sampling periods. Coloured lines represent hydrogen peroxide treated samples, and black lines represent untreated controls. Purple vertical lines indicate significant differences between hydrogen peroxide and control samples, while black horizontal lines indicate significant differences between timepoints with the hydrogen peroxide treatment. Data points reflect the average ratio at each timepoint across biological replicates; error bars represent standard deviation. Asterisks denote genera with statistically significant differences across timepoints (*p < 0.05, **p < 0.005, ***p < 0.005). The 8 h timepoint was not sampled during the September (Bloom Decline) experiment.

In the pre‐bloom season (June), no genera showed significant differences between treatments (control vs. hydrogen peroxide), but 11 genera exhibited significant temporal shifts in expression within the hydrogen peroxide treatment group. *Polynucleobacter* significantly decreased from 0 to 2 h (0.283 to 0.172; *p* = 0.003; Figure 5A and Table S11), followed by a rebound at 8 h. *Formosimonas* significantly increased from 8 to 24 h (0.573 to 0.628; *p* = 0.023; Figure 5A and Table S11), while *Paenibacillus*, *Puniceibacterium*, and *Rhodococcus* also exhibited late‐stage increased catalase marker gene expression from 2 to 24 h (*p* < 0.05; Figure 5A and Table S11), suggesting differential catalase regulation across the time‐series even in the absence of significant treatment‐level differences that were absent in control samples.

In contrast, hydrogen peroxide exposure during peak bloom conditions (August) triggered the most dynamic transcriptional response in catalase marker gene expression by the bacterial genera tested. Of the 23 genera evaluated, 16 (~70%) significantly changed catalase marker gene expression in hydrogen peroxide treated samples compared to control samples at one or more timepoints (Figure 5B and Table S11). These included *Chitinilyticum*, *Chitinimonas*, *Cupriavidus*, *Defluviimonas*, *Limnobacter*, *Paenibacillus*, *Phenylobacterium*, *Polynucleobacter*, *Pseudotabrizicola*, *Puniceibacterium*, *Sphingobium*, and *Stenotrophomonas*. Genera with significant differences between timepoints within the hydrogen peroxide treatment were categorised into three functional response types: first responders, delayed responders, and downregulators, with a fourth category for the non‐statistically significant genera classified as non‐responders (Figure 5B and Table S11). Only five genera were classified as nonresponders: *Aquirufa*, *Enterobacter*, *Formosimonas*, *Rhizobium*, *and Rhodococcus* (Figure 5B).

First responders were members of genera that exhibited an increase in catalase marker gene expression at either the 30 m or 2 h timepoint: *Phenylobacterium*, *Polynucleobacter*, *Pseudomonas*, *Pseudotabrizicola*, and *Puniceibacterium* (Figure 5B). *Phenylobacterium* demonstrated one of the strongest early responses, with catalase marker gene expression ratios increasing from 0.923 to 14.920 at 2 h (*p* = 0.004; Figure 5B and Table S11) and remaining elevated at 8 h (*p* = 0.002; Figure 5B and Table S11). *Polynucleobacter* and *Pseudomonas* both increased expression early, followed by a subsequent decline, suggesting a transient oxidative stress response (Figure 5B and Table S11). *Pseudotabrizicola* had a unique sustained response to elevated hydrogen peroxide, with significantly elevated catalase marker gene expression ratios from 2 h through 24 h (0.471 to 2.659; *p* = 5.86e‐4; Figure 5B and Table S11). *Puniceibacterium* increased from 0.303 to 2.503 at 2 h (*p* = 0.024), with expression still elevated at 24 h (1.232; *p* = 3.69e‐4; Figure 5B and Table S11).

Delayed responders were genera that exhibited an increase in catalase marker gene expression at either the 8 h or 24 h timepoint, with minimal expression at the earlier timepoints: *Chitinilyticum*, *Chitinimonas*, *Defluviimonas*, *Exiguobacterium*, *Streptomyces*, and *Sphingobium* (Figure 5B). *Chitinilyticum* increased from 0.377 at 0 h to 1.283 at 24 h (*p* = 0.020), with additional significant differences between 30 min and 24 h (*p* = 0.034) and between 8 h and 30 min (*p* = 0.035, Table S11). *Defluviimonas* increased from 0.307 to 1.496 over the time course, with significant upregulation at both 8 h and 24 h (*p* = 0.016 and *p* = 0.0003, Table S11). *Sphingobium* increased from 0.553 at 0 h to 1.954 at 24 h (*p* = 0.045, Table S11), marking a clear delayed response (Figure 5B and Table S11).

Downregulators were classified by significant decreases in catalase marker gene expression during the time‐series: *Escherichia*, *Deinococcus*, *and Stenotrophomonas* (Figure 5B). *Escherichia* significantly declined from 30 min to 24 h (*p* = 0.014, Table S11), and *Deinococcus* similarly decreased between 2 and 24 h (*p* = 0.001, Table S11). *Stenotrophomonas* peaked at 8 h and declined significantly by 24 h (*p* = 0.048, Table S11), reflecting a late stage catalase activation pattern.

During bloom decline (September), catalase:*rpoB* ratio responses were markedly subdued compared to peak bloom conditions (Figure 5). Only two genera significantly differentially regulated catalase marker gene expression between treatments: *Exiguobacterium* and *Rhodococcus* (Table S11). Within the hydrogen peroxide time series, *Exiguobacterium* and *Formosimonas* both significantly increased expression at 24 h compared to 0 h (*p* = 0.049 and *p* = 0.010, respectively; Figure 5C and Table S11). However, expression levels during September were generally lower than in August, and the absence of the 8 h timepoint limited resolution of mid‐response patterns.

**FIGURE 6:**
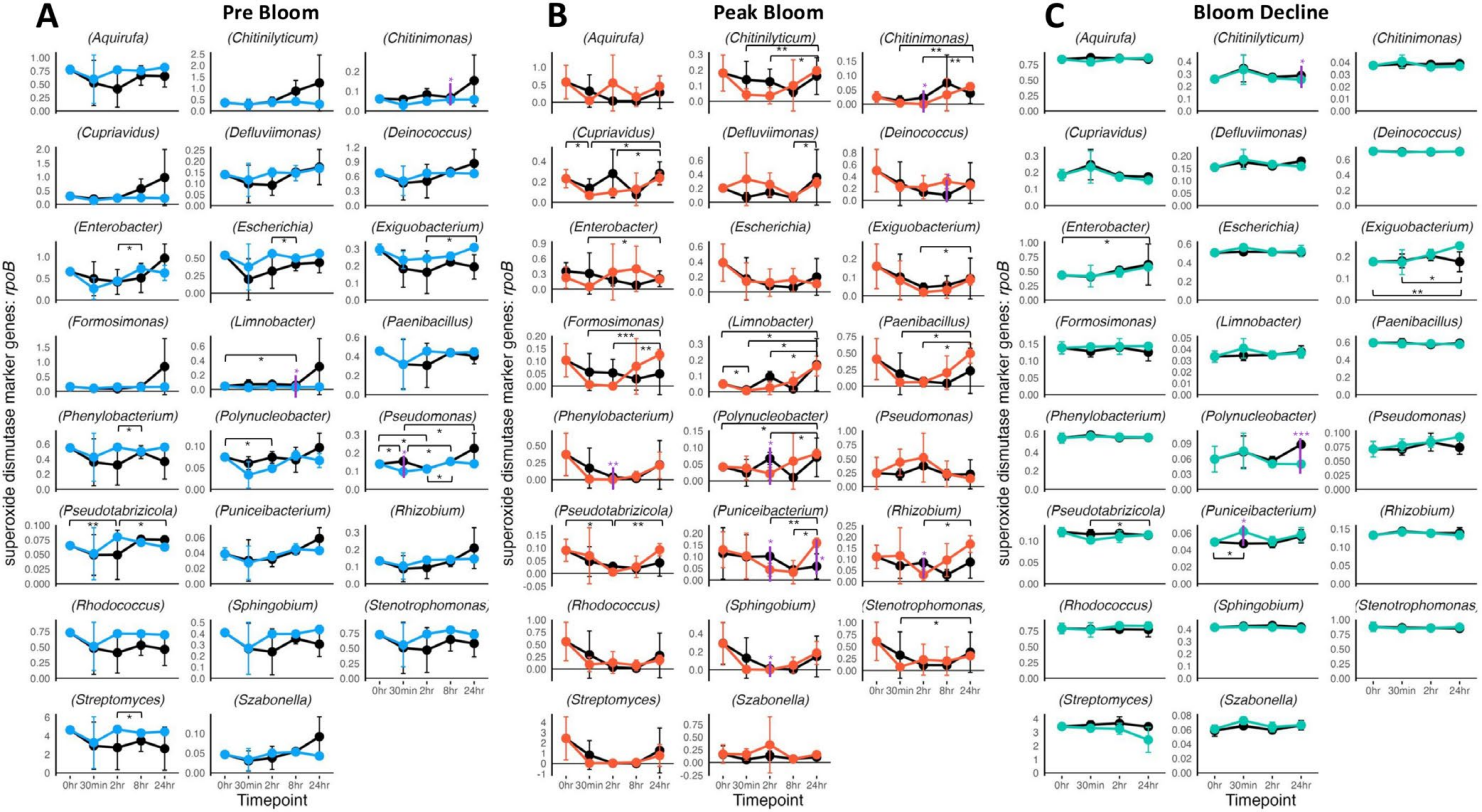
Expression of superoxide dismutase marker genes (*sodA*, *sodB*, *sodC*, *sodF*, *sodN*, *sodX*) over time. Expression quantified as the ratio of catalase marker gene reads to *rpoB* reads across 23 bacterial genera during the P pre‐bloom (A—blue), peak bloom (B—orange) and bloom decline (C—turquoise). Coloured lines represent hydrogen peroxide treated samples, and black lines represent untreated controls. Purple vertical lines indicate significant differences between hydrogen peroxide and control samples, while black horizontal lines indicate significant differences among hydrogen peroxide timepoints. Data points reflect the average ratio at each timepoint across biological replicates; error bars represent standard deviation. Asterisks denote genera with statistically significant differences across timepoints (*p < 0.05, **p < 0.005, ***p < 0.0005). The 8 h timepoint was not sampled during the September (Bloom Decline) experiment.

The transcriptional responses of superoxide dismutase marker genes showed some similar trends in comparison to the catalase marker genes. The response was minimal during pre‐bloom and bloom decline periods, and in some cases hydrogen peroxide treatment resulted in reduced expression compared to the control (Figure 6A and Table S12). During the pre‐bloom period (June), only three genera exhibited significant differences between hydrogen peroxide treated and control samples at individual timepoints: *Chitinimonas*, *Limnobacter*, and *Pseudomonas* (Figure 6A and Table S12). In all three cases, expression in the control samples was significantly higher (Table S12). Within the hydrogen peroxide treatment group, 11 genera exhibited timepoint‐specific differences, including *Chitinilyticum*, *Escherichia*, *Phenylobacterium*, and *Polynucleobacter*, but these responses were largely inconsistent, transient, and lacked a coordinated temporal pattern. In contrast, three genera, *Enterobacter*, *Exiguobacterium*, and *Pseudotabrizicola*, displayed a distinct and consistent trend. Each showed an initial decline in expression followed by a marked increase at 8 h or 24 h, categorising them as ‘delayed responders’ (Figure 6A and Table S12).

In contrast, the peak bloom (August) period showed the clearest differential transcriptional activity, although the response was still relatively weaker than for catalase. Four genera displayed significant differences between hydrogen peroxide treated and control samples at the 2 h or 8 h timepoints: *Chitinimonas*, *Deinococcus*, *Phenylobacterium*, and *Polynucleobacter* (Figure 6B and Table S12). Within the hydrogen peroxide group, 13 genera exhibited significantly elevated SOD marker gene expression at 24 h compared to earlier timepoints, falling into the delayed responders category. This category included *Chitinilyticum*, *Chitinimonas*, *Formosimonas*, *Paenibacillus*, *Exiguobacterium*, *Cupriavidus*, *Limnobacter*, *Pseudotabrizicola*, and *Puniceibacterium* (Figure 6B and Table S12). Although significant, the magnitude of change in these genera remained relatively low. No genera consistently upregulated SOD marker gene expression in a manner characteristic of ‘first responders,’ by increasing expression at the 30 m and 2 h timepoints. The only early response observed was a significant suppression in *Phenylobacterium* at 2 h (*p* = 0.001 Figure 6B and Table S12).

During bloom decline (September), only *Chitinilyticum*, *Polynucleobacter*, and *Puniceibacterium* significantly differentially regulated SOD marker gene expression between hydrogen peroxide treated and control samples (Figure 6C and Table S12). *Exiguobacterium* and *Enterobacter* exhibited significant within‐treatment changes over time, with elevated expression by 24 h (Figure 6C and Table S12).

**FIGURE 7:**
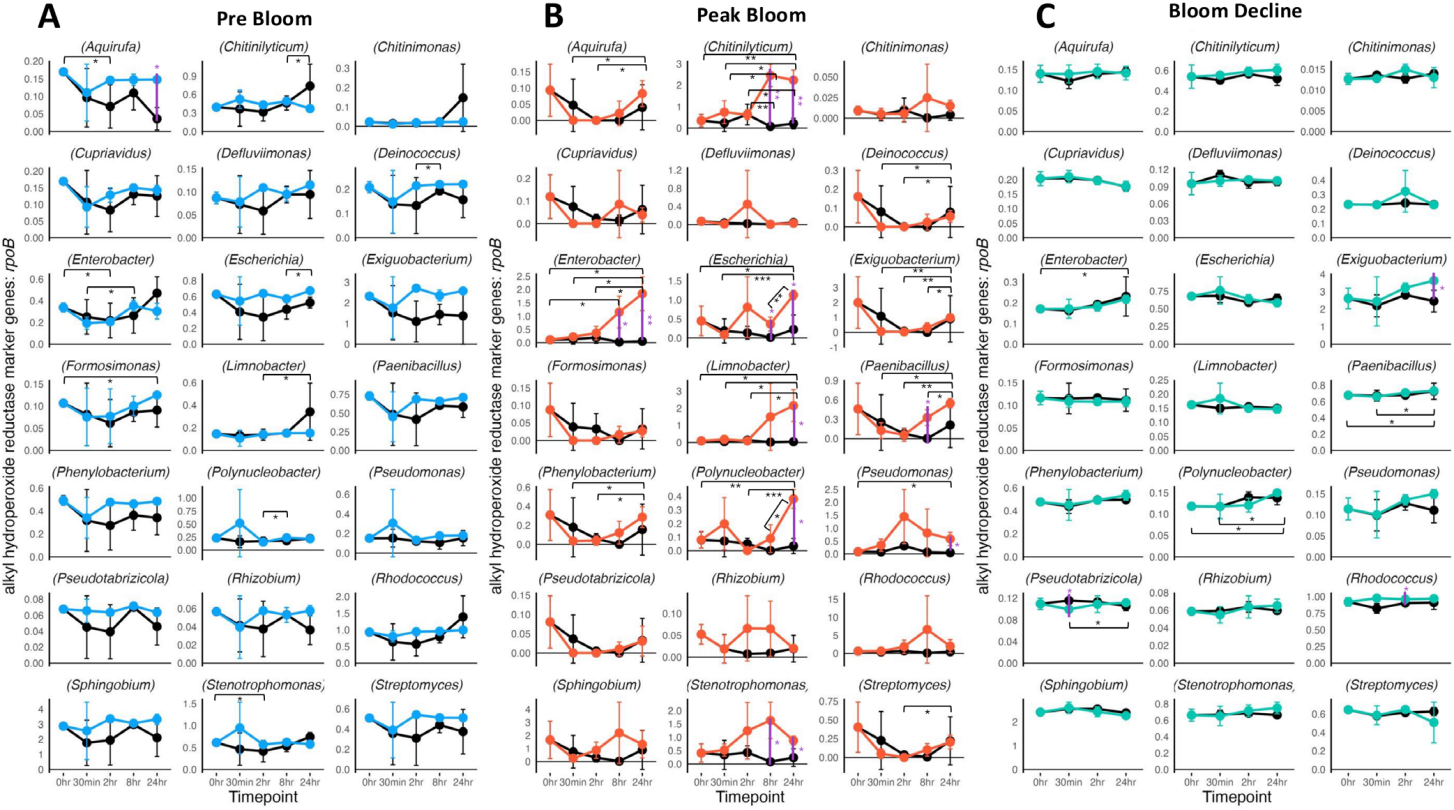
Expression of alkyl hydroperoxide reductase marker genes (*ahpC‐F*) over time. Expression quantified as the ratio of alkyl hydroperoxide reductase gene reads to *rpoB* reads across 21 bacterial genera during the pre‐bloom (A—blue), peak bloom (B—orange) and bloom decline (C—turquoise) sampling periods. *Puniceibacterium* and *Szabonella* were excluded due to the absence of genes encoding alkyl hydroperoxide reductase. Coloured lines represent hydrogen peroxide treated samples, and black lines represent untreated controls. Purple vertical lines indicate significant differences between hydrogen peroxide and control samples, while black horizontal lines indicate significant differences among hydrogen peroxide timepoints. Data points reflect timepoint averages across biological replicates; error bars represent standard deviation. Asterisks denote genera with statistically significant differences across timepoints (*p < 0.05, **p < 0.005, ***p < 0.0005). The 8 h timepoint was not sampled during the September (Bloom Decline) experiment. The figure was created in R (v4.3.1) using the ggplot2 and patchwork packages.

The final group of marker genes evaluated are involved in alkyl hydroperoxide reductase (*ahpC*) production. Among the 23 genera analysed, two did not possess any annotated genes linked to *ahpC* production (*Puniceibacterium* and *Szabonella*) and were therefore not included in the analysis. During pre‐bloom and bloom decline conditions, bacterial transcriptional responses for *ahpC* were limited in both magnitude and frequency but still revealed shifts in gene expression. In June, *Aquirufa* exhibited a significant difference between control (0.036) and hydrogen peroxide treated samples (0.148) at 24 h (*p* = 0.043), suggesting a modest but specific response to oxidative stress (Table S13). In contrast, *Stenotrophomonas* significantly downregulated expression between 0 h (0.611) and 2 h (0.566) (*p* = 0.007; Figure 7A and Table S13), indicating a possible stress‐suppression response. In September, *Exiguobacterium* showed significantly higher expression in hydrogen peroxide treated samples (3.628) compared to control (2.457) at 24 h (*p* = 0.033; Figure 7C and Table S13). *Pseudotabrizicola* slightly decreased expression at 30 min (control: 0.116, hydrogen peroxide: 0.100; *p* = 0.026; Figure 7C and Table S13). Within‐treatment comparisons also showed elevated expression at 24 h for *Enterobacter* (0 h: 0.170, 24 h: 0.217; *p* = 0.049; Figure 7C and Table S13), *Paenibacillus* (0 h: 0.679, 24 h: 0.734; *p* = 0.034; Figure 7C and Table S13), and *Polynucleobacter* (0 h: 0.118, 24 h: 0.150; *p* = 0.023; Figure 7C and Table S13), reflecting late‐stage transcriptional activation. Although overall responses were weaker than those observed during peak bloom, these findings suggest that even during lower biomass periods, select genera mount measurable *ahpC*‐mediated responses to oxidative stress.

Although pre‐bloom and bloom decline seasons exhibited measurable oxidative stress responses, peak bloom conditions elicited the most widespread and sustained transcriptional shifts in *ahpC* expression in response to hydrogen peroxide exposure, with 15 genera exhibiting significant changes (Table S13). First responders included *Chitinilyticum*, *Enterobacter*, *Paenibacillus*, and *Polynucleobacter*, each significantly upregulating *ahpC* expression at either the 30 m or 2 h timepoint In many cases, elevated expression was sustained through 8 h and 24 h (e.g., *Enterobacter*: 24 h [1.843] > 0 h [0.110]; *p* = 0.010; Figure 7B and Table S13), indicating prolonged oxidative stress defence. Delayed responders such as *Exiguobacterium*, *Phenylobacterium*, and *Limnobacter* exhibited minimal early expression but significantly elevated *ahpC* expression at 24 h (e.g., *Exiguobacterium*: 24 h [0.980] > 2 h [0.031]; *p* = 0.003; *Limnobacter*: 24 h [2.145] > 2 h [0.097]; *p* = 0.019; Figure 7B and Table S13). Downregualting genera included *Deinococcus*, *Escherichia*, and *Pseudomonas*, although some of these were transient or showed high variability across replicates.

## 4 Discussion

This study demonstrated that oxidative stress mitigation within *Microcystis* bloom microbial communities is not uniformly distributed across all genera in the bacterial bloom microbiome, but instead is performed by a specific group of bacterial taxa. Bacterial associates of algae play critical roles in nutrient assimilation, vitamin and hormone synthesis, and neutralisation of ROS such as hydrogen peroxide (Baylous et al. 2024; Morris et al. 2011; Seymour et al. 2017; Smith et al. 2022). This is well established for the *Prochlorococcus*‐*Alteromonas* partnership, but there is a growing body of evidence that bacteria decompose hydrogen peroxide in freshwater cHAB systems using cellular machinery that *Microcystis* spp. generally lack, namely catalase enzymes (Morris et al. 2011, 2012; Smith et al. 2022). For *Microcystis* cells, hydrogen peroxide decomposition reflects a nuanced division of labour: cellular modelling indicates that toxin‐producing cells shield enzymes from ambient hydrogen peroxide. At higher hydrogen peroxide concentrations, non‐toxic cells begin to engage peroxiredoxin‐mediated decomposition, collectively protecting the bloom from oxidative stress (Hellweger et al. 2022). The potential contribution of bacteria further complicates the process.

To better understand the role of bacterial constituents of *Microcystis* bloom communities in the decomposition of hydrogen peroxide, we used in situ incubations exposed to a pulse of elevated hydrogen peroxide levels to test: (1) whether the bacterial associates of Lake Erie cHABs differentially regulate their ROS neutralisation enzymes in response to hydrogen peroxide and (2) whether those responses are impacted by bloom stage. Heterotrophic bacterial functional gene analysis revealed enzyme‐specific transcriptional responses among bacterial taxa, emphasising distinct differential regulation patterns for catalase, superoxide dismutase (SOD), and alkyl hydroperoxide reductase that varied among taxa and season in response to elevated hydrogen peroxide. One limitation of this study is that in situ hydrogen peroxide concentrations were not measured throughout the time course. However, previous work demonstrates that hydrogen peroxide decay in freshwater systems follow first‐order kinetics, with rate constants on the order of ~1 h^−1^ under chlorophyll conditions similar to those observed here (Marsico et al. 2015; Vermilyea et al. 2010). Additional field‐based measurements from Lake Erie report hydrogen peroxide decay on the order of 113 ± 24 nM h^−1^ across bloom conditions, with ambient concentrations typically not exceeding ~1 μM (Cory et al. 2016, 2017; Smith et al. 2025). Taken together, these estimates suggest that the 10 μM hydrogen peroxide added in this experiment would have remained elevated during early time points (30 min and 2 h), declined by 8 h, and been effectively depleted by 24 h. These dynamics indicate that early sampling points capture active oxidative stress, whereas later timepoints likely reflect cellular responses during recovery from prior exposure. Consistent with this interpretation, pronounced differences in transcriptional profiles between treatment and control samples, where hydrogen peroxide was the only variable, support the conclusion that oxidative stress response was induced in the treatment group.

During the 2023 Lake Erie bloom, the greatest number of bacterial genera with significant differential expression of catalase marker genes in response to elevated hydrogen peroxide occurred during peak bloom conditions. These responses were grouped into four distinct expression patterns: first responders, delayed responders, downregulators and non‐responders (Figure 5B). We hypothesise that these response patterns may reflect the ability of these bacterial genera to respond immediately to exogenous hydrogen peroxide stress (first responders) versus the indirect response of some of these genera to stress signals from *Microcystis*, rather than directly from elevated exogenous hydrogen peroxide concentrations (delayed responders).

The stage of bloom development had an impact on bacterial differential regulation of catalase marker genes in response to elevated hydrogen peroxide, as pre‐bloom and bloom decline samples matched peak bloom samples in magnitude of the bacterial differential regulation (Figure 5). Given that these response patterns were mainly observed during peak bloom conditions, it may be indicative of a quorum sensing‐type signal that is density dependent and only impacts surrounding bacteria once a certain *Microcystis* cell density threshold has been reached. Quorum sensing capabilities have been well‐documented in multiple *Microcystis* strains, suggesting that the coordination of oxidative stress responses via similar signalling mechanisms is both plausible and consistent with known functional traits (Xu et al. 2024). Bacteria belonging to the first‐responder group (*Phenylobacterium*, Burkholderiaceae family) and the late‐stage defenders (*Sphingobium*, *Streptomyces*, the Burkholderiaceae family) have previously been shown to express catalase genes during freshwater cHAB conditions (Baylous et al. 2024; Smith et al. 2021, 2022). Furthermore, the responses of the bacteria may indeed be cyanobacterial‐host species or even strain specific (Smith et al. 2022). The more nuanced mechanisms that underlie whether these bacteria are expressing these genes in response to hydrogen peroxide or indirectly in response to signalling from individual *Microcystis* strains are unclear, however. A more detailed analysis of transcriptional and metabolite responses to elevated hydrogen peroxide of these individual genera co‐cultured with *Microcystis* is necessary to validate whether chemical signals from *Microcystis* can induce supplementary catalase gene expression by the late‐stage defenders.

In contrast to catalase marker gene expression, differential expression by the target bacterial genera of superoxide dismutase marker genes was limited. Since *Microcystis* encodes its own superoxide dismutase (SOD) genes, community‐level responses were expected to be less pronounced under the experimental conditions. Only a handful of genera demonstrated significant changes in SOD marker gene expression at any stage of bloom development, and these changes were typically modest, delayed, and/or transient (Figure 6). Similarly, alkyl hydroperoxide reductase exhibited a variable and intermediate response. While many genera significantly differentially regulated alkyl hydroperoxide marker gene expression in response to hydrogen peroxide exposure, these were often less consistent or pronounced than catalase (Figures 5 and 7). This pattern is consistent with the intracellular decomposition of the role that alkyl hydroperoxide reductase fills as opposed to extracellular decomposition, as well as *Microcystis's* ability to produce this enzyme itself, making external support from the bacterial community less essential.

The bacteria selected for targeted analysis of ROS‐stress marker gene expression were chosen due to their presence in this dataset (Figure 3) or previous association with *Microcystis* blooms in Lake Erie (Hoke et al. 2021; Smith et al. 2022) or other systems (Baylous et al. 2024; Cook et al. 2020). Notably, *Exiguobacterium* transcripts were universally present in samples from all three stages of bloom development and even outnumbered the Cyanobacteria in some pre‐bloom and bloom‐decline samples (Figure 3). Members of this genus have been previously identified as colony‐inducers for *Microcystis*, indicating that their presence has a direct physiological impact on these cHAB‐forming Cyanobacteria (Akins et al. 2020). Based upon our analysis, however, it is unlikely that the role for *Exiguobacterium* spp. is involved in hydrogen peroxide decomposition, as the maker gene expression response by this genus throughout the 2023 Lake Erie bloom season was limited (Figures 5, 6, 7).

Across all three enzyme groups, a core cohort—*Aquirufa*, *Cupriavidus*, *Formosimonas*, *Stenotrophomonas*, and *Rhodococcus—*consistently showed significant changes in regulation of catalase, superoxide dismutase, and alkyl hydroperoxide reductase. This concentrated functional capacity echoes prior division‐of‐labour patterns in bloom microbiomes and points to coordinated, stage‐dependent protection. Taxon‐specific functional contribution by bacteria hasbeen observed previously for bloom microbial communities, with functions ranging from nitrogen metabolism and assimilation to vitamin synthesis (Baylous et al. 2024; Steffen et al. 2017). We hypothesise that genus‐specific response patterns in response to hydrogen peroxide stress may be related to interaction dynamics and signalling between the bacteria and *Microcystis*, or they may be related to division of labour within the bacterial community, promoting ‘around the clock’ protection for the bloom community while optimising energy expenditure under oxidative stress conditions. The division of labour reduces redundancy, enhances efficiency, and can likely partially account for bloom community resilience under stress. The observed potential contribution of various taxa to hydrogen peroxide decomposition aligns with recent experiments in Lake Erie that indicate multiple free‐living taxa as the biological source of hydrogen peroxide decay during bloom conditions (Smith et al. 2025).

## 5 Conclusions

**FIGURE 8:**
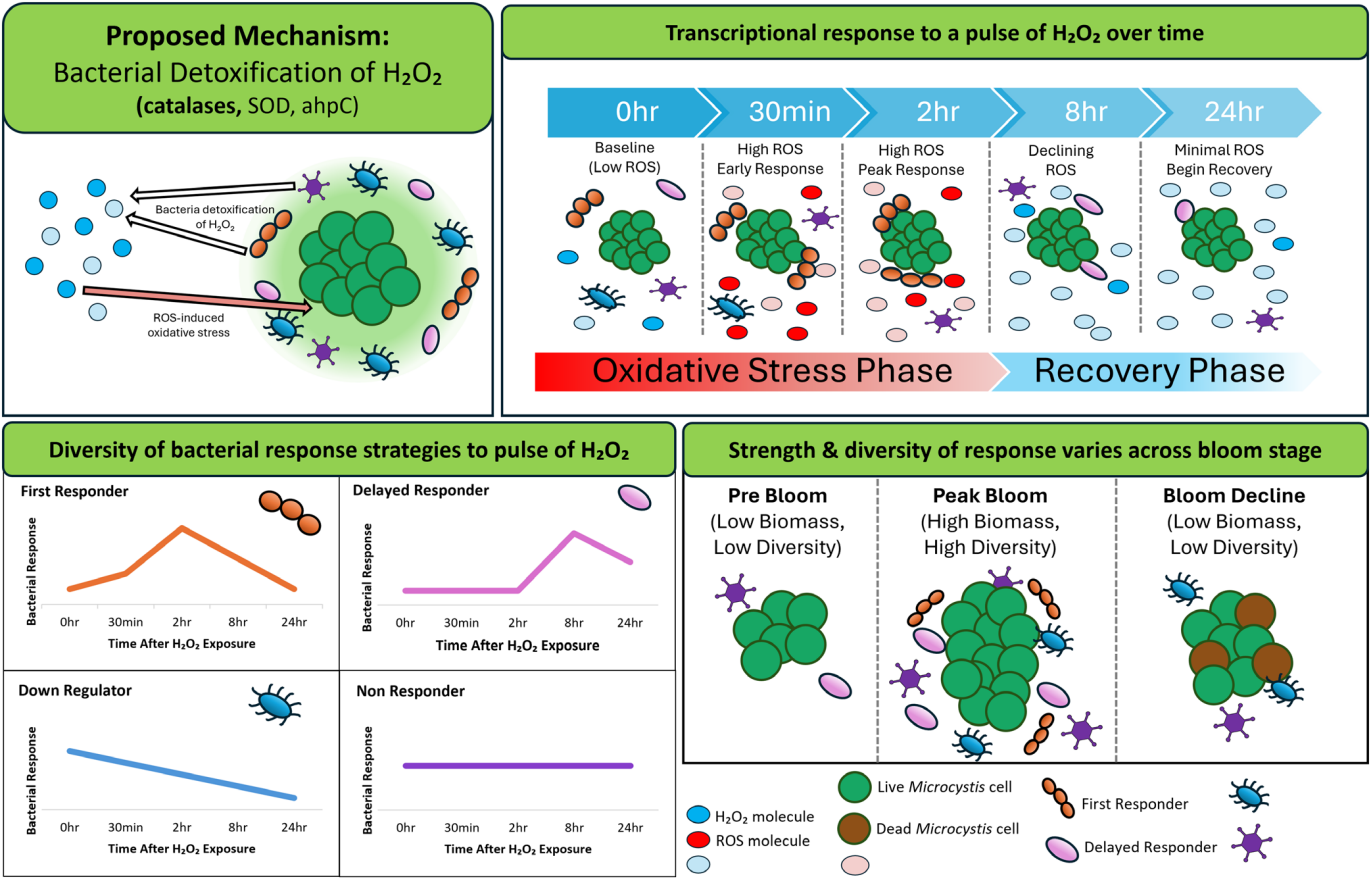
Summary of the expression patterns of the bacterial community of Lake Erie *Microcystis* in response to exposure to elevated exogenous hydrogen peroxide concentrations.

The stage of bloom development, represented by samples collected seasonally in June, August, and late September 2023, had a clear impact on the expression of ROS marker genes by bloom‐associated bacterial genera. The strongest and most diverse bacterial expression patterns occurred during peak bloom conditions, when cyanobacterial biomass, oxygen demand, and oxidative stress are typically highest. In contrast, both pre‐bloom and bloom decline communities exhibited fewer significant differences in ROS response gene expression and less pronounced taxonomic restructuring, suggesting that cooperative oxidative stress defence is most robust and functionally important under peak bloom conditions. This pattern is consistent with prior studies showing that the structure and function of the *Microcystis* phycosphere microbiome are seasonally regulated and is synthesised in Figure 8 (Li et al. 2011; Smith et al. 2021; Wu et al. 2019). During peak bloom, associated bacterial communities often become more functionally specialised, enhancing their roles in nutrient recycling, oxidative stress mitigation, and bloom maintenance (Le et al. 2023; Lezcano et al. 2018). In contrast, early and late bloom phases tend to harbour more taxonomically diverse but functionally flexible microbiomes, with reduced dependency on host‐specific interactions (Kim et al. 2020; Mankiewicz‐Boczek and Font‐Nájera 2022). Our findings reflect this temporal partitioning, demonstrating that cooperative bacterial decomposition of hydrogen peroxide was most active during peak bloom, while responses in pre‐bloom and bloom decline periods were comparatively muted. The impact of seasonality and bloom stage on the bacterial responses to elevated hydrogen peroxide further underscores the importance of bloom‐phase dynamics in shaping functional interactions. Rather than plain coexistence, these microbial interactions reflect strategic coordination and functional specialisation, which could be pivotal to the persistence and success of harmful algal blooms. Our findings highlight the importance of microbial diversity, timing, and division of labour in shaping bloom resilience and offer insight for future approaches to bloom management and mitigation.

## Author Contributions

**Chelsea E. Donovan:** conceptualization, investigation, writing – original draft, methodology, validation, visualization, writing – review and editing, formal analysis, data curation. **Gabriel Padilla:** investigation, validation, methodology, formal analysis, writing – review and editing. **Madison Ashbaugh:** conceptualization, investigation, methodology, validation, writing – review and editing, formal analysis. **Jordan Cibellis:** investigation, methodology, validation, writing – review and editing, formal analysis. **Wilson Ritterpusch:** investigation, methodology, validation, writing – review and editing, formal analysis. **Cameron Tierney:** investigation, methodology, validation, writing – review and editing, formal analysis. **Sophie Bencivenga:** conceptualization, investigation, methodology, validation, writing – review and editing, formal analysis. **Justin D. Chaffin:** conceptualization, investigation, funding acquisition, writing – review and editing, supervision, resources. **Morgan M. Steffen:** conceptualization, investigation, funding acquisition, writing – original draft, methodology, validation, visualization, writing – review and editing, formal analysis, project administration, data curation, supervision, resources. **Louie L. Wurch:** conceptualization, investigation, funding acquisition, writing – original draft, visualization, writing – review and editing, project administration, supervision.

## Funding

This work was supported by National Science Foundation (DEB‐1831106) and National Oceanic and Atmospheric Administration SBIR (NA22OAR0210493).

## Conflicts of Interest

The authors declare no conflicts of interest.

## Supporting information


**Figure S1:** Order‐level composition of bacterial communities in control (A, C, E) and hydrogen peroxide treated (10 μM) samples (B, D, F) from pre‐bloom (A–B, June 2023), peak bloom (C–D, August 2023), and bloom decline (E–F, September 2023). Bars represent the mean relative abundance of each bacterial order across biological replicates at five timepoints (0 h, 30 min, 2 h, 8 h, and 24 h). The 8 h timepoint was not sampled during the September bloom decline (E–F). Taxa with < 1% mean relative abundance across the dataset or lacking classification were grouped as ‘Unclassified/Low‐Abundance.’
**Table S1:** Bacterial genera selected for data mining and their representative freshwater isolates. NCBI RefSeq assembly accession numbers for each isolate are listed with the paper announcement.
**Table S2:** List of enzyme and gene names included in the nucleotide sequence search for each of the four targeted genes.
**Table S3:** Fluorescence measurements of chlorophyll‐*a* concentration obtained using an AquaFluor handheld fluorometer. Values are presented as mean ± standard deviation (SD) from three biological replicates. Paired t‐tests were conducted to assess statistical differences between control and 10 μM hydrogen peroxide treated samples at each timepoint. The same 0 h values were used for both control and treatment comparisons, resulting in a *p* value of 1 at that timepoint. The 8 h timepoint was not performed in September. Statistically significant differences are indicated (* = *p* < 0.05, ** = *p* < 0.005, *** = *p* < 0.0005).
**Table S4:** Average percentage of reads mapped to the *Microcystis aeruginosa* NIES‐843 genome, calculated as the proportion of mapped reads relative to total sample reads. Values are reported as mean ± standard deviation (SD) from biological replicates. No outliers were detected using the Tukey IQR method. Unpaired t‐tests were used to assess: (1) differences between timepoints within 10 μM hydrogen peroxide treated samples, (2) differences between timepoints within control samples, and (3) differences between control and 10 μM hydrogen peroxide treated samples at each timepoint. For the 0 h comparison, the same baseline values were used for both treatment and control groups, resulting in a *p* value of 1. The 8 h timepoint was not collected in September.
**Table S5:** Average percentage of reads mapped to the *Dolichospermum* sp. LE20‐WE8 genome, calculated as the proportion of mapped reads relative to total sample reads. Values are reported as mean ± standard deviation (SD) from biological replicates. No outliers were detected using the Tukey IQR method. Unpaired t‐tests were used to assess: (1) differences between timepoints within 10 μM hydrogen peroxide treated samples, (2) differences between timepoints within control samples, and (3) differences between control and 10 μM hydrogen peroxide treated samples at each timepoint. For the 0 h comparison, the same baseline values were used for both treatment and control groups, resulting in a *p* value of 1. The 8 h timepoint was not collected in September.
**Table S6:** Average percentage of reads mapped to the *rpoB* gene in *Microcystis aeruginosa* NIES‐843, expressed as the proportion of mapped reads relative to total sample reads. Values are reported as mean ± standard deviation (SD) from biological replicates. No outliers were detected using the Tukey IQR method. Unpaired t‐tests were conducted to assess: (1) differences between timepoints within 10 μM hydrogen peroxide treated samples, (2) differences between timepoints within control samples, and (3) differences between control and 10 μM hydrogen peroxide treated samples at each timepoint. The same 0 h values were used for both groups, resulting in a *p* value of 1. The 8 h timepoint was not collected in September.
**Table S7:** Average percentage of reads mapped to the *rpoB* gene in *Dolichospermum* LE20‐WE8, expressed as the proportion of mapped reads relative to total sample reads. Values are reported as mean ± standard deviation (SD) from biological replicates. No outliers were detected using the Tukey IQR method. Unpaired t‐tests were conducted to assess: (1) differences between timepoints within 10 μM hydrogen peroxide treated samples, (2) differences between timepoints within control samples, and (3) differences between control and 10 μM hydrogen peroxide treated samples at each timepoint. The same 0 h values were used for both groups, resulting in a *p* value of 1. The 8 h timepoint was not collected in September.
**Table S8:** Mean relative abundance of Cyanobacteria phyla across timepoints during the three sampling periods. Values are reported as mean ± standard deviation (SD) based on biological replicates. Outliers were removed using the Tukey IQR method. Unpaired t‐tests were conducted to assess: (1) differences between timepoints within 10 μM hydrogen peroxide‐treated samples, (2) differences between timepoints within control samples, and (3) differences between hydrogen peroxide treated and control samples at each timepoint. Statistical significance is annotated with asterisks (* = *p* < 0.05, ** = *p* < 0.005, *** = *p* < 0.0005). For the 0 h comparison, the same baseline values were used for both treatment and control groups, resulting in a *p* value of 1. The 8 h timepoint was not collected in September.
**Table S9:** Mean relative abundance of the *Microcystis* genus within the total microbial community across all timepoints during the three sampling trips. Values are reported as mean ± standard deviation (SD) from biological replicates. Outliers were removed using the Tukey IQR method. Unpaired t‐tests were conducted to assess: (1) differences between timepoints within 10 μM hydrogen peroxide treated samples, (2) differences between timepoints within control samples, and (3) differences between hydrogen peroxide treated and control samples at each timepoint. Statistical significance is annotated with asterisks (* = *p* < 0.05, ** = *p* < 0.005, *** = *p* < 0.0005). For the 0 h comparison, the same baseline values were used for both treatment and control, resulting in a *p* value of 1. The 8 h timepoint was not collected in September.
**Table S10:** Mean relative abundance of the *Dolichospermum* genus within the total microbial community across all timepoints during the three sampling trips. Values are reported as mean ± standard deviation (SD) from biological replicates. Outliers were removed using the Tukey IQR method. Unpaired t‐tests were conducted to assess: (1) differences between timepoints within 10 μM hydrogen peroxide treated samples, (2) differences between timepoints within control samples, and (3) differences between hydrogen peroxide treated and control samples at each timepoint. Statistical significance is annotated with asterisks (* = *p* < 0.05, ** = *p* < 0.005, *** = *p* < 0.0005). For the 0 h comparison, the same baseline values were used for both treatment and control, resulting in a *p* value of 1. The 8 h timepoint was not collected in September.
**Table S11:** Catalase marker gene expression across June, August, and September sampling seasons, reported as the mean ± standard deviation of the catalase marker gene:*rpoB* ratio. Unpaired *t*‐tests were used to assess statistical differences across three comparison types: between treatments at each timepoint (hydrogen peroxide vs. control), within the hydrogen peroxide treatment time series, and within the control time series. Significance thresholds are indicated as follows: *p* < 0.05 = *, *p* < 0.005 = **, *p* < 0.0005 = ***. Note: the September time series does not include an 8‐h timepoint. Significant differences within the hydrogen peroxide treatment group marked with ‘(R)’ were excluded from interpretation, as they were also observed in the control group and likely reflect time‐of‐day effects rather than treatment‐specific responses.
**Table S12:** Superoxide dismutase marker gene expression across June, August, and September sampling seasons, reported as the mean ± standard deviation of the superoxide dismutase marker gene:*rpoB* ratio. Unpaired *t*‐tests were used to assess statistical differences across three comparison types: between treatments at each timepoint (hydrogen peroxide vs. control), within the hydrogen peroxide treatment time series, and within the control time series. Significance thresholds are indicated as follows: *p* < 0.05 = *, *p* < 0.005 = **, *p* < 0.0005 = ***. Note: the September time series does not include an 8‐h timepoint.
**Table S13:** Alkyl hydroperoxide reductase marker gene expression across June, August, and September sampling seasons, reported as the mean ± standard deviation of the alkyl hydroperoxide reductase marker gene:*rpoB* ratio. Unpaired *t*‐tests were used to assess statistical differences across three comparison types: between treatments at each timepoint (hydrogen peroxide vs. control), within the hydrogen peroxide treatment time series, and within the control time series. Significance thresholds are indicated as follows: *p* < 0.05 = *, *p* < 0.005 = **, *p* < 0.0005 = ***. Note: the September time series does not include an 8‐h timepoint. *Puniceibacterium* and *Szabonella* were excluded due to the absence of genes encoding for production of this enzyme.

## Data Availability

Sequence data are available under NCBI accession number PRJNA1282059.
