# Supplementary material for: Bacterial Contribution to Hydrogen Peroxide Decomposition in Lake Erie Cyanobacterial Harmful Algal Bloom Communities Is Dependent on Bloom Stage
Source: Environ Microbiol Rep. 2026 Sep 22;18(5):e70405. doi: 10.1111/1758-2229.70405 (PMC13594773; doi:10.1111/1758-2229.70405)
Supplement: Supplementary file 1 [file EMI4-18-e70405-s001.docx]

# Supplemental Figures

**Figure S1.** Order-level composition of bacterial communities in control (A, C, E) and hydrogen peroxide treated (10 µM) samples (B, D, F) from pre-bloom (A–B, June 2023), peak bloom (C–D, August 2023), and bloom decline (E–F, September 2023). Bars represent the mean relative abundance of each bacterial order across biological replicates at five timepoints (0 hr, 30 min, 2 hr, 8 hr, and 24 hr). The 8 hr timepoint was not sampled during the September bloom decline (E–F). Taxa with <1% mean relative abundance across the dataset or lacking classification were grouped as “Unclassified/Low-Abundance.”

# Supplemental Tables

**Table S1:** Bacterial genera selected for data mining and their representative freshwater isolates. NCBI RefSeq assembly accession numbers for each isolate are listed with the paper announcement.

| **Genus** | **Species (Strain)** | **NCBI RefSeq Assembly #** | **Source** |
| --- | --- | --- | --- |
| *Aquirufa* (2) | *Aquirufa antheringensis* (30S-ANTBAC) | GCF_004307425.1 | ([Pitt *et al.*, 2019)](https://www.zotero.org/google-docs/?UGOccp) |
|  | *Aquirufa antheringensis* (103A-SOEBACH) | GCF_004307415.1 | ([Pitt *et al*., 2019)](https://www.zotero.org/google-docs/?wNaMHF) |
| *Chitinilyticum* (1) | *Chitinilyticum litopenaei*  (DSM 21440) | GCF_000428145.1 | [(Chang *et al*., 2009)](https://www.zotero.org/google-docs/?ZqETzS) |
| *Chitinimonas* (1) | *Chitinimonas sp.* (BJB300) | GCF_006151725.2 | [(Jude, 2019)](https://www.zotero.org/google-docs/?7xjT6g) |
| *Cupriavidus* (1) | *Cupriavidus necator* (H16) | GCF_004798725.1 | [(Kurth *et al*., 2019)](https://www.zotero.org/google-docs/?0f4ywM) |
| *Defluviimonas* (1) | *Defluviimonas aquaemixtae* (CECT 8626) | GCF_900302475.1 | [(Jung *et al.*, 2014)](https://www.zotero.org/google-docs/?MEQJ7T) |
| *Deinococcus* (7) | *Deinococcus aquaticus* (PB-314) | GCF_028622095.1 | [(Im *et al.,* 2008)](https://www.zotero.org/google-docs/?j7D33j) |
|  | *Deinococcus caeni*  (NBRC 10132) | GCF_039544935.1 | [(Im *et al*., 2008)](https://www.zotero.org/google-docs/?d4idXY) |
|  | *Deinococcus aestuarii*  (SYSU M49105) | GCF_018863415.1 | ([Yin *et al.,* 2022)](https://www.zotero.org/google-docs/?Eqq2uJ) |
|  | *Deinococcus koreensis* (SJW1-2) | GCF_002901445.1 | [(Baek *et al.*, 2018)](https://www.zotero.org/google-docs/?TbKgUL) |
|  | *Deinococcus indicus* (DR1) | GCF_002198095.1 | [(Chauhan *et al.*, 2017)](https://www.zotero.org/google-docs/?iEfnwy) |
|  | *Deinococcus roseus*  (JCM 14370) | GCF_014646895.1 | [(Asker *et al.*, 2008)](https://www.zotero.org/google-docs/?sBQcgo) |
|  | *Deinococcus sp.* (JMULE3) | GCF_013337115.1 | [(Hoke *et al*., 2021)](https://www.zotero.org/google-docs/?6SlB9S) |
| *Enterobacter* (13) | *Enterobacter asburiae* (TZW01-TZW11) | TZW01 - GCF_016771675.1  TZW02 - GCF_016771105.1  TZW03 - GCF_016771735.1  TZW04 - GCF_016771095.1  TZW05 - GCF_016771805.1  TZW06 - GCF_016771775.1  TZW07 - GCF_016771715.1  TZW08 - GCF_016771055.1  TZW09 - GCF_016771035.1  TZW10 - GCF_016771835.1  TZW11 - GCF_016771015.1 | [(Leister & Hügler, 2022)](https://www.zotero.org/google-docs/?qRcTrk) |
|  | *Enterobacter sp.* (JMULE2) | GCF_013337145.1 | [(Hoke *et al*., 2021)](https://www.zotero.org/google-docs/?2YYfDG) |
|  | *Enterobacter roggenkampii* (RX.G5M56) | GCF_025021485.1 | [(Xu *et al.*, 2023)](https://www.zotero.org/google-docs/?4ZUG4n) |
| *Escherichia* (2) | *Escherichia coli* (ST131) | GCF_955652485.1 | [(Colomer-Lluch *et al.*, 2013)](https://www.zotero.org/google-docs/?THCv7t) |
|  | *Escherichia coli* (ST69) | GCF_024662075.1 | [(Colomer-Lluch *et al*., 2013)](https://www.zotero.org/google-docs/?zaZzeb) |
| *Exiguobacterium* (2) | *Exiguobacterium profundum* (TSS-3) | GCF_029026745.1 | [(Rincón-Rosales *et al*., 2023)](https://www.zotero.org/google-docs/?HtukP1) |
|  | *Exiguobacterium sp.* (JMULE1) | GCF_013337175.1 | [(Hoke *et al*., 2021)](https://www.zotero.org/google-docs/?vDTLz2) |
| *Formosimonas* (1) | *Formosimonas limnophila* (KCTC 32501) | GCF_014652675.1 | ([Chen *et al.*, 2017)](https://www.zotero.org/google-docs/?vbP3J2) |
| *Limnobacter* (1) | *Limnobacter parvus* (YS8-69) | GCF_024623725.1 | [(Xamxidin *et al.*, 2022)](https://www.zotero.org/google-docs/?0NuZC4) |
| *Paenibacillus* (7) | *Paenibacillus sedimenti* (WST5) | ASM1453465v1 | [(Yan *et al.*, 2023)](https://www.zotero.org/google-docs/?l0OM4b) |
|  | *Paenibacillus allorhizosphaerae* (JJ-447) | GCF_910594985.1 | [(Kämpfer *et al.*, 2021)](https://www.zotero.org/google-docs/?DAcRJU) |
|  | *Paenibacillus sp.* (JMULE4) | GCF_013337105.1 | [(Hoke *et al.*, 2021)](https://www.zotero.org/google-docs/?wAAeOL) |
|  | *Paenibacillus lautus* (BHU3) | GCF_002407025.1 | [(Yadav & Dubey, 2018)](https://www.zotero.org/google-docs/?NO3w7P) |
|  | *Paenibacillus lutrae* (N10) | GCF_009757775.1 | [(Rodríguez *et al*., 2019)](https://www.zotero.org/google-docs/?LTsgMS) |
|  | *Paenibacillus taihuensis* (CGMCC 1.10966) | GCF_003386535.1 | [(Wu *et al.*, 2013)](https://www.zotero.org/google-docs/?BwJODV) |
|  | *Paenibacillus rigui* (JCM 16352) | GCF_002234615.1 | [(Baik *et al.,* 2011)](https://www.zotero.org/google-docs/?sa0lPv) |
| *Phenylobacterium* (2) | *Phenylobacterium parvum* (HYN0004) | GCF_003150835.1 | [(Baek *et al*., 2019)](https://www.zotero.org/google-docs/?V9o0uc) |
|  | *Phenylobacterium glaciei* (20VBR1) | GCF_016772415.1 | [(Thomas *et al*., 2022)](https://www.zotero.org/google-docs/?HyjfqQ) |
| *Polynucleobacter* (3) | *Polynucleobacter sphagniphilus* (MWH-Weng1-1) | GCF_001953355.1 | [(Hahn *et al*., 2017)](https://www.zotero.org/google-docs/?xYvnrQ) |
|  | *Polynucleobacter acidiphobus* (MWH-PoolGreenA3) | GCF_003065385.1 | [(Hahn *et al*., 2011)](https://www.zotero.org/google-docs/?5I6sSO) |
|  | *Polynucleobacter meluiroseus* (AP-Melu-1000-B4) | GCF_900205755.1 | [(Pitt *et al*., 2018)](https://www.zotero.org/google-docs/?QM1hLk) |
| *Pseudomonas* (2) | *Pseudomonas sp.* (ML96) | GCF_000761545.1 | [(Li *et al.*, 2015)](https://www.zotero.org/google-docs/?MhE9lE) |
|  | *Pseudomonas boanensis*  (DB1) | GCF_018704125.1 | ([Nicklasson *et al.*, 2022)](https://www.zotero.org/google-docs/?saQSgL) |
| *Pseudotabrizicola* (1) | *Pseudotabrizicola alkalilacus* (DJC) | GCF_003443995.1 | [(Ma *et al.*, 2022; Phurbu *et al.*, 2019)](https://www.zotero.org/google-docs/?1z6yix) |
| *Puniceibacterium* (1) | *Puniceibacterium confluentis* (JSSK-17) | GCF_007923445.1 | [(Park *et al.,* 2017)](https://www.zotero.org/google-docs/?aCr1J6) |
| *Rhizobium* (3) | *Rhizobium sp.* (CSW-27) | GCF_018760785.1 | [(Chen *et al*., 2021)](https://www.zotero.org/google-docs/?gLbtpc) |
|  | *Rhizobium alvei* (TNR-22) | GCF_030551875.1 | [(Sheu *et al*., 2015)](https://www.zotero.org/google-docs/?4ax5zr) |
|  | *Rhizobium aquaticum*  (DSM 29780) | GCF_040545545.1 | [(Máthé *et al.*, 2018)](https://www.zotero.org/google-docs/?ESpidi) |
| *Rhodococcus* (1) | *Rhodococcus sp.* (ABRD24) | GCF_004328705.1 | (Jin *et. al*., 2019) |
| *Sphingobium* (5) | *Sphingobium limneticum*  (301 - DSM 25076) | GCF_008710155.1 | [(Chen *et al*., 2013)](https://www.zotero.org/google-docs/?ZRysYQ) |
|  | *Sphingobium boeckii*  (469 - DSM 25079) | GCF_014199215.1 | [(Chen *et al*., 2013)](https://www.zotero.org/google-docs/?BT2RxZ) |
|  | *Sphingobium cyanobacteriorum* (HBC34) | GCF_030580935.1 | [(Le *et al*., 2024)](https://www.zotero.org/google-docs/?PxialR) |
|  | *Sphingobium sufflavum* (HL-25) | GCF_021403115.1 | [(Sheu *et al*., 2013)](https://www.zotero.org/google-docs/?58YI28) |
|  | *Sphingobium algorifonticola* (TLA-22) | GCF_004005485.1 | [(Chen *et al*., 2020)](https://www.zotero.org/google-docs/?vf4Ixk) |
| *Stenotrophomonas* (2) | *Stenotrophomonas lacuserhaii* (K32) | GCF_014596295.1 | [(Deng *et al.*, 2022)](https://www.zotero.org/google-docs/?yI33VI) |
|  | *Stenotrophomonas indicatrix* (DAIF1) | GCF_004551575.1 | [(Friedrich *et al.,* 2021)](https://www.zotero.org/google-docs/?nr467O) |
| *Streptomyces* (4) | *Streptomyces meridianus* (MTZ3.1) | GCF_023703325.1 | ([Santos *et al.*, 2023)](https://www.zotero.org/google-docs/?GgV6DK) |
|  | *Streptomyces sp.* (MNU77) | GCF_000935135.2 | [(Mehdiratta *et al.,* 2022)](https://www.zotero.org/google-docs/?iTIcrT) |
|  | *Streptomyces sp.* (BPSDS2) | GCF_005234185.1 | ([Zothanpuia *et al.,* 2019)](https://www.zotero.org/google-docs/?IKWhb4) |
|  | *Streptomyces avermitilis* (MA-4680) | GCF_000009765.2 | [(Ōmura *et al.*, 2001)](https://www.zotero.org/google-docs/?fqu8A1) |
| *Szabonella* (1) | *Szabonella alba* (DMG-N-6) | GCF_016765775.1 | [(Szuróczki *et al.*, 2022)](https://www.zotero.org/google-docs/?94MlIo) |

**Table S2.** List of enzyme and gene names included in the nucleotide sequence search for each of the four targeted genes.

| **Enzyme** | **Names Included in Search** |
| --- | --- |
| Catalase-peroxidase | Catalase/peroxidase HPI (*katG*)  Catalase HPII (*katE*)  Manganese catalase family protein  Catalase family protein  Catalase  Vegetative catalase (*katA*)  Catalase X (*katX*)  Catalase family peroxidase  Putative manganese catalase (*ydbD*)  Putative catalase |
| Superoxide dismutase | Superoxide dismutase  Superoxide dismutase family protein  Superoxide dismutase [Fe] (*sodB*)  Superoxide dismutase [Mn] (*sodA*)  Superoxide dismutase [Cu-Zn] SodC2 (*sodC*)  Fe-Mn family superoxide dismutase  Cu-Zn family superoxide dismutase  Superoxide dismutase [Mn/Fe]  Nickel-type superoxide dismutase maturation protease (*sodX*)  Putative superoxide dismutase (*sodN* & *sodF*) |
| Alkyl hydroperoxide reductase | Alkyl hydroperoxide reductase  Alkylhydroperoxidase  Alkyl hydroperoxide reductase subunit F (*ahpF*)  Alkyl hydroperoxide reductase subunit C (*ahpC*)  Alkyl hydroperoxide reductase subunit E (*ahpE*)  Alkyl hydroperoxide reductase subunit D (*ahpD*)  Peroxiredoxin - alkyl hydroperoxide reductase subunit C (*ahpC*) |
| *rpoB* | DNA-directed RNA polymerase subunit beta |

**Table S3.** Fluorescence measurements of chlorophyll-*a* concentration obtained using an AquaFluor handheld fluorometer. Values are presented as mean ± standard deviation (SD) from three biological replicates. Paired t-tests were conducted to assess statistical differences between control and 10 µM hydrogen peroxide treated samples at each timepoint. The same 0 hr values were used for both control and treatment comparisons, resulting in a p-value of 1 at that timepoint. The 8 hr timepoint was not performed in September. Statistically significant differences are indicated (* = *p* < 0.05, ** = *p* < 0.005, *** = *p* < 0.0005).

| **Chl *a* Concentration (Mean ± SD, RFU)** | | | | | | | | | |
| --- | --- | --- | --- | --- | --- | --- | --- | --- | --- |
| **Month** | **Pre Bloom** | | | **Peak Bloom** | | | **Bloom Decline** | | |
| **Time** | **Control** | **H2O2** | **T-test p-value** | **Control** | **H2O2** | **T-test p-value** | **Control** | **H2O2** | **T-test p-value** |
| 0hr | 16.2333 ± 9.621 | 16.2333 ± 9.621 | 1.00000 | 14.8933 ± 4.738 | 14.8933 ± 4.738 | 1.00000 | 16.2333± 9.621 | 16.2333± 9.621 | 1.00000 |
| 30m | 15.8233 ± 5.935 | 11.0567 ± 9.145 | 0.49100 | 12.003 ± 2.207 | 12.413 ± 1.366 | 0.79800 | 15.8233 ± 5.935 | 11.0567 ± 9.145 | 0.49100 |
| 2hr | 19.6133 ± 5.412 | 8.3367 ± 3.399 | 0.03780 * | 10.5100 ± 2.393 | 11.8700 ± 3.850 | 0.63100 | 13.8233 ± 2.422 | 10.6200 ± 2.967 | 0.03780  * |
| 8hr | 13.8233 ± 2.421 | 10.6200  ± 2.968 | 0.22100 | 16.253 ± 6.504 | 11.863 ± 1.797 | 0.32300 | - | - | - |
| 24hr | 12.6833 ± 6.082 | 1.5533 ± 0.040 | 0.03390 * | 14.557 ± 0.571 | 5.610 ± 1.164 | 0.00028 *** | 12.6833 ± 6.082 | 1.5533 ± 0.040 | 0.03390* |

**Table S4**. Average percentage of reads mapped to the *Microcystis aeruginosa* NIES-843 genome, calculated as the proportion of mapped reads relative to total sample reads. Values are reported as mean ± standard deviation (SD) from biological replicates. No outliers were detected using the Tukey IQR method. Unpaired t-tests were used to assess: (1) differences between timepoints within 10 µM hydrogen peroxide treated samples, (2) differences between timepoints within control samples, and (3) differences between control and 10 µM hydrogen peroxide treated samples at each timepoint. For the 0 hr comparison, the same baseline values were used for both treatment and control groups, resulting in a p-value of 1. The 8 hr timepoint was not collected in September.

| **Pre Bloom Reads Mapped to NIES-843 Genome Percentage** | | | | | |
| --- | --- | --- | --- | --- | --- |
| **Time** | **Control Abundance** | **H2O2 Abundance** | **T-Test Results (Comparing Treatments)** | **Significant**  **T-Test Results (Comparing H2O2)** | **Significant**  **T-Test Results (Comparing Control)** |
| 0hr | 1.10% ±  0.30% | 1.10% ±  0.30% | 1.000 | No significant results | No significant results |
| 30m | 3.60% ±  4.10% | 3.50% ±  3.00% | 0.081 |  |  |
| 2hr | 3.30% ±  2.70% | 1.60% ±  0.20% | 0.081 |  |  |
| 8hr | 1.30% ±  0.50% | 1.00% ±  0.02% | 0.190 |  |  |
| 24hr | 1.20% ±  0.30% | 1.30% ±  0.30% | 0.190 |  |  |
| **Peak Bloom Reads Mapped to NIES-843 Genome Percentage** | | | | | |
| **Time** | **Control Abundance** | **H2O2 Abundance** | **T-Test Results (Comparing Treatments)** | **Significant**  **T-Test Results (Comparing H2O2)** | **Significant**  **T-Test Results (Comparing Control)** |
| 0hr | 4.10% ±  4.80% | 4.10% ±  4.80% | 1.000 | No significant results | No significant results |
| 30m | 7.10% ±  5.50% | 19.80% ±  1.40% | 0.663 |  |  |
| 2hr | 8.30% ±  2.80% | 15.10% ±  1.22% | 0.386 |  |  |
| 8hr | 9.70% ±  2.60% | 5.10% ±  2.00% | 0.699 |  |  |
| 24hr | 5.80% ±  3.70% | 1.30% ±  1.30% | 1.000 |  |  |
| **Bloom Decline Reads Mapped to NIES-843 Genome Percentage** | | | | | |
| **Time** | **Control Abundance** | **H2O2 Abundance** | **T-Test Results (Comparing Treatments)** | **Significant**  **T-Test Results (Comparing H2O2)** | **Significant**  **T-Test Results (Comparing Control)** |
| 0hr | 1.00% ±  0.10% | 1.00% ±  0.10% | 1.000 | No significant results | No significant results |
| 30m | 1.00% ±  0.10% | 1.40% ±  0.70% | 1.000 |  |  |
| 2hr | 1.00% ±  0.10% | 0.90% ±  0.10% | 0.663 |  |  |
| 24hr | 1.00% ±  0.20% | 0.80% ±  0.10% | 0.190 |  |  |

**Table S5:** Average percentage of reads mapped to the *Dolichospermum sp.* LE20-WE8 genome, calculated as the proportion of mapped reads relative to total sample reads. Values are reported as mean ± standard deviation (SD) from biological replicates. No outliers were detected using the Tukey IQR method. Unpaired t-tests were used to assess: (1) differences between timepoints within 10 µM hydrogen peroxide treated samples, (2) differences between timepoints within control samples, and (3) differences between control and 10 µM hydrogen peroxide treated samples at each timepoint. For the 0 hr comparison, the same baseline values were used for both treatment and control groups, resulting in a p-value of 1. The 8 hr timepoint was not collected in September.

| **Pre Bloom Reads Mapped to LE20-WE8 Genome Percentage** | | | | | |
| --- | --- | --- | --- | --- | --- |
| **Time** | **Control Abundance** | **H2O2 Abundance** | **T-Test Results (Comparing Treatments)** | **Significant**  **T-Test Results (Comparing H2O2)** | **Significant**  **T-Test Results (Comparing Control)** |
| 0hr | 0.20% ±  0.11% | 0.20% ±  0.11% | 1.000 | No significant results | No significant results |
| 30m | 3.00% ±  4.80% | 0.50% ±  0.68% | 0.663 |  |  |
| 2hr | 2.40% ±  3.30% | 0.10% ±  0.03% | 0.663 |  |  |
| 8hr | 0.40% ±  0.07% | 0.04% ±  0.01% | 0.081 |  |  |
| 24hr | 0.20% ±  0.22% | 0.04% ±  0.01% | 0.081 |  |  |
| **Peak Bloom Reads Mapped to LE20-WE8 Genome Percentage** | | | | | |
| **Time** | **Control Abundance** | **H2O2 Abundance** | **T-Test Results (Comparing Treatments)** | **Significant**  **T-Test Results (Comparing H2O2)** | **Significant**  **T-Test Results (Comparing Control)** |
| 0hr | 0.058 ±  9.1e-2 | 0.058 ±  9.1e-2 | 1.000 | No significant results | No significant results |
| 30m | 0.079 ±  8.7e-2 | 0.019 ±  7.0e-3 | 0.663 |  |  |
| 2hr | 0.094 ±  8.4e-2 | 0.009 ±  1.9e-3 | 0.149 |  |  |
| 8hr | 0.081 ±  3.4e-2 | 0.003 ±  2.4e-4 | 0.245 |  |  |
| 24hr | 0.107 ±  8.2e-2 | 0.001 ±  2.3e-3 | 0.245 |  |  |
| **Bloom Decline Reads Mapped to LE20-WE8 Genome Percentage** | | | | | |
| **Time** | **Control Abundance** | **H2O2 Abundance** | **T-Test Results (Comparing Treatments)** | **Significant**  **T-Test Results (Comparing H2O2)** | **Significant**  **T-Test Results (Comparing Control)** |
| 0hr | 0.10% ±  0.01% | 0.10% ±  0.01% | 1.000 | No significant results | No significant results |
| 30m | 0.10% ±  0.03% | 0.10% ±  0.05% | 1.000 |  |  |
| 2hr | 0.04% ±  0.003% | 0.03% ±  0.006% | 0.081 |  |  |
| 24hr | 0.10% ±  0.05% | 0.03% ±  0.01% | 0.081 |  |  |

**Table S6**. Average percentage of reads mapped to the *rpoB* gene in *Microcystis aeruginosa* NIES-843, expressed as the proportion of mapped reads relative to total sample reads. Values are reported as mean ± standard deviation (SD) from biological replicates. No outliers were detected using the Tukey IQR method. Unpaired t-tests were conducted to assess: (1) differences between timepoints within 10 µM hydrogen peroxide treated samples, (2) differences between timepoints within control samples, and (3) differences between control and 10 µM hydrogen peroxide treated samples at each timepoint. The same 0 hr values were used for both groups, resulting in a p-value of 1. The 8 hr timepoint was not collected in September.

| **Pre Bloom Reads Mapped to *Microcystis rpoB* Percentage** | | | | | |
| --- | --- | --- | --- | --- | --- |
| **Time** | **Control Abundance** | **H2O2 Abundance** | **T-Test Results (Comparing Treatments)** | **Significant**  **T-Test Results (Comparing H2O2)** | **Significant**  **T-Test Results (Comparing Control)** |
| 0hr | 0.005% ± 0.002% | 0.005% ± 0.002% | 1.000 | No significant results | No significant results |
| 30m | 0.005% ±  0.0002% | 0.004% ±  0.003% | 0.663 |  |  |
| 2hr | 0.004% ±  0.001% | 0.006% ±  0.0002% | 0.149 |  |  |
| 8hr | 0.003% ±  0.002% | 0.004% ±  0.0004% | 0.699 |  |  |
| 24hr | 0.003% ±  0.003 | 0.006% ±  0.0004% | 0.245 |  |  |
| **Peak Bloom Reads Mapped to *Microcystis rpoB* Percentage** | | | | | |
| **Time** | **Control Abundance** | **H2O2 Abundance** | **T-Test Results (Comparing Treatments)** | **Significant**  **T-Test Results (Comparing H2O2)** | **Significant**  **T-Test Results (Comparing Control)** |
| 0hr | 0.007% ±  0.003% | 0.007% ±  0.003% | 1.000 | No significant results | No significant results |
| 30m | 0.01% ±  0.007% | 0.004% ±  0.0007% | 0.190 |  |  |
| 2hr | 0.009% ±  0.001% | 0.002% ±  0.0002% | 0.081 |  |  |
| 8hr | 0.022% ±  0.014% | 0.0009% ±  0.0005% | 0.081 |  |  |
| 24hr | 0.012% ±  0.012% | 0.001% ±  0.0004% | 0.081 |  |  |
| **Bloom Decline Reads Mapped to *Microcystis rpoB* Percentage** | | | | | |
| **Time** | **Control Abundance** | **H2O2 Abundance** | **T-Test Results (Comparing Treatments)** | **Significant**  **T-Test Results (Comparing H2O2)** | **Significant**  **T-Test Results (Comparing Control)** |
| 0hr | 0.004% ±  0.0003% | 0.004% ±  0.0003% | 1.000 | No significant results | No significant results |
| 30m | 0.004 ±  0.0006% | 0.036% ±  0.001% | 1.000 |  |  |
| 2hr | 0.004% ±  0.0006% | 0.004% ±  0.0006% | 0.663 |  |  |
| 24hr | 0.004% ±  0.0003% | 0.004% ±  0.0003% | 0.663 |  |  |

**Table S7.** Average percentage of reads mapped to the *rpoB* gene in *Dolichospermum* LE20-WE8, expressed as the proportion of mapped reads relative to total sample reads. Values are reported as mean ± standard deviation (SD) from biological replicates. No outliers were detected using the Tukey IQR method. Unpaired t-tests were conducted to assess: (1) differences between timepoints within 10 µM hydrogen peroxide treated samples, (2) differences between timepoints within control samples, and (3) differences between control and 10 µM hydrogen peroxide treated samples at each timepoint. The same 0 hr values were used for both groups, resulting in a p-value of 1. The 8 hr timepoint was not collected in September.

| **Pre Bloom Reads Mapped to *Dolichospermum rpoB* Percentage** | | | | | |
| --- | --- | --- | --- | --- | --- |
| **Time** | **Control Abundance** | **H2O2 Abundance** | **T-Test Results (Comparing Treatments)** | **Significant**  **T-Test Results (Comparing H2O2)** | **Significant**  **T-Test Results (Comparing Control)** |
| 0hr | 0.002% ±  0.0005% | 0.002% ±  0.0005% | 1.000 | No significant results | No significant results |
| 30m | 0.006% ±  0.006% | 0.003% ±  0.0005% | 0.663 |  |  |
| 2hr | 0.006% ±  0.005% | 0.003% ±  0.0007% | 0.663 |  |  |
| 8hr | 0.002% ±  0.0007% | 0.002% ±  0.0007% | 0.081 |  |  |
| 24hr | 0.002% ±  0.0006 | 0.003% ±  0.0005% | 0.081 |  |  |
| **Peak Bloom Reads Mapped to *Dolichospermum rpoB* Percentage** | | | | | |
| **Time** | **Control Abundance** | **H2O2 Abundance** | **T-Test Results (Comparing Treatments)** | **Significant**  **T-Test Results (Comparing H2O2)** | **Significant**  **T-Test Results (Comparing Control)** |
| 0hr | 0.009% ±  0.009% | 0.009% ±  0.009% | 1.000 | No significant results | No significant results |
| 30m | 0.013% ±  0.012% | 0.007% ±  0.001% | 1.000 |  |  |
| 2hr | 0.012% ±  0.010% | 0.0003% ±  0.0003% | 0.773 |  |  |
| 8hr | 0.025% ±  0.009% | 0.001% ±  0.0008% | 0.699 |  |  |
| 24hr | 0.024% ±  0.017% | 0.0008% ±  0.0002% | 0.245 |  |  |
| **Post Bloom Reads Mapped to *Dolichospermum rpoB* Percentage** | | | | | |
| **Time** | **Control Abundance** | **H2O2 Abundance** | **T-Test Results (Comparing Treatments)** | **Significant**  **T-Test Results (Comparing H2O2)** | **Significant**  **T-Test Results (Comparing Control)** |
| 0hr | 0.002% ±  0.0001% | 0.002% ±  0.0001% | 1.000 | No significant results | No significant results |
| 30m | 0.002% ±  0.0002% | 0.002% ±  0.0004% | 1.000 |  |  |
| 2hr | 0.002% ±  0.0003% | 0.002% ±  0.0002% | 0.383 |  |  |
| 24hr | 0.002% ±  0.0001% | 0.002% ±  0.0002% | 1.000 |  |  |

**Table S8.** Mean relative abundance of Cyanobacteria phyla across timepoints during the three sampling periods. Values are reported as mean ± standard deviation (SD) based on biological replicates. Outliers were removed using the Tukey IQR method. Unpaired t-tests were conducted to assess: (1) differences between timepoints within 10 µM hydrogen peroxide-treated samples, (2) differences between timepoints within control samples, and (3) differences between hydrogen peroxide treated and control samples at each timepoint. Statistical significance is annotated with asterisks (* = *p* < 0.05, ** = *p* < 0.005, *** = *p* < 0.0005). For the 0 hr comparison, the same baseline values were used for both treatment and control groups, resulting in a p-value of 1. The 8 hr timepoint was not collected in September.

| **Pre Bloom Cyanobacteria Phylum Abundance** | | | | | |
| --- | --- | --- | --- | --- | --- |
| **Time** | **Control Abundance** | **H2O2 Abundance** | **T-Test Results (Comparing Treatments)** | **Significant**  **T-Test Results (Comparing H2O2)** | **Significant**  **T-Test Results (Comparing Control)** |
| 0hr | 0.0579 ±  0.0250 | 0.0579 ±  0.0250 | 1.000 | No significant comparisons | No significant comparisons |
| 30m | 0.3529 ±  0.4822 | 0.2564 ±  0.3757 | 0.799 |  |  |
| 2hr | 0.1563 ±  0.0355 | 0.0379 ±  0.0122 | 0.104 |  |  |
| 8hr | 0.1292 ±  0.0344 | 0.0329 ±  0.0023 | 0.156 |  |  |
| 24hr | 0.0743 ±  0.0425 | 0.0285 ±  0.0005 | 0.370 |  |  |
| **Peak Bloom Cyanobacteria Phylum Abundance** | | | | | |
| **Time** | **Control Abundance** | **H2O2 Abundance** | **T-Test Results (Comparing Treatments)** | **Significant**  **T-Test Results (Comparing H2O2)** | **Significant**  **T-Test Results (Comparing Control)** |
| 0hr | 0.3949 ±  0.4820 | 0.3949 ±  0.4820 | 1.000 | 30min > 2hr,  p = 0.0479  30min > 24hr,  p = 0.0035  2hr > 24hr,  p = 0.0258 | No significant comparisons |
| 30m | 0.9539 ±  0.0292 | 0.8824 ±  0.0120 | 0.150 |  |  |
| 2hr | 0.9604 ±  0.0186 | 0.8282 ±  0.0254 | 0.008 ** |  |  |
| 8hr | 0.9385 ±  0.0123 | 0.4985 ±  0.2011 | 0.063 |  |  |
| 24hr | 0.7128 ±  0.4155 | 0.0949 ±  0.0867 | 0.118 |  |  |
| **Bloom Decline Cyanobacteria Phylum Abundance** | | | | | |
| **Time** | **Control Abundance** | **H2O2 Abundance** | **T-Test Results (Comparing Treatments)** | **Significant**  **T-Test Results (Comparing H2O2)** | **Significant**  **T-Test Results (Comparing Control)** |
| 0hr | 0.0433 ±  0.0065 | 0.0433 ±  0.0065 | 1.000 | 2hr > 24hr,  p = 0.0072 | No significant comparisons |
| 30m | 0.0540 ±  0.0182 | 0.0313 ±  0.000 (n = 1) | N/A |  |  |
| 2hr | 0.0363 ±  0.0014 | 0.0360 ±  0.0006 | 0.800 |  |  |
| 24hr | 0.0358 ±  0.0026 | 0.0318 ±  0.0010 | 0.244 |  |  |

**Table S9.** Mean relative abundance of the *Microcystis* genus within the total microbial community across all timepoints during the three sampling trips. Values are reported as mean ± standard deviation (SD) from biological replicates. Outliers were removed using the Tukey IQR method. Unpaired t-tests were conducted to assess: (1) differences between timepoints within 10 µM hydrogen peroxide treated samples, (2) differences between timepoints within control samples, and (3) differences between hydrogen peroxide treated and control samples at each timepoint. Statistical significance is annotated with asterisks (* = *p* < 0.05, ** = *p* < 0.005, *** = *p* < 0.0005). For the 0 hr comparison, the same baseline values were used for both treatment and control, resulting in a p-value of 1. The 8 hr timepoint was not collected in September.

| **Pre Bloom *Microcystis* Abundance** | | | | | |
| --- | --- | --- | --- | --- | --- |
| **Time** | **Control Abundance** | **H2O2 Abundance** | **T-Test Results (Comparing Treatments)** | **Significant**  **T-Test Results (Comparing H2O2)** | **Significant**  **T-Test Results (Comparing Control)** |
| 0hr | 0.0115 ±  0.0032 | 0.0115 ±  0.0032 | 1.000 | No significant comparisons | No significant comparisons |
| 30m | 0.0365 ±  0.0410 | 0.0349 ±  0.0297 | 0.810 |  |  |
| 2hr | 0.0330 ±  0.0275 | 0.0160 ±  0.0015 | 0.0805 |  |  |
| 8hr | 0.0130 ±  0.0048 | 0.0096 ±  0.0002 | 0.0749 |  |  |
| 24hr | 0.0122 ±  0.0003 | 0.0130 ±  0.0003 | 0.330 |  |  |
| **Peak Bloom *Microcystis* Abundance** | | | | | |
| **Time** | **Control Abundance** | **H2O2 Abundance** | **T-Test Results (Comparing Treatments)** | **Significant**  **T-Test Results (Comparing H2O2)** | **Significant**  **T-Test Results (Comparing Control)** |
| 0hr | 0.0408 ±  0.0480 | 0.0408 ±  0.0480 | 1.000 | No significant comparisons | No significant comparisons |
| 30m | 0.0710 ±  0.0547 | 0.1980 ±  0.0141 | 0.0235 * |  |  |
| 2hr | 0.0828 ±  0.0276 | 0.1512 ±  0.0122 | 0.000175 *** |  |  |
| 8hr | 0.0967 ±  0.0259 | 0.0511 ±  0.0199 | 0.453 |  |  |
| 24hr | 0.0576 ±  0.0370 | 0.0125 ±  0.0127 | 0.255 |  |  |
| **Bloom Decline *Microcystis* Abundance** | | | | | |
| **Time** | **Control Abundance** | **H2O2 Abundance** | **T-Test Results (Comparing Treatments)** | **Significant**  **T-Test Results (Comparing H2O2)** | **Significant**  **T-Test Results (Comparing Control)** |
| 0hr | 0.0098 ±  0.0013 | 0.0098 ±  0.0013 | 1.000 | No significant comparisons | No significant comparisons |
| 30m | 0.0104 ±  0.0008 | 0.0140 ±  0.0066 | 1.000 |  |  |
| 2hr | 0.0098 ±  0.0010 | 0.0091 ±  0.0012 | 0.914 |  |  |
| 24hr | 0.0103 ±  0.0021 | 0.0080 ±  0.0010 | 0.309 |  |  |

**Table S10.** Mean relative abundance of the *Dolichospermum* genus within the total microbial community across all timepoints during the three sampling trips. Values are reported as mean ± standard deviation (SD) from biological replicates. Outliers were removed using the Tukey IQR method. Unpaired t-tests were conducted to assess: (1) differences between timepoints within 10 µM hydrogen peroxide treated samples, (2) differences between timepoints within control samples, and (3) differences between hydrogen peroxide treated and control samples at each timepoint. Statistical significance is annotated with asterisks (* = *p* < 0.05, ** = *p* < 0.005, *** = *p* < 0.0005). For the 0 hr comparison, the same baseline values were used for both treatment and control, resulting in a p-value of 1. The 8 hr timepoint was not collected in September.

| **Pre Bloom *Dolichospermum* Abundance** | | | | | |
| --- | --- | --- | --- | --- | --- |
| **Time** | **Control Abundance** | **H2O2 Abundance** | **T-Test Results (Comparing Treatments)** | **Significant**  **T-Test Results (Comparing H2O2)** | **Significant**  **T-Test Results (Comparing Control)** |
| 0hr | 0.0018 ±  0.0011 | 0.0018 ±  0.0011 | 1.000 | No significant comparisons | No significant comparisons |
| 30m | 0.0302 ±  0.0477 | 0.0048 ±  0.0068 | 1.000 |  |  |
| 2hr | 0.0239 ±  0.0333 | 0.0008 ±  3.33e-4 | 1.000 |  |  |
| 8hr | 0.0043 ±  6.67e-4 | 0.0005 ±  8.12e-5 | 0.141 |  |  |
| 24hr | 0.0023 ±  0.0022 | 0.0005 ±  1.44e-4 | 1.000 |  |  |
| **Peak Bloom *Dolichospermum* Abundance** | | | | | |
| **Time** | **Control Abundance** | **H2O2 Abundance** | **T-Test Results (Comparing Treatments)** | **Significant**  **T-Test Results (Comparing H2O2)** | **Significant**  **T-Test Results (Comparing Control)** |
| 0hr | 0.0577 ±  0.0910 | 0.0577 ±  0.0910 | 1.000 | No significant comparisons | No significant comparisons |
| 30m | 0.0786 ±  0.0866 | 0.0194 ±  0.0070 | 0.320 |  |  |
| 2hr | 0.0945 ±  0.0836 | 0.0090 ±  0.0019 | 0.225 |  |  |
| 8hr | 0.0809 ±  0.0344 | 0.0032 ±  2.37e-4 | 0.016* |  |  |
| 24hr | 0.1069 ±  0.0820 | 0.0015 ±  0.0023 | 0.113 |  |  |
| **Bloom Decline *Dolichospermum* Abundance** | | | | | |
| **Time** | **Control Abundance** | **H2O2 Abundance** | **T-Test Results (Comparing Treatments)** | **Significant**  **T-Test Results (Comparing H2O2)** | **Significant**  **T-Test Results (Comparing Control)** |
| 0hr | 0.0006 ±  7.80e-5 | 0.0006 ±  7.80e-5 | 1.000 | No significant comparisons | No significant comparisons |
| 30m | 0.0008 ±  3.15e-4 | 0.0007 ±  4.65e-4 | 1.000 |  |  |
| 2hr | 0.0005 ±  3.12e-5 | 0.0003 ±  5.71e-5 | 0.207 |  |  |
| 24hr | 0.0007 ±  4.57e-4 | 0.0003 ±  6.50e-5 | 1.000 |  |  |

**Table S11.** Catalase marker gene expression across June, August, and September sampling seasons, reported as the mean ± standard deviation of the catalase marker gene:*rpoB* ratio. Unpaired *t*-tests were used to assess statistical differences across three comparison types: between treatments at each timepoint (hydrogen peroxide vs. control), within the hydrogen peroxide treatment time series, and within the control time series. Significance thresholds are indicated as follows: *p* < 0.05 = *, *p* < 0.005 = **, *p* < 0.0005 = ***. Note: the September time series does not include an 8-hour timepoint. Significant differences within the hydrogen peroxide treatment group marked with “(R)” were excluded from interpretation, as they were also observed in the control group and likely reflect time-of-day effects rather than treatment-specific responses.

| **Pre Bloom Catalase Marker Genes:*rpoB* Expression** | | | | | | |
| --- | --- | --- | --- | --- | --- | --- |
| **Genus** | **Time** | **Control Ratio** | **H2O2 Ratio** | **T-Test Results (Comparing Treatments)** | **Significant**  **T-Test Results (Comparing H2O2)** | **Significant**  **T-Test Results (Comparing Control)** |
| *Aquirufa* | 0hr | 0.649 ± 0.090 | 0.649 ± 0.090 | 1.000 | 8hr > 2hr,  p = 0.028 | No significant comparisons |
|  | 30m | 0.444 ±  0.308 | 0.641 ± 0.263 | 0.446 |  |  |
|  | 2hr | 0.389 ±  0.267 | 0.708 ± 0.022 | 0.208 |  |  |
|  | 8hr | 0.489 ±  0.150 | 0.617 ± 0.004 | 0.349 |  |  |
|  | 24hr | 0.503 ±  0.232 | 0.648 ± 0.042 | 0.474 |  |  |
| *Chitinilyticum* | 0hr | 0.287 ± 0.020 | 0.287 ± 0.020 | 1.000 | 8hr > 0hr,  p = 0.032 (R) | 8hr > 0hr,  p = 0.021 |
|  | 30m | 0.251 ±  0.201 | 0.760 ± 0.632 | 0.255 |  |  |
|  | 2hr | 0.304 ±  0.078 | 0.349 ± 0.016 | 0.495 |  |  |
|  | 8hr | 0.391 ±  0.008 | 0.377 ± 0.012 | 0.297 |  |  |
|  | 24hr | 0.569 ±  0.315 | 0.288 ± 0.041 | 0.338 |  |  |
| *Chitinimonas* | 0hr | 0.123 ± 0.014 | 0.123 ± 0.014 | 1.000 | No significant comparisons | No significant comparisons |
|  | 30m | 0.077 ±  0.067 | 0.124 ± 0.058 | 0.409 |  |  |
|  | 2hr | 0.071 ±  0.062 | 0.098 ± 0.002 | 0.607 |  |  |
|  | 8hr | 0.108 ±  0.009 | 0.116 ± 0.009 | 0.491 |  |  |
|  | 24hr | 0.106 ±  0.035 | 0.110 ± 0.016 | 0.898 |  |  |
| *Cupriavidus* | 0hr | 0.884 ± 0.120 | 0.884 ± 0.120 | 1.000 | No significant comparisons | No significant comparisons |
|  | 30m | 0.600 ±  0.528 | 0.943 ± 0.199 | 0.351 |  |  |
|  | 2hr | 0.524 ±  0.282 | 0.826 ± 0.052 | 0.249 |  |  |
|  | 8hr | 0.931 ±  0.189 | 0.739 ± 0.004 | 0.287 |  |  |
|  | 24hr | 0.774 ±  0.131 | 0.827 ± 0.046 | 0.647 |  |  |
| *Defluviimonas* | 0hr | 0.369 ± 0.016 | 0.369 ± 0.016 | 1.000 | No significant comparisons | 8hr > 0hr,  p = 0.024 |
|  | 30m | 0.348 ±  0.087 | 0.828 ± 0.673 | 0.288 |  |  |
|  | 2hr | 0.454 ±  0.087 | 0.432 ± 0.015 | 0.760 |  |  |
|  | 8hr | 0.451 ±  0.008 | 0.436 ± 0.018 | 0.382 |  |  |
|  | 24hr | 0.648 ±  0.284 | 0.413 ± 0.056 | 0.369 |  |  |
| *Deinococcus* | 0hr | 1.087 ± 0.071 | 1.087 ± 0.071 | 1.000 | No significant comparisons | No significant comparisons |
|  | 30m | 0.730 ±  0.633 | 0.722 ± 0.520 | 0.988 |  |  |
|  | 2hr | 0.636 ±  0.546 | 1.020 ± 0.011 | 0.415 |  |  |
|  | 8hr | 0.886 ±  0.135 | 1.062 ± 0.054 | 0.229 |  |  |
|  | 24hr | 0.852 ±  0.337 | 1.070 ± 0.028 | 0.459 |  |  |
| *Enterobacter* | 0hr | 1.361 ± 0.089 | 1.361 ± 0.089 | 1.000 | 0hr > 2hr,  p = 0.015  8hr > 2hr,  p = 0.044 | No significant comparisons |
|  | 30m | 0.956 ±  0.779 | 0.676 ± 0.340 | 0.599 |  |  |
|  | 2hr | 0.835 ±  0.595 | 0.855 ± 0.007 | 0.968 |  |  |
|  | 8hr | 1.038 ±  0.566 | 1.409 ± 0.170 | 0.469 |  |  |
|  | 24hr | 1.139 ±  0.557 | 1.249 ± 0.342 | 0.834 |  |  |
| *Escherichia* | 0hr | 6.659 ± 0.641 | 6.659 ± 0.641 | 1.000 | 8hr > 24hr,  p = 0.031 | No significant comparisons |
|  | 30m | 4.393 ±  3.748 | 4.804 ± 3.549 | 0.897 |  |  |
|  | 2hr | 3.235 ±  2.809 | 6.680 ± 0.359 | 0.200 |  |  |
|  | 8hr | 4.356 ±  2.097 | 6.482 ± 0.076 | 0.288 |  |  |
|  | 24hr | 3.516 ±  4.244 | 7.221 ± 0.171 | 0.343 |  |  |
| *Exiguobacterium* | 0hr | 0.752 ± 0.040 | 0.752 ± 0.040 | 1.000 | No significant comparisons | No significant comparisons |
|  | 30m | 0.493 ±  0.428 | 0.519 ± 0.432 | 0.945 |  |  |
|  | 2hr | 0.390 ±  0.339 | 0.782 ± 0.016 | 0.218 |  |  |
|  | 8hr | 0.550 ±  0.157 | 0.731 ± 0.119 | 0.322 |  |  |
|  | 24hr | 0.625 ±  0.231 | 0.804 ± 0.026 | 0.392 |  |  |
| *Formosimonas* | 0hr | 0.594 ± 0.019 | 0.594 ± 0.019 | 1.000 | 24hr > 8hr,  p = 0.023 | No significant comparisons |
|  | 30m | 0.439 ±  0.275 | 0.661 ± 0.063 | 0.243 |  |  |
|  | 2hr | 0.420 ±  0.215 | 0.565 ± 0.044 | 0.436 |  |  |
|  | 8hr | 0.523 ±  0.086 | 0.573 ± 0.001 | 0.498 |  |  |
|  | 24hr | 0.821 ±  0.309 | 0.628 ± 0.012 | 0.472 |  |  |
| *Limnobacter* | 0hr | 0.401 ± 0.037 | 0.401 ± 0.037 | 1.000 | No significant comparisons | No significant comparisons |
|  | 30m | 0.319 ±  0.119 | 0.642 ± 0.472 | 0.314 |  |  |
|  | 2hr | 0.355 ±  0.068 | 0.394 ± 0.017 | 0.502 |  |  |
|  | 8hr | 0.399 | 0.392 ± 0.031 | 0.775 |  |  |
|  | 24hr | 0.452 ±  0.018 | 0.388 ± 0.028 | 0.113 |  |  |
| *Paenibacillus* | 0hr | 3.290 ± 0.027 | 3.290 ± 0.027 | 1.000 | 0hr > 24hr,  p = 0.042  24hr > 2hr,  p = 0.017 | No significant comparisons |
|  | 30m | 2.252 ±  1.940 | 2.371 ± 1.710 | 0.940 |  |  |
|  | 2hr | 1.961 ±  1.671 | 3.326 ± 0.013 | 0.353 |  |  |
|  | 8hr | 2.896 ±  0.235 | 3.184 ± 0.052 | 0.232 |  |  |
|  | 24hr | 2.416 ±  1.200 | 3.159 ± 0.028 | 0.474 |  |  |
| *Phenylobacterium* | 0hr | 0.832 ± 0.022 | 0.832 ± 0.022 | 1.000 | No significant comparisons | No significant comparisons |
|  | 30m | 0.593 ±  0.361 | 1.100 ± 0.454 | 0.205 |  |  |
|  | 2hr | 0.610 ±  0.266 | 0.846 ± 0.001 | 0.319 |  |  |
|  | 8hr | 0.765 ±  0.062 | 0.799 ± 0.018 | 0.525 |  |  |
|  | 24hr | 0.883 ±  0.104 | 0.828 ± 0.034 | 0.548 |  |  |
| *Polynucleobacter* | 0hr | 0.283 ± 0.004 | 0.283 ± 0.004 | 1.000 | 0hr > 2hr,  p = 0.003  8hr > 2hr,  p = 0.026 | No significant comparisons |
|  | 30m | 0.207 ±  0.117 | 0.220 ± 0.118 | 0.898 |  |  |
|  | 2hr | 0.183 ±  0.104 | 0.172 ± 0.008 | 0.899 |  |  |
|  | 8hr | 0.209 ±  0.099 | 0.284 ± 0.025 | 0.409 |  |  |
|  | 24hr | 0.230 ±  0.077 | 0.241 ± 0.066 | 0.890 |  |  |
| *Pseudomonas* | 0hr | 0.314 ± 0.038 | 0.314 ± 0.038 | 1.000 | 2hr > 8hr,  p = 0.022 | No significant comparisons |
|  | 30m | 0.267 ±  0.191 | 0.661 ± 0.752 | 0.429 |  |  |
|  | 2hr | 0.238 ±  0.046 | 0.259 ± 0.021 | 0.593 |  |  |
|  | 8hr | 0.225 ±  0.123 | 0.359 ± 0.001 | 0.265 |  |  |
|  | 24hr | 0.262 ±  0.194 | 0.354 ± 0.064 | 0.591 |  |  |
| *Pseudotabrizicola* | 0hr | 0.471 ± 0.041 | 0.471 ± 0.041 | 1.000 | No significant comparisons | No significant comparisons |
|  | 30m | 0.374 ±  0.208 | 0.643 ± 0.295 | 0.265 |  |  |
|  | 2hr | 0.358 ±  0.182 | 0.478 ± 0.013 | 0.443 |  |  |
|  | 8hr | 0.486 ±  0.003 | 0.472 ± 0.009 | 0.174 |  |  |
|  | 24hr | 0.535 ±  0.074 | 0.439 ± 0.043 | 0.253 |  |  |
| *Puniceibacterium* | 0hr | 0.227 ± 0.011 | 0.227 ± 0.011 | 1.000 | 24hr > 2hr,  p = 0.042  0hr > 2hr,  p = 0.038 (R) | 0hr > 2hr,  p = 0.009 |
|  | 30m | 0.180 ±  0.078 | 0.485 ± 0.531 | 0.380 |  |  |
|  | 2hr | 0.170 ±  0..010 | 0.172 ± 0.011 | 0.808 |  |  |
|  | 8hr | 0.211 ±  0.038 | 0.189 ± 0.015 | 0.541 |  |  |
|  | 24hr | 0.371 ±  0.253 | 0.246 ± 0.019 | 0.559 |  |  |
| *Rhizobium* | 0hr | 1.744 ± 0.049 | 1.744 ± 0.049 | 1.000 | No significant comparisons | No significant comparisons |
|  | 30m | 1.184 ±  0.844 | 1.710 ± 0.271 | 0.363 |  |  |
|  | 2hr | 1.142 ±  0.695 | 1.841 ± 0.127 | 0.273 |  |  |
|  | 8hr | 1.450 ±  0.190 | 1.796 ± 0.014 | 0.125 |  |  |
|  | 24hr | 1.443 ±  0.449 | 1.761 ± 0.108 | 0.432 |  |  |
| *Rhodococcus* | 0hr | 1.095 ± 0.038 | 1.095 ± 0.038 | 1.000 | 24hr > 2hr,  p = 0.011 | No significant comparisons |
|  | 30m | 0.720 ±  0.624 | 0.773 ± 0.660 | 0.924 |  |  |
|  | 2hr | 0.646 ±  0.547 | 1.169 ± 0.006 | 0.290 |  |  |
|  | 8hr | 0.813 ±  0.245 | 1.091 ± 0.041 | 0.255 |  |  |
|  | 24hr | 0.666 ±  0.517 | 1.091 ± 0.010 | 0.366 |  |  |
| *Sphingobium* | 0hr | 1.584 ± 0.018 | 1.584 ± 0.018 | 1.000 | 24hr > 2hr,  p = 0.039 | No significant comparisons |
|  | 30m | 1.012 ±  0.847 | 1.467 ± 0.122 | 0.409 |  |  |
|  | 2hr | 0.972 ±  0.728 | 1.524 ± 0.010 | 0.384 |  |  |
|  | 8hr | 1.428 ±  0.143 | 1.581 ± 0.026 | 0.275 |  |  |
|  | 24hr | 1.354 ±  0.326 | 1.693 ± 0.048 | 0.283 |  |  |
| *Stenotrophomonas* | 0hr | 3.167 ± 0.088 | 3.167 ± 0.088 | 1.000 | No significant comparisons | No significant comparisons |
|  | 30m | 2.091 ±  1.743 | 2.731 ± 0.729 | 0.589 |  |  |
|  | 2hr | 1.816 ±  1.275 | 2.993 ± 0.048 | 0.304 |  |  |
|  | 8hr | 2.730 ±  0.707 | 3.435 ± 0.199 | 0.307 |  |  |
|  | 24hr | 2.328 ±  1.400 | 3.062 ± 0.088 | 0.536 |  |  |
| *Streptomyces* | 0hr | 3.382 ± 0.102 | 3.382 ± 0.102 | 1.000 | 2hr > 8hr,  p = 0.029 | No significant comparisons |
|  | 30m | 2.291 ±  1.875 | 3.117 ± 1.515 | 0.585 |  |  |
|  | 2hr | 2.189 ±  1.636 | 3.775 ± 0.086 | 0.284 |  |  |
|  | 8hr | 3.124 ±  0.402 | 3.427 ± 0.009 | 0.399 |  |  |
|  | 24hr | 2.813 ±  1.084 | 3.567 ± 0.156 | 0.433 |  |  |
| *Szabonella* | 0hr | 0.155 ± 0.016 | 0.155 ± 0.016 | 1.000 | No significant comparisons | No significant comparisons |
|  | 30m | 0.120 ±  0.104 | 0.149 ± 0.049 | 0.687 |  |  |
|  | 2hr | 0.122 ±  0.106 | 0.174 ± 0.004 | 0.563 |  |  |
|  | 8hr | 0.179 ±  0.008 | 0.176 ± 0.002 | 0.595 |  |  |
|  | 24hr | 0.145 ±  0.045 | 0.152 ± 0.020 | 0.855 |  |  |
| **Peak Bloom Catalase Marker Gene:*rpoB* Expression** | | | | | | |
| **Genus** | **Time** | **Control Ratio** | **H2O2 Ratio** | **T-Test Results (Comparing Treatments)** | **Significant**  **T-Test Results (Comparing H2O2)** | **Significant**  **T-Test Results (Comparing Control)** |
| *Aquirufa* | 0hr | 0.494 ±  0.416 | 0.494 ± 0.416 | 1.000 | No significant comparisons | No significant comparisons |
|  | 30m | 0.393 ±  0.369 | 1.031 ± 1.048 | 0.335 |  |  |
|  | 2hr | 0.242 ±  0.204 | 2.431 ± 1.804 | 0.105 |  |  |
|  | 8hr | 0.033 ±  0.027 | 0.517 ± 0.321 | 0.060 |  |  |
|  | 24hr | 0.268 ±  0.425 | 0.562 ± 0.082 | 0.304 |  |  |
| *Chitinilyticum* | 0hr | 0.377 ± 0.101 | 0.377 ± 0.101 | 1.000 | 24hr > 0hr,  p = 0.000868  30min > 0hr,  p = 0.002  24hr > 30m,  p = 0.034  8hr > 30min,  p = 0.035  8hr > 0hr,  p = 0.019 (R) | 8hr > 0hr,  p = 0.026 |
|  | 30m | 0.581 ±  0.320 | 0.957 ± 0.105 | 0.126 |  |  |
|  | 2hr | 0.946 ±  0.962 | 2.358 ± 1.776 | 0.293 |  |  |
|  | 8hr | 0.168 ±  0.028 | 3.593 ± 1.459 | 0.153 |  |  |
|  | 24hr | 0.504 ±  0.329 | 1.283 ± 0.144 | 0.020 * |  |  |
| *Chitinimonas* | 0hr | 0.055 ± 0.024 | 0.055 ± 0.024 | 1.000 | 24hr > 0hr,  p = 0.017  0hr > 2hr,  p = 0.016  24hr > 2hr,  p = 0.006  24hr > 30min,  p = 0.008 | No significant comparisons |
|  | 30m | 0.024 ±  0.039 | 0.013 ± 0.022 | 0.689 |  |  |
|  | 2hr | 0.013 ±  0.022 | 0.000 | 0.374 |  |  |
|  | 8hr | 0.252 ±  0.281 | 0.252 ± 0.281 | 0.218 |  |  |
|  | 24hr | 0.019 ±  0.033 | 0.237 ± 0.0759 | 0.010 * |  |  |
| *Cupriavidus* | 0hr | 0.863 ± 0.455 | 0.863 ± 0.455 | 1.000 | 8hr > 0hr,  p = 0.022  24hr > 30min,  p = 0.004  8hr > 30min,  p = 0.012 | No significant comparisons |
|  | 30m | 0.474 ±  0.325 | 0.567 ± 0.260 | 0.718 |  |  |
|  | 2hr | 1.765 ±  2.405 | 3.176 ± 1.675 | 0.451 |  |  |
|  | 8hr | 0.200 ±  0.083 | 3.146 ± 0.995 | 0.007 * |  |  |
|  | 24hr | 0.611 ±  0.449 | 1.556 ± 0.135 | 0.025 * |  |  |
| *Defluviimonas* | 0hr | 0.319 ± 0.207 | 0.319 ± 0.207 | 1.000 | 24hr > 0hr,  p = 0.000964  8hr > 0hr,  p = 0.023  24hr > 30min,  p = 0.016 | No significant comparisons |
|  | 30m | 0.542 ±  0.431 | 0.637 ± 0.354 | 0.782 |  |  |
|  | 2hr | 0.714 ±  0.576 | 4.069 ± 4.219 | 0.244 |  |  |
|  | 8hr | 0.192 ±  0.036 | 1.940 ± 0.752 | 0.016 * |  |  |
|  | 24hr | 0.307 ±  0.137 | 1.496 ± 0.110 | 0.0003 *** |  |  |
| *Deinococcus* | 0hr | 0.755 ± 0.640 | 0.755 ± 0.640 | 1.000 | 24hr > 2hr,  p = 0.001  24hr > 30min,  p = 0.00395 | No significant comparisons |
|  | 30m | 0.460 ±  0.772 | 0.038 ± 0.053 | 0.398 |  |  |
|  | 2hr | 0.025 ±  0.032 | 0.119 ± 0.054 | 0.058 |  |  |
|  | 8hr | 0.009 ±  0.013 | 0.391 ± 0.314 | 0.104 |  |  |
|  | 24hr | 0.356 ±  0.614 | 0.425 ± 0.031 | 0.856 |  |  |
| *Enterobacter* | 0hr | 0.493 ± 0.367 | 0.493 ± 0.367 | 1.000 | No significant comparisons | No significant comparisons |
|  | 30m | 0.533 ±  0.702 | 0.926 ± 0.906 | 0.584 |  |  |
|  | 2hr | 0.809 ±  0.772 | 1.577 ± 0.766 | 0.288 |  |  |
|  | 8hr | 0.084 ±  0.047 | 3.023 ± 1.894 | 0.055 |  |  |
|  | 24hr | 0.341 ±  0.295 | 0.840 ± 0.263 | 0.094 |  |  |
| *Escherichia* | 0hr | 4.528 ± 3.834 | 4.528 ± 3.834 | 1.000 | 30min > 24hr,  p = 0.014 | No significant comparisons |
|  | 30m | 2.257 ±  3.869 | 0.761 ± 0.132 | 0.540 |  |  |
|  | 2hr | 0.453 ±  0.434 | 2.337 ± 2.341 | 0.242 |  |  |
|  | 8hr | 0.061 ±  0.037 | 2.272 ± 1.732 | 0.092 |  |  |
|  | 24hr | 2.386 ±  3.762 | 0.363 ± 0.099 | 0.405 |  |  |
| *Exiguobacterium* | 0hr | 0.449 ± 0.383 | 0.449 ± 0.383 | 1.000 | 24hr > 2hr,  p = 0.008  24hr > 30min,  p = 0.009 | No significant comparisons |
|  | 30m | 0.232 ±  0.401 | 0.062 ± 0.045 | 0.507 |  |  |
|  | 2hr | 0.037 ±  0.063 | 0.053 ± 0.048 | 0.741 |  |  |
|  | 8hr | 0.000 | 0.883 ± 1.102 | 0.205 |  |  |
|  | 24hr | 0.188 ±  0.326 | 0.410 ± 0.118 | 0.330 |  |  |
| *Formosimonas* | 0hr | 0.450 ± 0.281 | 0.450 ± 0.281 | 1.000 | No significant comparisons | No significant comparisons |
|  | 30m | 0.388 ±  0.250 | 1.154 ± 1.145 | 0.321 |  |  |
|  | 2hr | 1.018 ±  0.762 | 47.98 ± 46.52 | 0.155 |  |  |
|  | 8hr | 0.218 ±  0.071 | 4.070 ± 3.613 | 0.136 |  |  |
|  | 24hr | 1.154 ±  1.406 | 1.213 ± 0.389 | 0.948 |  |  |
| *Limnobacter* | 0hr | 0.297 ± 0.156 | 0.297 ± 0.156 | 1.000 | 2hr > 0hr,  p = 0.040 | No significant comparisons |
|  | 30m | 0.319 ±  0.188 | 1.535 ± 1.828 | 0.316 |  |  |
|  | 2hr | 0.645 ±  0.556 | 2.895 ± 1.490 | 0.070 |  |  |
|  | 8hr | 0.095 ±  0.026 | 37.83 ± 63.15 | 0.359 |  |  |
|  | 24hr | 0.154 ±  0.188 | 0.552 ± 0.151 | 0.046 * |  |  |
| *Paenibacillus* | 0hr | 2.327 ± 1.977 | 2.327 ± 1.977 | 1.000 | No significant comparisons | No significant comparisons |
|  | 30m | 1.366 ±  2.320 | 0.707 ± 0.509 | 0.656 |  |  |
|  | 2hr | 0.262 ±  0.172 | 0.843 ± 0.815 | 0.293 |  |  |
|  | 8hr | 0.055 ±  0.032 | 0.880 ± 0.372 | 0.019 * |  |  |
|  | 24hr | 1.102 ±  1.825 | 0.766 ± 0.305 | 0.769 |  |  |
| *Phenylobacterium* | 0hr | 0.762 ± 0.407 | 0.762 ± 0.407 | 1.000 | 24hr > 0hr,  p = 0.015  8hr > 0hr,  p = 0.011  8hr > 30min,  p = 0.022 | 0hr > 8hr,  p = 0.046  30min > 8hr,  p = 0.036 |
|  | 30m | 0.755 ±  0.345 | 1.169 ± 0.384 | 0.341 |  |  |
|  | 2hr | 0.923 ±  0.705 | 14.92 ± 22.44 | 0.004 ** |  |  |
|  | 8hr | 0.133 ±  0.042 | 2.699 ± 0.616 | 0.002 ** |  |  |
|  | 24hr | 0.494 ±  0.336 | 1.789 ± 0.162 | 0.833 |  |  |
| *Polynucleobacter* | 0hr | 0.108 ± 0.052 | 0.108 ± 0.052 | 1.000 | 24hr > 0hr,  p = 0.000366  30min > 0hr,  p = 0.005  30min > 24hr,  p = 0.020 | No significant comparisons |
|  | 30m | 0.127 ±  0.124 | 1.301 ± 0.364 | 0.240 |  |  |
|  | 2hr | 0.348 ±  0.443 | 1.802 ± 1.770 | 8.34e-5 *** |  |  |
|  | 8hr | 0.081 ±  0.054 | 1.256 ± 0.967 | 0.103 |  |  |
|  | 24hr | 0.099 ±  0.027 | 0.505 ± 0.033 | 0.970 |  |  |
| *Pseudomonas* | 0hr | 0.218 ± 0.091 | 0.218 ± 0.091 | 1.000 | 2hr > 0hr,  p = 0.018  2hr > 24hr,  p = 0.027 | No significant comparisons |
|  | 30m | 0.395 ±  0.194 | 0.991 ± 0.507 | 0.130 |  |  |
|  | 2hr | 0.880 ±  0.612 | 3.151 ± 1.313 | 0.053 |  |  |
|  | 8hr | 0.146 ±  0.045 | 1.814 ± 1.369 | 0.103 |  |  |
|  | 24hr | 0.242 ±  0.053 | 0.541 ± 0.194 | 0.062 |  |  |
| *Pseudotabrizicola* | 0hr | 0.594 ± 0.356 | 0.594 ± 0.356 | 1.000 | 24hr > 0hr,  p = 0.000719  2hr > 0hr,  p = 0.046  8hr > 0hr,  p = 0.004  24hr > 30min,  p = 0.000999  2hr > 30min,  p = 0.049  8hr > 30min,  p = 0.010 | No significant comparisons |
|  | 30m | 0.516 ±  0.363 | 0.524 ± 0.481 | 0.982 |  |  |
|  | 2hr | 0.623 ±  0.507 | 1.935 ± 0.730 | 0.063 |  |  |
|  | 8hr | 0.067 ±  0.040 | 1.829 ± 0.095 | 7.68e-6 *** |  |  |
|  | 24hr | 0.293 ±  0.391 | 2.659 ± 0.137 | 5.86e-4 *** |  |  |
| *Puniceibacterium* | 0hr | 0.303 ± 0.191 | 0.303 ± 0.191 | 1.000 | 24hr > 0hr,  p = 0.003  2hr > 0hr,  p = 0.024  2hr > 30min,  p = 0.048 | No significant comparisons |
|  | 30m | 0.346 ±  0.240 | 0.672 ± 0.381 | 0.278 |  |  |
|  | 2hr | 0.679 ±  0.505 | 2.503 ± 1.062 | 0.055 |  |  |
|  | 8hr | 0.110 ±  0.038 | 2.362 ± 1.433 | 0.053 |  |  |
|  | 24hr | 0.178 ±  0.057 | 1.232 ± 0.154 | 3.69e-4 *** |  |  |
| *Rhizobium* | 0hr | 1.494 ± 1.136 | 1.494 ± 1.136 | 1.000 | No significant comparisons | No significant comparisons |
|  | 30m | 1.012 ±  0.929 | 1.167 ± 0.606 | 0.820 |  |  |
|  | 2hr | 1.050 ±  0.798 | 5.287 ± 6.076 | 0.297 |  |  |
|  | 8hr | 0.182 ±  0.044 | 4.257 ± 3.511 | 0.115 |  |  |
|  | 24hr | 0.889 ±  1.043 | 1.772 ± 0.301 | 0.232 |  |  |
| *Rhodococcus* | 0hr | 0.912 ± 0.755 | 0.912 ± 0.755 | 1.000 | No significant comparisons | No significant comparisons |
|  | 30m | 0.618 ±  0.968 | 0.045 ± 0.079 | 0.365 |  |  |
|  | 2hr | 0.019 ±  0.024 | 0.263 ± 0.435 | 0.387 |  |  |
|  | 8hr | 0.022 ±  0.034 | 0.292 ± 0.335 | 0.237 |  |  |
|  | 24hr | 0.424 ±  0.733 | 0.355 ± 0.214 | 0.882 |  |  |
| *Sphingobium* | 0hr | 1.092 ± 0.898 | 1.092 ± 0.898 | 1.000 | 24hr > 30min,  p = 0.000406 | No significant comparisons |
|  | 30m | 0.721 ±  0.844 | 0.462 ± 0.141 | 0.627 |  |  |
|  | 2hr | 0.590 ±  0.500 | 3.321 ± 3.531 | 0.255 |  |  |
|  | 8hr | 0.112 ±  0.034 | 3.465 ± 2.800 | 0.107 |  |  |
|  | 24hr | 0.553 ±  0.839 | 1.954 ± 0.191 | 0.045 * |  |  |
| *Stenotrophomonas* | 0hr | 1.911 ± 1.396 | 1.911 ± 1.396 | 1.000 | 8hr > 24hr,  p = 0.048  8hr > 30min,  p = 0.041 | No significant comparisons |
|  | 30m | 1.192 ±  1.413 | 1.154 ± 0.708 | 0.969 |  |  |
|  | 2hr | 1.029 ±  0.940 | 2.169 ± 1.207 | 0.266 |  |  |
|  | 8hr | 0.179 ±  0.051 | 3.612 ± 1.248 | 0.009 * |  |  |
|  | 24hr | 1.147 ±  1.402 | 1.574 ± 0.130 | 0.627 |  |  |
| *Streptomyces* | 0hr | 2.646 ± 2.157 | 2.646 ± 2.157 | 1.000 | 24hr > 30min,  p = 0.003 | No significant comparisons |
|  | 30m | 1.538 ±  1.799 | 1.198 ± 0.298 | 0.763 |  |  |
|  | 2hr | 1.079 ±  0.903 | 2.912 ± 1.861 | 0.200 |  |  |
|  | 8hr | 0.163 ±  0.076 | 3.783 ± 3.339 | 0.139 |  |  |
|  | 24hr | 1.599 ±  1.978 | 2.945 ± 0.357 | 0.310 |  |  |
| *Szabonella* | 0hr | 0.168 ± 0.123 | 0.168 ± 0.123 | 1.000 | No significant comparisons | No significant comparisons |
|  | 30m | 0.096 ±  0.109 | 0.000 | 0.204 |  |  |
|  | 2hr | 0.001 ±  0.002 | 0.000 | 0.374 |  |  |
|  | 8hr | 0.000 | 0.045 ± 0.066 | 0.303 |  |  |
|  | 24hr | 0.069 ±  0.120 | 0.095 ± 0.070 | 0.767 |  |  |
| **Bloom Decline Catalase Marker Genes:*rpoB* Expression** | | | | | | |
| **Genus** | **Time** | **Control Ratio** | **H2O2 Ratio** | **T-Test Results (Comparing Treatments)** | **Significant**  **T-Test Results (Comparing H2O2)** | **Significant**  **T-Test Results (Comparing Control)** |
| *Aquirufa* | 0hr | 0.766 ± 0.087 | 0.766 ± 0.087 | 1.000 | No significant comparisons | No significant comparisons |
|  | 30m | 0.764 ±  0.092 | 0.754 ± 0.040 | 0.894 |  |  |
|  | 2hr | 0.753 ±  0.040 | 0.734 ± 0.007 | 0.455 |  |  |
|  | 24hr | 0.716 ±  0.041 | 0.769  ± 0.039 | 0.184 |  |  |
| *Chitinilyticum* | 0hr | 0.435 ± 0.054 | 0.435 ± 0.054 | 1.000 | No significant comparisons | No significant comparisons |
|  | 30m | 0.467 ±  0.043 | 0.486 ± 0.083 | 0.743 |  |  |
|  | 2hr | 0.472 ±  0.035 | 0.450 ± 0.040 | 0.497 |  |  |
|  | 24hr | 0.466 ±  0.030 | 0.451 ± 0.007 | 0.449 |  |  |
| *Chitinimonas* | 0hr | 0.068 ± 0.005 | 0.068 ± 0.005 | 1.000 | No significant comparisons | No significant comparisons |
|  | 30m | 0.068 ±  0.010 | 0.071 ± 0.001 | 0.756 |  |  |
|  | 2hr | 0.070 ±  0.007 | 0.073 ± 0.002 | 0.555 |  |  |
|  | 24hr | 0.070 ±  0.006 | 0.068 ± 0.003 | 0.645 |  |  |
| *Cupriavidus* | 0hr | 0.994 ± 0.044 | 0.994 ± 0.044 | 1.000 | No significant comparisons | No significant comparisons |
|  | 30m | 1.158 ±  0.065 | 1.114 ± 0.182 | 0.711 |  |  |
|  | 2hr | 0.985 ±  0.106 | 1.011 ± 0.035 | 0.713 |  |  |
|  | 24hr | 0.995 ±  0.133 | 0.921 ± 0.093 | 0.472 |  |  |
| *Defluviimonas* | 0hr | 0.427 ± 0.017 | 0.427 ± 0.017 | 1.000 | No significant comparisons | No significant comparisons |
|  | 30m | 0.436 ±  0.015 | 0.440 ± 0.068 | 0.932 |  |  |
|  | 2hr | 0.428 ±  0.011 | 0.436 ± 0.027 | 0.662 |  |  |
|  | 24hr | 0.445 ±  0.018 | 0.439 ± 0.044 | 0.836 |  |  |
| *Deinococcus* | 0hr | 1.154 ± 0.065 | 1.154 ± 0.065 | 1.000 | No significant comparisons | No significant comparisons |
|  | 30m | 1.042 ±  0.088 | 1.075 ± 0.227 | 0.825 |  |  |
|  | 2hr | 1.193 ±  0.038 | 1.243 ± 0.059 | 0.287 |  |  |
|  | 24hr | 1.162 ±  0.101 | 1.283 ± 0.059 | 0.211 |  |  |
| *Enterobacter* | 0hr | 0.713 ± 0.053 | 0.713 ± 0.053 | 1.000 | No significant comparisons | No significant comparisons |
|  | 30m | 0.714 ±  0.020 | 0.721 ± 0.196 | 0.950 |  |  |
|  | 2hr | 0.819 ±  0.065 | 0.766 ± 0.090 | 0.453 |  |  |
|  | 24hr | 0.983 ±  0.428 | 0.894 ± 0.075 | 0.740 |  |  |
| *Escherichia* | 0hr | 6.728 ± 0.360 | 6.728 ± 0.360 | 1.000 | No significant comparisons | No significant comparisons |
|  | 30m | 6.239 ±  0.289 | 6.223 ± 1.292 | 0.983 |  |  |
|  | 2hr | 6.657 ±  0.315 | 6.806 ± 0.285 | 0.575 |  |  |
|  | 24hr | 6.652 ±  0.670 | 6.710 ± 0.514 | 0.910 |  |  |
| *Exiguobacterium* | 0hr | 0.556 ± 0.071 | 0.556 ± 0.071 | 1.000 | 24hr > 0hr,  p = 0.049 | No significant comparisons |
|  | 30m | 0.569 ±  0.067 | 0.550 ± 0.211 | 0.886 |  |  |
|  | 2hr | 0.663 ±  0.039 | 0.637 ± 0.043 | 0.487 |  |  |
|  | 24hr | 0.612 ±  0.036 | 0.718 ± 0.046 | 0.035 * |  |  |
| *Formosimonas* | 0hr | 0.541 ± 0.015 | 0.541 ± 0.015 | 1.000 | 24hr > 0hr,  p = 0.010  30min > 0hr,  p = 0.0340 | No significant comparisons |
|  | 30m | 0.539 ±  0.067 | 0.602 ± 0.008 | 0.295 |  |  |
|  | 2hr | 0.587 ±  0.027 | 0.588 ± 0.036 | 0.980 |  |  |
|  | 24hr | 0.548 ±  0.041 | 0.601 ± 0.009 | 0.094 |  |  |
| *Limnobacter* | 0hr | 0.391 ± 0.012 | 0.391 ± 0.012 | 1.000 | No significant comparisons | No significant comparisons |
|  | 30m | 0.375 ±  0.021 | 0.449 ± 0.077 | 0.186 |  |  |
|  | 2hr | 0.396 ±  0.024 | 0.387 ± 0.020 | 0.647 |  |  |
|  | 24hr | 0.388 ±  0.027 | 0.392 ± 0.009 | 0.836 |  |  |
| *Paenibacillus* | 0hr | 3.654 ± 0.017 | 3.654 ± 0.017 | 1.000 | No significant comparisons | No significant comparisons |
|  | 30m | 3.397 ±  0.356 | 3.326 ± 0.517 | 0.864 |  |  |
|  | 2hr | 3.623 ±  0.122 | 3.801 ± 0.091 | 0.119 |  |  |
|  | 24hr | 3.520 ±  0.145 | 3.800 ± 0.232 | 0.151 |  |  |
| *Phenylobacterium* | 0hr | 0.928 ± 0.015 | 0.928 ± 0.015 | 1.000 | No significant comparisons | No significant comparisons |
|  | 30m | 0.919 ±  0.010 | 0.918 ± 0.013 | 0.895 |  |  |
|  | 2hr | 0.947 ±  0.036 | 0.904 ± 0.037 | 0.197 |  |  |
|  | 24hr | 0.947 ±  0.036 | 0.964 ± 0.072 | 0..733 |  |  |
| *Polynucleobacter* | 0hr | 0.137 ± 0.011 | 0.137 ± 0.011 | 1.000 | No significant comparisons | No significant comparisons |
|  | 30m | 0.134 ±  0.006 | 0.134 ± 0.045 | 0.993 |  |  |
|  | 2hr | 0.157 ±  0.016 | 0.165 ± 0.027 | 0.558 |  |  |
|  | 24hr | 0.165 ±  0.030 | 0.172 ± 0.018 | 0.744 |  |  |
| *Pseudomonas* | 0hr | 0.262 ± 0.077 | 0.262 ± 0.077 | 1.000 | No significant comparisons | No significant comparisons |
|  | 30m | 0.217 ±  0.076 | 0.234 ± 0.116 | 0.857 |  |  |
|  | 2hr | 0.283 ±  0.041 | 0.310 ± 0.033 | 0.421 |  |  |
|  | 24hr | 0.251 ±  0.070 | 0.347 ± 0.029 | 0.093 |  |  |
| *Pseudotabrizicola* | 0hr | 0.764 ± 0.036 | 0.764 ± 0.036 | 1.000 | No significant comparisons | No significant comparisons |
|  | 30m | 0.807 ±  0.051 | 0.757 ± 0.038 | 0.325 |  |  |
|  | 2hr | 0.765 ±  0.025 | 0.723 ± 0.047 | 0.244 |  |  |
|  | 24hr | 0.755 ±  0.031 | 0.694 ± 0.030 | 0.420 |  |  |
| *Puniceibacterium* | 0hr | 0.231 ± 0.020 | 0.231 ± 0.020 | 1.000 | No significant comparisons | No significant comparisons |
|  | 30m | 0.236 ±  0.028 | 0.292 ± 0.102 | 0.397 |  |  |
|  | 2hr | 0.234 ±  0.004 | 0.234 ± 0.016 | 0.977 |  |  |
|  | 24hr | 0.239 ±  0.011 | 0.237 ± 0.004 | 0.798 |  |  |
| *Rhizobium* | 0hr | 2.136 ± 0.106 | 2.136 ± 0.106 | 1.000 | No significant comparisons | No significant comparisons |
|  | 30m | 2.092 ±  0.168 | 2.032 ± 0.330 | 0.797 |  |  |
|  | 2hr | 2.246 ±  0.097 | 2.258 ± 0.098 | 0.888 |  |  |
|  | 24hr | 2.112 ±  0.121 | 2.197 ± 0.016 | 0.295 |  |  |
| *Rhodococcus* | 0hr | 1.357 ± 0.149 | 1.357 ± 0.149 | 1.000 | No significant comparisons | No significant comparisons |
|  | 30m | 1.230 ±  0.234 | 1.293 ± 0.494 | 0.852 |  |  |
|  | 2hr | 1.406 ±  0.051 | 1.595 ± 0.94 | 0.038 * |  |  |
|  | 24hr | 1.357 ±  0.149 | 1.633 ± 0.070 | 0.048 * |  |  |
| *Sphingobium* | 0hr | 1.636 ± 0.034 | 1.636 ± 0.034 | 1.000 | No significant comparisons | No significant comparisons |
|  | 30m | 1.567 ±  0.081 | 1.672 ± 0.021 | 0.190 |  |  |
|  | 2hr | 1.648 ±  0.036 | 1.638 ± 0.031 | 0.728 |  |  |
|  | 24hr | 1.613 ±  0.020 | 1.626 ± 0.033 | 0.578 |  |  |
| *Stenotrophomonas* | 0hr | 2.686 ± 0.182 | 2.686 ± 0.182 | 1.000 | No significant comparisons | No significant comparisons |
|  | 30m | 2.711 ±  0.141 | 2.630 ± 0.369 | 0.738 |  |  |
|  | 2hr | 2.837 ±  0.054 | 2.827 ± 0.050 | 0.822 |  |  |
|  | 24hr | 2.712 ±  0.253 | 2.903 ± 0.025 | 0.264 |  |  |
| *Streptomyces* | 0hr | 3.959 ± 0.228 | 3.959 ± 0.228 | 1.000 | No significant comparisons | No significant comparisons |
|  | 30m | 3.848 ±  0.232 | 3.796 ± 0.250 | 0.823 |  |  |
|  | 2hr | 3.915 ±  0.132 | 3.955 ± 0.196 | 0.781 |  |  |
|  | 24hr | 3.869 ±  0.142 | 3.102 ± 1.347 | 0.382 |  |  |
| *Szabonella* | 0hr | 0.226 ± 0.008 | 0.226 ± 0.008 | 1.000 | No significant comparisons | No significant comparisons |
|  | 30m | 0.230 ±  0.015 | 0.201 ± 0.035 | 0.281 |  |  |
|  | 2hr | 0.217 ±  0.010 | 0.230 ± 0.027 | 0.482 |  |  |
|  | 24hr | 0.224 ±  0.022 | 0.233 ± 0.012 | 0.575 |  |  |

**Table S12:** Superoxide dismutase marker gene expression across June, August, and September sampling seasons, reported as the mean ± standard deviation of the superoxide dismutase marker gene:*rpoB* ratio. Unpaired *t*-tests were used to assess statistical differences across three comparison types: between treatments at each timepoint (hydrogen peroxide vs. control), within the hydrogen peroxide treatment time series, and within the control time series. Significance thresholds are indicated as follows: *p* < 0.05 = *, *p* < 0.005 = **, *p* < 0.0005 = ***. Note: the September time series does not include an 8-hour timepoint.

| **Pre Bloom Superoxide Dismutase Marker Genes:*rpoB* Expression** | | | | | | |
| --- | --- | --- | --- | --- | --- | --- |
| **Genus** | **Time** | **Control Ratio** | **H2O2 Ratio** | **T-Test Results (Comparing Treatments)** | **Significant**  **T-Test Results (Comparing H2O2)** | **Significant**  **T-Test Results (Comparing Control)** |
| *Aquirufa* | 0hr | 0.775 ± 0.082 | 0.775 ± 0.082 | 1.000 | No significant comparisons | No significant comparisons |
|  | 30m | 0.524 ±  0.425 | 0.597 ±  0.453 | 0.849 |  |  |
|  | 2hr | 0.413 ±  0.343 | 0.770 ±  0.012 | 0.258 |  |  |
|  | 8hr | 0.664 ±  0.183 | 0.753 ±  0.057 | 0.579 |  |  |
|  | 24hr | 0.652 ±  0.203 | 0.816 ±  0.011 | 0.373 |  |  |
| *Chitinilyticum* | 0hr | 0.359 ±  0.049 | 0.359 ±  0.049 | 1.000 | No significant comparisons | No significant comparisons |
|  | 30m | 0.277 ±  0.203 | 0.283 ±  0.243 | 0.977 |  |  |
|  | 2hr | 0.405 ±  0.199 | 0.381 ±  0.026 | 0.883 |  |  |
|  | 8hr | 0.875 ±  0.465 | 0.407 ±  0.003 | 0.291 |  |  |
|  | 24hr | 1.231 ±  1.235 | 0.294 ±  0.056 | 0.396 |  |  |
| *Chitinimonas* | 0hr | 0.062 ±  0.012 | 0.062 ±  0.012 | 1.000 | No significant comparisons | No significant comparisons |
|  | 30m | 0.059 ±  0.006 | 0.030 ±  0.027 | 0.146 |  |  |
|  | 2hr | 0.083 ±  0.030 | 0.049 ±  0.007 | 0.232 |  |  |
|  | 8hr | 0.068 ±  0.001 | 0.058 ±  0.003 | 0.029 * |  |  |
|  | 24hr | 0.154 ±  0.130 | 0.058 ±  0.005 | 0.406 |  |  |
| *Cupriavidus* | 0hr | 0.296 ±  0.072 | 0.296 ±  0.072 | 1.000 | No significant comparisons | No significant comparisons |
|  | 30m | 0.197 ±  0.137 | 0.139 ±  0.118 | 0.610 |  |  |
|  | 2hr | 0.229 ±  0.125 | 0.230 ±  0.028 | 0.987 |  |  |
|  | 8hr | 0.564 ±  0.358 | 0.238 ±  0.013 | 0.329 |  |  |
|  | 24hr | 0.976 ±  1.022 | 0.221 ±  0.004 | 0.406 |  |  |
| *Defluviimonas* | 0hr | 0.140 ±  0.002 | 0.140 ±  0.002 | 1.000 | No significant comparisons | No significant comparisons |
|  | 30m | 0.098 ±  0.085 | 0.116 ±  0.075 | 0.795 |  |  |
|  | 2hr | 0.092 ±  0.046 | 0.150 ±  0.020 | 0.205 |  |  |
|  | 8hr | 0.151 ±  0.008 | 0.146 ±  0.035 | 0.856 |  |  |
|  | 24hr | 0.174 ±  0.079 | 0.168 ±  0.015 | 0.928 |  |  |
| *Deinococcus* | 0hr | 0.673 ±  0.003 | 0.673 ±  0.003 | 1.000 | No significant comparisons | No significant comparisons |
|  | 30m | 0.463 ±  0.350 | 0.509 ±  0.302 | 0.871 |  |  |
|  | 2hr | 0.504 ±  0.347 | 0.666 ±  0.029 | 0.574 |  |  |
|  | 8hr | 0.701 ±  0.069 | 0.672 ±  0.004 | 0.616 |  |  |
|  | 24hr | 0.869 ±  0.286 | 0.659 ±  0.028 | 0.409 |  |  |
| *Enterobacter* | 0hr | 0.656 ±  0.064 | 0.656 ±  0.064 | 1.000 | 8hr > 2hr,  p = 0.041 | No significant comparisons |
|  | 30m | 0.491 ±  0.391 | 0.266 ±  0.248 | 0.447 |  |  |
|  | 2hr | 0.419 ±  0.286 | 0.443 ±  0.042 | 0.920 |  |  |
|  | 8hr | 0.510 ±  0.335 | 0.723 ±  0.071 | 0.472 |  |  |
|  | 24hr | 0.968 ±  0.325 | 0.625 ±  0.174 | 0.319 |  |  |
| *Escherichia* | 0hr | 0.540 ±  0.013 | 0.540 ±  0.013 | 1.000 | 2hr > 8hr,  p = 0.036 | No significant comparisons |
|  | 30m | 0.196 ±  0.295 | 0.373 ±  0.326 | 0.525 |  |  |
|  | 2hr | 0.313 ±  0.243 | 0.562 ±  0.006 | 0.263 |  |  |
|  | 8hr | 0.413 ±  0.095 | 0.498 ±  0.017 | 0.338 |  |  |
|  | 24hr | 0.435 ±  0.145 | 0.563 ±  0.016 | 0.342 |  |  |
| *Exiguobacterium* | 0hr | 0.296 ±  0.032 | 0.296 ±  0.032 | 1.000 | 24hr > 2hr,  p = 0.029 | No significant comparisons |
|  | 30m | 0.183 ±  0.109 | 0.235 ±  0.050 | 0.500 |  |  |
|  | 2hr | 0.164 ±  0.125 | 0.243 ±  0.012 | 0.456 |  |  |
|  | 8hr | 0.222 ±  0.004 | 0.257 ±  0.024 | 0.171 |  |  |
|  | 24hr | 0.194 ±  0.071 | 0.308 ±  0.010 | 0.156 |  |  |
| *Formosimonas* | 0hr | 0.156 ±  0.015 | 0.156 ±  0.015 | 1.000 | No significant comparisons | No significant comparisons |
|  | 30m | 0.096 ± 0.082 | 0.102 ±  0.088 | 0.939 |  |  |
|  | 2hr | 0.087 ±  0.076 | 0.147 ±  0.019 | 0.374 |  |  |
|  | 8hr | 0.161 ±  0.036 | 0.141 ±  0.016 | 0.549 |  |  |
|  | 24hr | 0.841 ±  0.956 | 0.151 ±  0.010 | 0.415 |  |  |
| *Limnobacter* | 0hr | 0.044 ±  0.003 | 0.044 ±  0.003 | 1.000 | 0hr > 8hr,  p = 0.022 | No significant comparisons |
|  | 30m | 0.070 ±  0.062 | 0.023 ±  0.019 | 0.277 |  |  |
|  | 2hr | 0.073 ±  0.039 | 0.039 ±  0.006 | 0.336 |  |  |
|  | 8hr | 0.060 ±  0.008 | 0.031 ± 2.57e-6 | 0.039* |  |  |
|  | 24hr | 0.315 ±  0.388 | 0.035 ±  0.005 | 0.414 |  |  |
| *Paenibacillus* | 0hr | 0.456 ±  0.023 | 0.456 ±  0.023 | 1.000 | No significant comparisons | No significant comparisons |
|  | 30m | 0.317 ±  0.258 | 0.318 ±  0.267 | 0.995 |  |  |
|  | 2hr | 0.305 ±  0.231 | 0.455 ±  0.004 | 0.450 |  |  |
|  | 8hr | 0.438 ±  0.001 | 0.435 ±  0.012 | 0.734 |  |  |
|  | 24hr | 0.404 ±  0.082 | 0.445 ±  0.009 | 0.554 |  |  |
| *Phenylobacterium* | 0hr | 0.549 ±  0.020 | 0.549 ±  0.020 | 1.000 | 8hr > 2hr,  p = 0.046 | No significant comparisons |
|  | 30m | 0.359 ±  0.311 | 0.426 ±  0.320 | 0.807 |  |  |
|  | 2hr | 0.322 ±  0.266 | 0.559 ±  0.017 | 0.319 |  |  |
|  | 8hr | 0.494 ±  0.085 | 0.503 ±  0.002 | 0.891 |  |  |
|  | 24hr | 0.367 ±  0.234 | 0.560 ±  0.020 | 0.366 |  |  |
| *Polynucleobacter* | 0hr | 0.074 ±  0.005 | 0.074 ±  0.005 | 1.000 | 0hr > 2hr,  p = 0.032 | No significant comparisons |
|  | 30m | 0.060 ±  0.015 | 0.033 ±  0.031 | 0.239 |  |  |
|  | 2hr | 0.074 ±  0.013 | 0.048 ±  0.004 | 0.085 |  |  |
|  | 8hr | 0.068 ±  0.029 | 0.078 ±  0.011 | 0.686 |  |  |
|  | 24hr | 0.096 ±  0.033 | 0.066 ±  0.016 | 0.376 |  |  |
| *Pseudomonas* | 0hr | 0.139 ±  0.006 | 0.139 ±  0.006 | 1.000 | 0hr > 2hr,  p = 0.031  0hr > 30min,  p = 0.017  24hr > 30min,  p = 0.044  8hr > 2hr,  p = 0.049  8hr > 30min,  p = 0.014 | 30min > 2hr,  p = 0.026 |
|  | 30m | 0.154 ±  0.011 | 0.097 ±  0.011 | 0.003 ** |  |  |
|  | 2hr | 0.112 ±  0.018 | 0.111 ± 0.004 | 0.981 |  |  |
|  | 8hr | 0.153 ±  0.010 | 0.152 ±  0.013 | 0.978 |  |  |
|  | 24hr | 0.225 ±  0.085 | 0.140 ±  0.019 | 0.302 |  |  |
| *Pseudotabrizicola* | 0hr | 0.065 ±  0.001 | 0.065 ±  0.001 | 1.000 | 2hr > 0hr,  p = 0.005  2hr > 24hr,  p = 0.036 | No significant comparisons |
|  | 30m | 0.049 ±  0.035 | 0.051 ±  0.044 | 0.950 |  |  |
|  | 2hr | 0.049 ±  0.042 | 0.080 ±  0.001 | 0.398 |  |  |
|  | 8hr | 0.076 ±  0.006 | 0.070 ±  0.006 | 0.459 |  |  |
|  | 24hr | 0.075 ±  0.005 | 0.063 ±  0.005 | 0.118 |  |  |
| *Puniceibacterium* | 0hr | 0.039 ±  0.008 | 0.039 ±  0.008 | 1.000 | No significant comparisons | No significant comparisons |
|  | 30m | 0.030 ±  0.027 | 0.027 ±  0.022 | 0.888 |  |  |
|  | 2hr | 0.032 ±  0.010 | 0.035 ±  0.002 | 0.671 |  |  |
|  | 8hr | 0.043 ±  0.004 | 0.045 ±  0.007 | 0.719 |  |  |
|  | 24hr | 0.059 ±  0.014 | 0.043 ±  0.004 | 0.269 |  |  |
| *Rhizobium* | 0hr | 0.133 ±  0.005 | 0.133 ±  0.005 | 1.000 | No significant comparisons | No significant comparisons |
|  | 30m | 0.088 ±  0.076 | 0.103 ±  0.076 | 0.815 |  |  |
|  | 2hr | 0.095 ±  0.065 | 0.139 ±  0.017 | 0.432 |  |  |
|  | 8hr | 0.130 ±  0.029 | 0.142 ±  0.007 | 0.614 |  |  |
|  | 24hr | 0.208 ±  0.119 | 0.145 ±  0.007 | 0.535 |  |  |
| *Rhodococcus* | 0hr | 0.732 ±  0.048 | 0.732 ±  0.048 | 1.000 | No significant comparisons | No significant comparisons |
|  | 30m | 0.479 ±  0.417 | 0.512 ±  0.386 | 0.927 |  |  |
|  | 2hr | 0.410 ±  0.325 | 0.715 ±  0.023 | 0.297 |  |  |
|  | 8hr | 0.531 ±  0.163 | 0.713 ±  0.026 | 0.259 |  |  |
|  | 24hr | 0.464 ±  0.260 | 0.695 ±  0.030 | 0.338 |  |  |
| *Sphingobium* | 0hr | 0.409 ±  0.005 | 0.409 ±  0.005 | 1.000 | No significant comparisons | No significant comparisons |
|  | 30m | 0.264 ±  0.229 | 0.269 ±  0.233 | 0.982 |  |  |
|  | 2hr | 0.237 ±  0.206 | 0.397 ±  0.003 | 0.374 |  |  |
|  | 8hr | 0.356 ±  0.482 | 0.397 ±  8.49e-6 | 0.347 |  |  |
|  | 24hr | 0.305 ±  0.111 | 0.438 ±  0.037 | 0.249 |  |  |
| *Stenotrophomonas* | 0hr | 0.727 ±  0.023 | 0.727 ±  0.023 | 1.000 | No significant comparisons | No significant comparisons |
|  | 30m | 0.506 ±  0.427 | 0.556 ±  0.363 | 0.885 |  |  |
|  | 2hr | 0.471 ±  0.373 | 0.737 ±  0.014 | 0.410 |  |  |
|  | 8hr | 0.645 ±  0.191 | 0.805 ±  0.040 | 0.367 |  |  |
|  | 24hr | 0.580 ±  0.223 | 0.726 ±  0.039 | 0.456 |  |  |
| *Streptomyces* | 0hr | 4.633 ±  0.318 | 4.633 ±  0.318 | 1.000 | 2hr > 8hr,  p = 0.048 | No significant comparisons |
|  | 30m | 2.927 ±  2.557 | 3.274 ±  2.784 | 0.881 |  |  |
|  | 2hr | 2.725 ±  2.391 | 4.699 ±  0.100 | 0.349 |  |  |
|  | 8hr | 3.467 ±  1.159 | 4.309 ±  0.075 | 0.413 |  |  |
|  | 24hr | 2.614 ±  2.323 | 4.450 ±  0.169 | 0.381 |  |  |
| *Szabonella* | 0hr | 0.047 ±  4.65e-4 | 0.047 ±  4.65e-4 | 1.000 | No significant comparisons | No significant comparisons |
|  | 30m | 0.030 ±  0.026 | 0.034 ±  0.028 | 0.883 |  |  |
|  | 2hr | 0.038 ±  0.029 | 0.050 ±  0.002 | 0.621 |  |  |
|  | 8hr | 0.054 ±  0.009 | 0.053 ±  0.007 | 0.877 |  |  |
|  | 24hr | 0.092 ±  0.050 | 0.043 ±  0.008 | 0.304 |  |  |
| **Peak Bloom Superoxide Dismutase Marker Gene:*rpoB* Expression** | | | | | | |
| **Genus** | **Time** | **Control Ratio** | **H2O2 Ratio** | **T-Test Results (Comparing Treatments)** | **Significant**  **T-Test Results (Comparing H2O2)** | **Significant**  **T-Test Results (Comparing Control)** |
| *Aquirufa* | 0hr | 0.576 ±  0.478 | 0.576 ±  0.478 | 1.000 | No significant comparisons | No significant comparisons |
|  | 30m | 0.318 ±  0.461 | 0.046 ±  0.064 | 0.368 |  |  |
|  | 2hr | 0.035 ±  0.033 | 0.884 ±  0.665 | 0.326 |  |  |
|  | 8hr | 0.035 ±  0.030 | 0.156 ±  0.270 | 0.485 |  |  |
|  | 24hr | 0.286 ±  0.466 | 0.458 ±  0.295 | 0.617 |  |  |
| *Chitinilyticum* | 0hr | 0.181 ±  0.116 | 0.181 ±  0.116 | 1.000 | 24hr > 2hr,  p = 0.009  24hr > 30min,  p = 0.004 | No significant comparisons |
|  | 30m | 0.138 ±  0.117 | 0.043 ±  0.038 | 0.254 |  |  |
|  | 2hr | 0.126 ±  0.080 | 0.035 ±  0.052 | 0.172 |  |  |
|  | 8hr | 0.062 ±  0.031 | 0.102 ±  0.163 | 0.700 |  |  |
|  | 24hr | 0.162 ±  0.117 | 0.195 ±  0.025 | 0.652 |  |  |
| *Chitinimonas* | 0hr | 0.025 ±  0.018 | 0.025 ±  0.018 | 1.000 | 24hr > 2hr,  p = 0.002  24hr > 30min,  p = 0.002 | No significant comparisons |
|  | 30m | 0.012 ±  0.017 | 0.004 ±  0.004 | 0.495 |  |  |
|  | 2hr | 0.022 ±  0.007 | 0.000 ±  0.000 | 0.006 ** |  |  |
|  | 8hr | 0.074 ±  0.097 | 0.033 ±  0.056 | 0.561 |  |  |
|  | 24hr | 0.037 ±  0.035 | 0.061 ±  0.014 | 0.333 |  |  |
| *Cupriavidus* | 0hr | 0.230 ±  0.089 | 0.230 ±  0.089 | 1.000 | 0hr > 30min,  p = 0.038  24hr > 2hr,  p = 0.021  24hr > 30min,  p = 0.011 | 0hr > 8hr,  p = 0.047  24hr > 8hr,  p = 0.037 |
|  | 30m | 0.136 ±  0.092 | 0.067 ±  0.026 | 0.276 |  |  |
|  | 2hr | 0.280 ±  0.235 | 0.099 ±  0.025 | 0.254 |  |  |
|  | 8hr | 0.076 ±  0.029 | 0.127 ±  0.159 | 0.611 |  |  |
|  | 24hr | 0.282 ±  0.112 | 0.237 ±  0.060 | 0.578 |  |  |
| *Defluviimonas* | 0hr | 0.201 ±  0.050 | 0.201 ±  0.050 | 1.000 | 24hr > 8hr,  p = 0.026 | 0hr > 8hr,  p = 0.013 |
|  | 30m | 0.070 ±  0.067 | 0.331 ±  0.380 | 0.307 |  |  |
|  | 2hr | 0.143 ±  0.073 | 0.254 ±  0.169 | 0.355 |  |  |
|  | 8hr | 0.060 ±  0.28 | 0.081 ±  0.068 | 0.654 |  |  |
|  | 24hr | 0.357 ±  0.401 | 0.280 ±  0.073 | 0.760 |  |  |
| *Deinococcus* | 0hr | 0.498 ±  0.357 | 0.498 ±  0.357 | 1.000 | No significant comparisons | No significant comparisons |
|  | 30m | 0.279 ±  0.366 | 0.229 ±  0.077 | 0.829 |  |  |
|  | 2hr | 0.137 ±  0.066 | 0.223 ±  0.196 | 0.513 |  |  |
|  | 8hr | 0.088 ±  0.019 | 0.317 ±  0.121 | 0.032 * |  |  |
|  | 24hr | 0.291 ±  0.342 | 0.257 ±  0.030 | 0.873 |  |  |
| *Enterobacter* | 0hr | 0.226 ±  0.208 | 0.226 ±  0.208 | 1.000 | 24hr > 30min,  p = 0.016 | No significant comparisons |
|  | 30m | 0.306 ±  0.404 | 0.047 ±  0.023 | 0.331 |  |  |
|  | 2hr | 0.174 ±  0.110 | 0.334 ±  0.550 | 0.647 |  |  |
|  | 8hr | 0.079 ±  0.053 | 0.398 ±  0.447 | 0.288 |  |  |
|  | 24hr | 0.202 ±  0.154 | 0.188 ±  0.057 | 0.890 |  |  |
| *Escherichia* | 0hr | 0.387 ±  0.245 | 0.387 ±  0.245 | 1.000 | No significant comparisons | No significant comparisons |
|  | 30m | 0.173 ±  0.280 | 0.142 ±  0.242 | 0.892 |  |  |
|  | 2hr | 0.084 ±  0.030 | 0.123 ±  0.181 | 0.727 |  |  |
|  | 8hr | 0.058 ±  0.050 | 0.160 ±  0.150 | 0.326 |  |  |
|  | 24hr | 0.199 ±  0.242 | 0.106 ±  0.050 | 0.552 |  |  |
| *Exiguobacterium* | 0hr | 0.158 ±  0.120 | 0.158 ±  0.120 | 1.000 | 24hr > 2hr,  p = 0.019 | No significant comparisons |
|  | 30m | 0.102 ±  0.122 | 0.082 ±  0.105 | 0.844 |  |  |
|  | 2hr | 0.045 ±  0.023 | 0.019 ±  0.013 | 0.161 |  |  |
|  | 8hr | 0.056 ±  0.051 | 0.031 ±  0.044 | 0.547 |  |  |
|  | 24hr | 0.093 ±  0.110 | 0.086 ±  0.027 | 0.921 |  |  |
| *Formosimonas* | 0hr | 0.103 ±  0.066 | 0.103 ±  0.066 | 1.000 | 24hr > 2hr,  p = 0.000473  24hr > 30min,  p = 0.000623 | No significant comparisons |
|  | 30m | 0.056 ±  0.074 | 0.006 ±  0.005 | 0.308 |  |  |
|  | 2hr | 0.053 ±  0.055 | 0.000 ±  0.000 | 0.169 |  |  |
|  | 8hr | 0.028 ±  0.045 | 0.079 ±  0.111 | 0.507 |  |  |
|  | 24hr | 0.049 ±  0.084 | 0.126 ±  0.021 | 0.199 |  |  |
| *Limnobacter* | 0hr | 0.049 ±  0.024 | 0.049 ±  0.024 | 1.000 | 24hr > 0hr,  p = 0.043  0hr > 30min,  p = 0.040  24hr > 2hr,  p = 0.035  24hr > 30min,  p = 0.013 | 2hr > 30min,  p = 0.017  2hr > 8hr,  p = 0.016 |
|  | 30m | 0.013 ±  0.017 | 0.007 ±  0.003 | 0.568 |  |  |
|  | 2hr | 0.095 ±  0.032 | 0.025 ±  0.043 | 0.083 |  |  |
|  | 8hr | 0.018 ±  0.010 | 0.065 ±  0.059 | 0.242 |  |  |
|  | 24hr | 0.170 ±  0.164 | 0.162 ±  0.063 | 0.940 |  |  |
| *Paenibacillus* | 0hr | 0.409 ±  0.310 | 0.409 ±  0.310 | 1.000 | 24hr > 2hr,  p = 0.011  24hr > 30min,  p = 0.008 | No significant comparisons |
|  | 30m | 0.186 ±  0.314 | 0.057 ± 0.049 | 0.521 |  |  |
|  | 2hr | 0.73 ±  0.066 | 0.061 ±  0.077 | 0.850 |  |  |
|  | 8hr | 0.041 ±  0.030 | 0.203 ±  0.254 | 0.333 |  |  |
|  | 24hr | 0.230 ±  0.342 | 0.492 ±  0.146 | 0.289 |  |  |
| *Phenylobacterium* | 0hr | 0.369 ±  0.309 | 0.369 ±  0.309 | 1.000 | No significant comparisons | No significant comparisons |
|  | 30m | 0.170 ±  0.291 | 0.008 ±  0.007 | 0.390 |  |  |
|  | 2hr | 0.040 ±  0.006 | 0.003 ±  0.003 | 0.001 ** |  |  |
|  | 8hr | 0.018 ±  0.017 | 0.052 ±  0.068 | 0.445 |  |  |
|  | 24hr | 0.217 ±  0.354 | 0.210 ±  0.191 | 0.977 |  |  |
| *Polynucleobacter* | 0hr | 0.042 ±  0.008 | 0.042 ±  0.008 | 1.000 | 24hr > 0hr,  p = 0.012  24hr > 2hr,  p = 0.011 | 0hr > 8hr,  p = 0.014  2hr > 8hr,  p = 0.009 |
|  | 30m | 0.024 ±  0.040 | 0.038 ±  0.036 | 0.669 |  |  |
|  | 2hr | 0.067 ±  0.018 | 0.022 ±  0.019 | 0.039 * |  |  |
|  | 8hr | 0.010 ±  0.010 | 0.059 ±  0.084 | 0.374 |  |  |
|  | 24hr | 0.071 ±  0.058 | 0.081 ±  0.013 | 0.772 |  |  |
| *Pseudomonas* | 0hr | 0.240 ±  0.286 | 0.240 ±  0.286 | 1.000 | No significant comparisons | No significant comparisons |
|  | 30m | 0.218 ±  0.096 | 0.436 ±  0.235 | 0.211 |  |  |
|  | 2hr | 0.370 ±  0.054 | 0.520 ±  0.438 | 0.587 |  |  |
|  | 8hr | 0.215 ±  0.093 | 0.228 ±  0.232 | 0.935 |  |  |
|  | 24hr | 0.221 ±  0.261 | 0.143 ±  0.058 | 0.640 |  |  |
| *Pseudotabrizicola* | 0hr | 0.090 ±  0.044 | 0.090 ±  0.044 | 1.000 | 0hr > 2hr,  p = 0.030  24hr > 2hr,  p = 0.004 | No significant comparisons |
|  | 30m | 0.046 ±  0.058 | 0.069 ±  0.110 | 0.768 |  |  |
|  | 2hr | 0.027 ±  0.014 | 0.006 ±  0.010 | 0.094 |  |  |
|  | 8hr | 0.020 ±  0.019 | 0.025 ± 0.042 | 0.838 |  |  |
|  | 24hr | 0.041 ±  0.052 | 0.092 ±  0.024 | 0.199 |  |  |
| *Puniceibacterium* | 0hr | 0.129 ±  0.076 | 0.129 ±  0.076 | 1.000 | 24hr > 2hr,  p = 0.004  24hr > 8hr,  p = 0.015 | 2hr > 8hr,  p = 0.006 |
|  | 30m | 0.098 ±  0.094 | 0.103 ± 0.098 | 0.959 |  |  |
|  | 2hr | 0.101 ±  0.015 | 0.044 ±  0.027 | 0.034 * |  |  |
|  | 8hr | 0.044 ±  0.012 | 0.034 ±  0.050 | 0.745 |  |  |
|  | 24hr | 0.058 ±  0.054 | 0.159 ±  0.020 | 0.038 |  |  |
| *Rhizobium* | 0hr | 0.110 ±  0.052 | 0.110 ±  0.052 | 1.000 | 24hr > 2hr,  p = 0.006 | 2hr > 8hr,  p = 0.035 |
|  | 30m | 0.070 ±  0.056 | 0.115 ±  0.126 | 0.596 |  |  |
|  | 2hr | 0.084 ±  0.018 | 0.030 ±  0.026 | 0.039 * |  |  |
|  | 8hr | 0.030 ±  0.025 | 0.096 ±  0.074 | 0.211 |  |  |
|  | 24hr | 0.086 ±  0.072 | 0.168 ±  0.038 | 0.159 |  |  |
| *Rhodococcus* | 0hr | 0.560 ±  0.393 | 0.560 ±  0.393 | 1.000 | No significant comparisons | No significant comparisons |
|  | 30m | 0.291 ±  0.481 | 0.093 ±  0.146 | 0.532 |  |  |
|  | 2hr | 0.037 ±  0.036 | 0.132 ±  0.223 | 0.504 |  |  |
|  | 8hr | 0.015 ±  0.012 | 0.076 ±  0.108 | 0.387 |  |  |
|  | 24hr | 0.277 ±  0.455 | 0.186 ±  0.097 | 0.753 |  |  |
| *Sphingobium* | 0hr | 0.289 ±  0.236 | 0.289 ±  0.236 | 1.000 | No significant comparisons | 2hr > 8hr,  p = 0.033 |
|  | 30m | 0.130 ±  0.224 | 0.003 ±  0.003 | 0.383 |  |  |
|  | 2hr | 0.013 ±  0.006 | 0.000 ±  0.000 | 0.017 * |  |  |
|  | 8hr | 0.002 ±  0.002 | 0.051 ±  0.089 | 0.391 |  |  |
|  | 24hr | 0.147 ±  0.225 | 0.185 ±  0.127 | 0.811 |  |  |
| *Stenotrophomonas* | 0hr | 0.607 ±  0.397 | 0.607 ±  0.397 | 1.000 | 24hr > 30min,  p = 0.008 | No significant comparisons |
|  | 30m | 0.321 ±  0.484 | 0.075 ±  0.042 | 0.429 |  |  |
|  | 2hr | 0.111 ±  0.085 | 0.225 ± 0.322 | 0.585 |  |  |
|  | 8hr | 0.109 ±  0.012 | 0.197 ±  0.296 | 0.635 |  |  |
|  | 24hr | 0.380 ±  0.418 | 0.305 ±  0.070 | 0.776 |  |  |
| *Streptomyces* | 0hr | 2.436 ±  2.092 | 2.436 ±  2.092 | 1.000 | No significant comparisons | No significant comparisons |
|  | 30m | 0.815 ±  1.404 | 0.033 ±  0.049 | 0.390 |  |  |
|  | 2hr | 0.030 ±  0.019 | 0.038 ±  0.056 | 0.818 |  |  |
|  | 8hr | 0.006 ±  0.004 | 0.103 ±  0.124 | 0.249 |  |  |
|  | 24hr | 1.265 ±  2.175 | 0.774 ±  1.063 | 0.743 |  |  |
| *Szabonella* | 0hr | 0.168 ±  0.181 | 0.168 ±  0.181 | 1.000 | No significant comparisons | No significant comparisons |
|  | 30m | 0.056 ±  0.028 | 0.158 ±  0.111 | 0.199 |  |  |
|  | 2hr | 0.132 ±  0.097 | 0.348 ±  0.552 | 0.541 |  |  |
|  | 8hr | 0.071 ±  0.014 | 0.069 ±  0.067 | 0.953 |  |  |
|  | 24hr | 0.105 ±  0.071 | 0.156 ±  0.028 | 0.316 |  |  |
| **Bloom Decline Superoxide Dismutase Marker Genes:*rpoB* Expression** | | | | | | |
| **Genus** | **Time** | **Control Ratio** | **H2O2 Ratio** | **T-Test Results (Comparing Treatments)** | **Significant**  **T-Test Results (Comparing H2O2)** | **Significant**  **T-Test Results (Comparing Control)** |
| *Aquirufa* | 0hr | 0.837 ±  0.049 | 0.837 ±  0.049 | 1.000 | No significant comparisons | No significant comparisons |
|  | 30m | 0.863 ±  0.045 | 0.793 ±  0.061 | 0.226 |  |  |
|  | 2hr | 0.851 ±  0.021 | 0.846 ±  0.019 | 0.773 |  |  |
|  | 24hr | 0.839 ±  0.022 | 0.858 ±  0.025 | 0.379 |  |  |
| *Chitinilyticum* | 0hr | 0.257 ±  2.03e-4 | 0.257 ±  2.03e-4 | 1.000 | No significant comparisons | 24hr > 0hr,  p = 0.016 |
|  | 30m | 0.345 ±  0.109 | 0.334 ±  0.119 | 0.920 |  |  |
|  | 2hr | 0.274 ±  0.030 | 0.265 ±  0.007 | 0.620 |  |  |
|  | 24hr | 0.287 ±  0.008 | 0.255 ±  0.016 | 0.036* |  |  |
| *Chitinimonas* | 0hr | 0.037 ±  0.002 | 0.037 ±  0.002 | 1.000 | No significant comparisons | No significant comparisons |
|  | 30m | 0.039 ±  0.004 | 0.041 ±  0.005 | 0.585 |  |  |
|  | 2hr | 0.038 ±  6.4e-4 | 0.036 ±  0.001 | 0.059 |  |  |
|  | 24hr | 0.039 ±  0.001 | 0.037 ±  0.001 | 0.120 |  |  |
| *Cupriavidus* | 0hr | 0.185 ±  0.033 | 0.185 ±  0.033 | 1.000 | No significant  comparisons | No significant  comparisons |
|  | 30m | 0.247 ±  0.092 | 0.233 ±  0.097 | 0.878 |  |  |
|  | 2hr | 0.178 ±  0.009 | 0.171 ±  0.006 | 0.327 |  |  |
|  | 24hr | 0.174 ±  0.006 | 0.153 ±  0.020 | 0.144 |  |  |
| *Defluviimonas* | 0hr | 0.154 ±  0.005 | 0.154 ±  0.005 | 1.000 | No significant comparisons | No significant comparisons |
|  | 30m | 0.175 ±  0.020 | 0.186 ±  0.040 | 0.695 |  |  |
|  | 2hr | 0.159 ±  0.012 | 0.166 ±  0.006 | 0.418 |  |  |
|  | 24hr | 0.179 ±  0.014 | 0.158 ±  0.015 | 0.147 |  |  |
| *Deinococcus* | 0hr | 0.710 ±  0.005 | 0.710 ±  0.005 | 1.000 | No significant comparisons | No significant comparisons |
|  | 30m | 0.702 ±  0.031 | 0.694 ±  0.006 | 0.744 |  |  |
|  | 2hr | 0.701 ±  0.026 | 0.705 ±  0.005 | 0.795 |  |  |
|  | 24hr | 0.706 ±  0.011 | 0.704 ±  0.015 | 0.838 |  |  |
| *Enterobacter* | 0hr | 0.433 ±  0.033 | 0.433 ±  0.033 | 1.000 | 0hr > 24hr,  p = 0.033 | 2hr > 30min,  p = 0.034 |
|  | 30m | 0.406 ±  0.051 | 0.417 ±  0.189 | 0.921 |  |  |
|  | 2hr | 0.521 ±  0.037 | 0.483 ±  0.082 | 0.504 |  |  |
|  | 24hr | 0.620 ±  0.357 | 0.575 ±  0.045 | 0.841 |  |  |
| *Escherichia* | 0hr | 0.506 ±  0.008 | 0.506 ±  0.008 | 1.000 | No significant comparisons | No significant comparisons |
|  | 30m | 0.516 ±  0.007 | 0.562 ±  0.037 | 0.106 |  |  |
|  | 2hr | 0.514 ±  0.021 | 0.514 ±  0.040 | 0.990 |  |  |
|  | 24hr | 0.508 ± 0.026 | 0.523 ±  0.061 | 0.712 |  |  |
| *Exiguobacterium* | 0hr | 0.177 ±  0.008 | 0.177 ±  0.008 | 1.000 | 0hr > 24hr,  p = 0.002  24hr > 2hr,  p = 0.045 | No significant comparisons |
|  | 30m | 0.180 ±  0.032 | 0.174 ±  0.057 | 0.879 |  |  |
|  | 2hr | 0.205 ±  0.023 | 0.211 ±  0.023 | 0.757 |  |  |
|  | 24hr | 0.177 ±  0.046 | 0.250 ±  0.008 | 0.051 |  |  |
| *Formosimonas* | 0hr | 0.138 ±  0.018 | 0.138 ±  0.018 | 1.000 | No significant comparisons | No significant comparisons |
|  | 30m | 0.128 ±  0.017 | 0.141 ±  0.002 | 0.350 |  |  |
|  | 2hr | 0.140 ±  0.007 | 0.142 ±  0.023 | 0.865 |  |  |
|  | 24hr | 0.125 ±  0.026 | 0.143 ±  0.010 | 0.325 |  |  |
| *Limnobacter* | 0hr | 0.034 ±  0.005 | 0.034 ±  0.005 | 1.000 | No significant comparisons | No significant comparisons |
|  | 30m | 0.034 ±  0.004 | 0.041 ±  0.009 | 0.341 |  |  |
|  | 2hr | 0.035 ±  0.002 | 0.035 ±  0.004 | 0.963 |  |  |
|  | 24hr | 0.038 ±  0.005 | 0.037 ±  0.004 | 0.781 |  |  |
| *Paenibacillus* | 0hr | 0.594 ±  0.028 | 0.594 ±  0.028 | 1.000 | No significant comparisons | No significant comparisons |
|  | 30m | 0.594 ±  0.047 | 0.581 ±  0.044 | 0.780 |  |  |
|  | 2hr | 0.572 ±  0.015 | 0.583 ±  0.026 | 0.547 |  |  |
|  | 24hr | 0.589 ±  0.006 | 0.574 ±  0.014 | 0.172 |  |  |
| *Phenylobacterium* | 0hr | 0.554 ±  0.054 | 0.554 ±  0.054 | 1.000 | No significant comparisons | 24hr > 0hr,  p = 0.032 |
|  | 30m | 0.589 ±  0.012 | 0.574 ±  0.012 | 0.270 |  |  |
|  | 2hr | 0.558 ±  0.025 | 0.566 ±  0.041 | 0.783 |  |  |
|  | 24hr | 0.560 ±  0.009 | 0.563 ±  0.021 | 0.834 |  |  |
| *Polynucleobacter* | 0hr | 0.059 ±  0.025 | 0.059 ±  0.025 | 1.000 | No significant comparisons | 24hr > 2hr,  p = 0.004 |
|  | 30m | 0.073 ±  0.031 | 0.075 ±  0.035 | 0.958 |  |  |
|  | 2hr | 0.056 ±  0.009 | 0.050 ±  0.005 | 0.381 |  |  |
|  | 24hr | 0.088 ±  0.004 | 0.049 ±  0.003 | 0.0001 *** |  |  |
| *Pseudomonas* | 0hr | 0.071 ±  0.014 | 0.071 ±  0.014 | 1.000 | No significant comparisons | No significant comparisons |
|  | 30m | 0.070 ±  0.010 | 0.078 ±  0.011 | 0.477 |  |  |
|  | 2hr | 0.083 ±  0.016 | 0.083 ±  0.009 | 0.997 |  |  |
|  | 24hr | 0.074 ±  0.012 | 0.093 ±  0.006 | 0.068 |  |  |
| *Pseudotabrizicola* | 0hr | 0.120 ±  0.010 | 0.120 ±  0.010 | 1.000 | 24hr > 30min,  p = 0.031 | No significant comparisons |
|  | 30m | 0.116 ±  0.010 | 0.102 ±  0.004 | 0.185 |  |  |
|  | 2hr | 0.118 ±  0.007 | 0.110 ±  0.014 | 0.425 |  |  |
|  | 24hr | 0.113 ±  0.007 | 0.114 ±  0.004 | 0.816 |  |  |
| *Puniceibacterium* | 0hr | 0.050 ±  0.001 | 0.050 ±  0.001 | 1.000 | 0hr > 30min,  p = 0.025 | No significant comparisons |
|  | 30m | 0.047 ±  0.001 | 0.062 ±  0.003 | 0.004 ** |  |  |
|  | 2hr | 0.048 ±  0.005 | 0.051 ±  0.005 | 0.558 |  |  |
|  | 24hr | 0.056 ±  0.008 | 0.058 ±  0.008 | 0.782 |  |  |
| *Rhizobium* | 0hr | 0.132 ±  0.005 | 0.132 ±  0.005 | 1.000 | No significant comparisons | No significant comparisons |
|  | 30m | 0143 ±  0.003 | 0.141 ±  0.001 | 0.467 |  |  |
|  | 2hr | 0.138 ±  0.002 | 0.138 ±  0.005 | 0.956 |  |  |
|  | 24hr | 0.138 ±  0.015 | 0.132 ±  0.011 | 0.592 |  |  |
| *Rhodococcus* | 0hr | 0.787 ±  0.082 | 0.787 ±  0.082 | 1.000 | No significant comparisons | No significant comparisons |
|  | 30m | 0.778 ±  0.022 | 0.765 ±  0.113 | 0.850 |  |  |
|  | 2hr | 0.774 ±  0.040 | 0.832 ±  0.034 | 0.133 |  |  |
|  | 24hr | 0.770 ±  0.116 | 0.828 ±  0.016 | 0.440 |  |  |
| *Sphingobium* | 0hr | 0.415 ±  0.013 | 0.415 ±  0.013 | 1.000 | No significant comparisons | No significant comparisons |
|  | 30m | 0.426 ±  0.017 | 0.419 ±  0.009 | 0.632 |  |  |
|  | 2hr | 0.431 ±  0.026 | 0.415 ±  0.024 | 0.473 |  |  |
|  | 24hr | 0.418 ±  0.020 | 0.405 ±  0.013 | 0.415 |  |  |
| *Stenotrophomonas* | 0hr | 0.874 ±  0.079 | 0.874 ±  0.079 | 1.000 | No significant comparisons | No significant comparisons |
|  | 30m | 0.862 ±  0.033 | 0.844 ±  0.044 | 0.629 |  |  |
|  | 2hr | 0.856 ±  0.020 | 0.853 ±  0.005 | 0.846 |  |  |
|  | 24hr | 0.848 ±  0.024 | 0.870 ±  0.034 | 0.415 |  |  |
| *Streptomyces* | 0hr | 3.426 ±  0.214 | 3.426 ±  0.214 | 1.000 | No significant comparisons | No significant comparisons |
|  | 30m | 3.561 ±  0.295 | 3.310 ±  0.219 | 0.385 |  |  |
|  | 2hr | 3.666 ±  0.482 | 3.261 ±  0.405 | 0.327 |  |  |
|  | 24hr | 3.413 ±  0.170 | 2.441 ±  0.936 | 0.152 |  |  |
| *Szabonella* | 0hr | 0.061 ±  0.004 | 0.061 ±  0.004 | 1.000 | No significant comparisons | No significant comparisons |
|  | 30m | 0.065 ±  0.004 | 0.073 ±  0.005 | 0.164 |  |  |
|  | 2hr | 0.060 ±  0.005 | 0.064 ±  0.007 | 0.440 |  |  |
|  | 24hr | 0.066 ±  0.007 | 0.066 ±  0.005 | 0.981 |  |  |

**Table S13:** Alkyl hydroperoxide reductase marker gene expression across June, August, and September sampling seasons, reported as the mean ± standard deviation of the alkyl hydroperoxide reductase marker gene:*rpoB* ratio. Unpaired *t*-tests were used to assess statistical differences across three comparison types: between treatments at each timepoint (hydrogen peroxide vs. control), within the hydrogen peroxide treatment time series, and within the control time series. Significance thresholds are indicated as follows: *p* < 0.05 = *, *p* < 0.005 = **, *p* < 0.0005 = ***. Note: the September time series does not include an 8-hour timepoint. *Puniceibacterium* and *Szabonella* were excluded due to the absence of genes encoding for production of this enzyme.

| **Pre Bloom Alkyl Hydroperoxide Reductase Marker Genes:*rpoB* Expression** | | | | | | |
| --- | --- | --- | --- | --- | --- | --- |
| **Genus** | **Time** | **Control Ratio** | **H2O2 Ratio** | **T-Test Results (Comparing Treatments)** | **Significant**  **T-Test Results (Comparing H2O2)** | **Significant**  **T-Test Results (Comparing Control)** |
| *Aquirufa* | 0hr | 0.170 ±  0.002 | 0.170 ±  0.002 | 1.000 | 2hr > 0hr,  p = 0.047 | 0hr > 24hr,  p = 0.028 |
|  | 30m | 0.096 ±  0.083 | 0.110 ±  0.081 | 0.836 |  |  |
|  | 2hr | 0.071 ±  0.061 | 0.146 ±  0.007 | 0.203 |  |  |
|  | 8hr | 0.109 ±  0.048 | 0.147 ±  0.016 | 0.404 |  |  |
|  | 24hr | 0.036 ±  0.032 | 0.148 ±  0.010 | 0.043 * |  |  |
| *Chitinilyticum* | 0hr | 0.394 ±  0.051 | 0.394 ±  0.051 | 1.000 | 8hr > 24hr,  p = 0.023 | No significant comparisons |
|  | 30m | 0.363 ±  0.280 | 0.523 ±  0.148 | 0.431 |  |  |
|  | 2hr | 0.315 ±  0.147 | 0.432 ±  0.039 | 0.371 |  |  |
|  | 8hr | 0.462 ±  0.115 | 0.491 ±  0.015 | 0.764 |  |  |
|  | 24hr | 0.738 ±  0.354 | 0.371 ±  0.026 | 0.282 |  |  |
| *Chitinimonas* | 0hr | 0.022 ±  0.006 | 0.022 ±  0.006 | 1.000 | No significant comparisons | No significant comparisons |
|  | 30m | 0.015 ±  0.013 | 0.011 ±  0.010 | 0.738 |  |  |
|  | 2hr | 0.018 ±  0.006 | 0.019 ±  6.37e-5 | 0.748 |  |  |
|  | 8hr | 0.023 ±  0.003 | 0.022 ±  0.001 | 0.669 |  |  |
|  | 24hr | 0.147 ±  0.173 | 0.023 ±  0.003 | 0.419 |  |  |
| *Cupriavidus* | 0hr | 0.169 ±  0.008 | 0.169 ±  0.008 | 1.000 | No significant comparisons | No significant comparisons |
|  | 30m | 0.107 ±  0.095 | 0.092 ±  0.061 | 0.839 |  |  |
|  | 2hr | 0.125 ±  0.061 | 0.129 ±  0.022 | 0.423 |  |  |
|  | 8hr | 0.130 ±  0.031 | 0.150 ±  0.003 | 0.466 |  |  |
|  | 24hr | 0.169 ±  0.008 | 0.143 ±  0.015 | 0.733 |  |  |
| *Defluviimonas* | 0hr | 0.087 ±  0.013 | 0.087 ±  0.013 | 1.000 | No significant comparisons | No significant comparisons |
|  | 30m | 0.073 ±  0.063 | 0.078 ±  0.055 | 0.913 |  |  |
|  | 2hr | 0.059 ±  0.051 | 0.109 ±  0.002 | 0.273 |  |  |
|  | 8hr | 0.094 ±  0.018 | 0.095 ±  0.015 | 0.973 |  |  |
|  | 24hr | 0.094 ±  0.052 | 0.115 ±  0.009 | 0.636 |  |  |
| *Deinococcus* | 0hr | 0.208 ±  0.023 | 0.208 ±  0.023 | 1.000 | 2hr > 8hr,  p = 0.023 | No significant comparisons |
|  | 30m | 0.138 ±  0.120 | 0.149 ±  0.129 | 0.922 |  |  |
|  | 2hr | 0.133 ±  0.115 | 0.215 ±  0.001 | 0.411 |  |  |
|  | 8hr | 0.193 ±  0.015 | 0.220 ±  0.001 | 0.132 |  |  |
|  | 24hr | 0.157 ±  0.074 | 0.221 ±  0.003 | 0.346 |  |  |
| *Enterobacter* | 0hr | 0.337 ±  0.041 | 0.337 ±  0.041 | 1.000 | 0hr > 2hr,  p = 0.045  8hr > 30min,  p = 0.047 | No significant comparisons |
|  | 30m | 0.251 ±  1.600 | 0.192 ±  0.056 | 0.581 |  |  |
|  | 2hr | 0.218 ±  0.160 | 0.206 ±  0.005 | 0.929 |  |  |
|  | 8hr | 0.264 ±  1.630 | 0.353 ±  0.049 | 0.534 |  |  |
|  | 24hr | 0.470 ±  0.151 | 0.303 ±  0.072 | 0.295 |  |  |
| *Escherichia* | 0hr | 0.630 ±  0.044 | 0.630 ±  0.044 | 1.000 | 24hr > 8hr,  p = 0.036 | No significant comparisons |
|  | 30m | 0.408 ±  0.349 | 0.544 ±  0.306 | 0.638 |  |  |
|  | 2hr | 0.341 ±  0.298 | 0.640 ±  0.009 | 0.272 |  |  |
|  | 8hr | 0.439 ±  0.123 | 0.572 ±  0.021 | 0.270 |  |  |
|  | 24hr | 0.524 ±  0.069 | 0.674 ±  0.019 | 0.097 |  |  |
| *Exiguobacterium* | 0hr | 2.327 ±  0.114 | 2.327 ±  0.114 | 1.000 | No significant comparisons | No significant comparisons |
|  | 30m | 1.516 ±  1.314 | 1.763 ±  1.505 | 0.842 |  |  |
|  | 2hr | 1.097 ±  0.994 | 2.719 ±  0.065 | 0.116 |  |  |
|  | 8hr | 1.436 ±  0.495 | 2.358 ±  0.267 | 0.146 |  |  |
|  | 24hr | 1.371 ±  1.373 | 2.582 ±  0.142 | 0.340 |  |  |
| *Formosimonas* | 0hr | 0.107 ±  0.004 | 0.107 ±  0.004 | 1.000 | 24hr > 0hr,  p = 0.037 | No significant comparisons |
|  | 30m | 0.081 ±  0.070 | 0.075 ±  0.065 | 0.920 |  |  |
|  | 2hr | 0.061 ±  0.053 | 0.077 ±  0.062 | 0.780 |  |  |
|  | 8hr | 0.086 ±  0.021 | 0.100 ±  0.022 | 0.570 |  |  |
|  | 24hr | 0.091 ±  0.038 | 0.125 ±  0.003 | 0.330 |  |  |
| *Limnobacter* | 0hr | 0.146 ±  0.006 | 0.146 ±  0.006 | 1.000 | 24hr > 2hr,  p = 0.025 | No significant comparisons |
|  | 30m | 0.133 ±  0.023 | 0.107 ±  0.071 | 0.578 |  |  |
|  | 2hr | 0.139 ±  0.049 | 0.142 ±  0.001 | 0.939 |  |  |
|  | 8hr | 0.153 ±  0.012 | 0.153 ±  0.016 | 0.969 |  |  |
|  | 24hr | 0.342 ±  0.252 | 0.153 ±  0.002 | 0.401 |  |  |
| *Paenibacillus* | 0hr | 0.729 ±  0.056 | 0.729 ±  0.056 | 1.000 | No significant comparisons | No significant comparisons |
|  | 30m | 0.483 ±  0.417 | 0.451 ±  0.335 | 0.922 |  |  |
|  | 2hr | 0.416 ±  0.354 | 0.687 ±  0.009 | 0.380 |  |  |
|  | 8hr | 0.601 ±  0.037 | 0.659 ±  0.006 | 0.161 |  |  |
|  | 24hr | 0.583 ±  0.143 | 0.710 ±  0.040 | 0.351 |  |  |
| *Phenylobacterium* | 0hr | 0.490 ±  0.040 | 0.490 ±  0.040 | 1.000 | No significant comparisons | No significant comparisons |
|  | 30m | 0.317 ±  0.270 | 0.340 ±  0.177 | 0.909 |  |  |
|  | 2hr | 0.273 ±  0.215 | 0.475 ±  0.004 | 0.296 |  |  |
|  | 8hr | 0.362 ±  0.132 | 0.461 ±  0.019 | 0.402 |  |  |
|  | 24hr | 0.340 ±  0.148 | 0.484 ±  0.015 | 0.306 |  |  |
| *Polynucleobacter* | 0hr | 0.232 ±  0.025 | 0.232 ±  0.025 | 1.000 | 8hr > 2hr,  p = 0.048 | No significant comparisons |
|  | 30m | 0.161 ±  0.114 | 0.517 ±  0.645 | 0.399 |  |  |
|  | 2hr | 0.172 ±  0.080 | 0.153 ±  0.015 | 0.769 |  |  |
|  | 8hr | 0.180 ±  0.067 | 0.238 ±  0.023 | 0.368 |  |  |
|  | 24hr | 0.232 ±  0.025 | 0.216 ±  0.049 | 0.882 |  |  |
| *Pseudomonas* | 0hr | 0.151 ±  0.015 | 0.151 ±  0.015 | 1.000 | 8hr > 2hr,  p = 0.046 | No significant comparisons |
|  | 30m | 0.152 ±  0.091 | 0.306 ±  0.344 | 0.496 |  |  |
|  | 2hr | 0.121 ±  0.033 | 0.134 ±  0.009 | 0.635 |  |  |
|  | 8hr | 0.106 ±  0.066 | 0.177 ±  0.010 | 0.277 |  |  |
|  | 24hr | 0.152 ±  0.078 | 0.180 ±  0.030 | 0.683 |  |  |
| *Pseudotabrizicola* | 0hr | 0.068 ±  0.004 | 0.068 ±  0.004 | 1.000 | No significant comparisons | No significant comparisons |
|  | 30m | 0.045 ±  0.039 | 0.066 ±  0.015 | 0.444 |  |  |
|  | 2hr | 0.039 ±  0.034 | 0.064 ±  0.001 | 0.407 |  |  |
|  | 8hr | 0.070 ±  0.005 | 0.072 ±  0.003 | 0.727 |  |  |
|  | 24hr | 0.046 ±  0.023 | 0.064 ±  0.002 | 0.392 |  |  |
| *Rhizobium* | 0hr | 0.057 ±  0.001 | 0.057 ±  0.001 | 1.000 | No significant comparisons | No significant comparisons |
|  | 30m | 0.041 ±  0.029 | 0.040 ±  0.034 | 0.946 |  |  |
|  | 2hr | 0.038 ±  0.030 | 0.057 ±  0.001 | 0.444 |  |  |
|  | 8hr | 0.053 ±  0.004 | 0.053 ±  0.008 | 0.965 |  |  |
|  | 24hr | 0.036 ±  0.016 | 0.058 ±  0.006 | 0.224 |  |  |
| *Rhodococcus* | 0hr | 0.931 ±  0.055 | 0.931 ±  0.055 | 1.000 | No significant comparisons | No significant comparisons |
|  | 30m | 0.636 ±  0.548 | 0.804 ±  0.168 | 0.638 |  |  |
|  | 2hr | 0.572 ±  0.358 | 0.944 ±  0.018 | 0.258 |  |  |
|  | 8hr | 0.791 ±  0.186 | 0.958 ±  4.90e-4 | 0.333 |  |  |
|  | 24hr | 1.392 ±  0.632 | 0.994 ±  0.044 | 0.468 |  |  |
| *Sphingobium* | 0hr | 2.875 ±  0.119 | 2.875 ±  0.119 | 1.000 | No significant comparisons | No significant comparisons |
|  | 30m | 1.765 ±  1.502 | 2.563 ±  1.903 | 0.600 |  |  |
|  | 2hr | 1.942 ±  1.648 | 3.356 ±  0.182 | 0.335 |  |  |
|  | 8hr | 2.982 ±  0.207 | 3.062 ±  0.037 | 0.645 |  |  |
|  | 24hr | 2.104 ±  1.242 | 3.330 ±  0.335 | 0.310 |  |  |
| *Stenotrophomonas* | 0hr | 0.611 ±  0.001 | 0.611 ±  0.001 | 1.000 | 0hr > 2hr,  p = 0.007 | No significant comparisons |
|  | 30m | 0.462 ±  0.367 | 0.946 ±  0.587 | 0.292 |  |  |
|  | 2hr | 0.415 ±  0.243 | 0.566 ±  0.005 | 0.463 |  |  |
|  | 8hr | 0.535 ±  0.128 | 0.620 ±  0.058 | 0.483 |  |  |
|  | 24hr | 0.740 ±  0.103 | 0.575 ±  0.012 | 0.152 |  |  |
| *Streptomyces* | 0hr | 0.508 ±  0.008 | 0.508 ±  0.008 | 1.000 | No significant comparisons | No significant comparisons |
|  | 30m | 0.355 ±  0.309 | 0.390 ±  0.275 | 0.892 |  |  |
|  | 2hr | 0.309 ±  0.268 | 0.541 ±  0.012 | 0.331 |  |  |
|  | 8hr | 0.440 ±  0.077 | 0.512 ±  0.013 | 0.320 |  |  |
|  | 24hr | 0.373 ±  0.221 | 0.511 ±  0.024 | 0.474 |  |  |
| **Peak Bloom Alkyl Hydroperoxide Reductase Marker Gene:*rpoB* Expression** | | | | | | |
| **Genus** | **Time** | **Control Ratio** | **H2O2 Ratio** | **T-Test Results (Comparing Treatments)** | **Significant**  **T-Test Results (Comparing H2O2)** | **Significant**  **T-Test Results (Comparing Control)** |
| *Aquirufa* | 0hr | 0.093 ±  0.081 | 0.093 ±  0.081 | 1.000 | 24hr > 2hr,  p = 0.023  24hr > 30min,  p = 0.023 | No significant comparisons |
|  | 30m | 0.047 ±  0.081 | 0.000 ±  0.000 | 0.377 |  |  |
|  | 2hr | 0.000 ±  0.000 | 0.000 ±  0.000 | N/A |  |  |
|  | 8hr | 0.000 ±  0.000 | 0.022 ±  0.038 | 0.374 |  |  |
|  | 24hr | 0.040 ±  0.070 | 0.083 ±  0.040 | 0.409 |  |  |
| *Chitinilyticum* | 0hr | 0.348 ±  0.291 | 0.348 ±  0.291 | 1.000 | 24hr > 0hr,  p = 0.004  8hr > 0hr,  p = 0.004  24hr > 2hr,  p = 0.006  24hr > 30min,  p = 0.022  8hr > 2hr,  p = 0.005  8hr > 30min,  p = 0.016 | No significant comparisons |
|  | 30m | 0.237 ±  0.395 | 0.743 ±  0.526 | 0.254 |  |  |
|  | 2hr | 0.631 ±  0.483 | 0.619 ±  0.228 | 0.971 |  |  |
|  | 8hr | 0.076 ±  0.033 | 2.456 ±  0.525 | 0.001 ** |  |  |
|  | 24hr | 0.208 ±  0.248 | 2.235 ±  0.471 | 0.003 ** |  |  |
| *Chitinimonas* | 0hr | 0.009 ±  0.005 | 0.009 ±  0.005 | 1.000 | No significant comparisons | 0hr > 8hr,  p = 0.040 |
|  | 30m | 0.004 ±  0.008 | 0.005 ±  0.007 | 0.902 |  |  |
|  | 2hr | 0.010 ±  0.014 | 0.005 ±  0.009 | 0.674 |  |  |
|  | 8hr | 0.001 ±  0.001 | 0.025 ±  0.041 | 0.360 |  |  |
|  | 24hr | 0.004 ±  0.007 | 0.015 ±  0.007 | 0.128 |  |  |
| *Cupriavidus* | 0hr | 0.120 ±  0.097 | 0.120 ±  0.097 | 1.000 | No significant comparisons | No significant comparisons |
|  | 30m | 0.075 ±  0.090 | 0.000 ±  0.000 | 0.221 |  |  |
|  | 2hr | 0.021 ±  0.019 | 0.000 ±  0.000 | 0.131 |  |  |
|  | 8hr | 0.014 ±  0.023 | 0.086 ±  0.150 | 0.453 |  |  |
|  | 24hr | 0.062 ±  0.108 | 0.039 ±  0.032 | 0.734 |  |  |
| *Defluviimonas* | 0hr | 0.072 ±  0.044 | 0.072 ±  0.044 | 1.000 | No significant comparisons | No significant comparisons |
|  | 30m | 0.037 ±  0.049 | 0.013 ±  0.023 | 0.492 |  |  |
|  | 2hr | 0.023 ±  0.018 | 0.450 ±  0.741 | 0.375 |  |  |
|  | 8hr | 0.006 ±  0.009 | 0.007 ±  0.013 | 0.855 |  |  |
|  | 24hr | 0.044 ±  0.053 | 0.029 ±  0.026 | 0.697 |  |  |
| *Deinococcus* | 0hr | 0.160 ±  0.139 | 0.160 ±  0.139 | 1.000 | 24hr > 2hr,  p = 0.036  24hr > 30min,  p = 0.036 | No significant comparisons |
|  | 30m | 0.080 ±  0.138 | 0.000 ±  0.000 | 0.373 |  |  |
|  | 2hr | 0.001 ±  0.002 | 0.000 ±  0.000 | 0.374 |  |  |
|  | 8hr | 0.000 ±  0.000 | 0.024 ±  0.042 | 0.374 |  |  |
|  | 24hr | 0.078 ±  0.136 | 0.055 ±  0.031 | 0.788 |  |  |
| *Enterobacter* | 0hr | 0.110 ±  0.095 | 0.110 ±  0.095 | 1.000 | 24hr > 0hr,  p = 0.010  8hr > 0hr,  p = 0.041  24hr > 2hr,  p = 0.020  24hr > 30min,  p = 0.012 | No significant comparisons |
|  | 30m | 0.130 ±  0.183 | 0.222 ±  0.131 | 0.516 |  |  |
|  | 2hr | 0.192 ±  0.210 | 0.362 ±  0.253 | 0.422 |  |  |
|  | 8hr | 0.025 ±  0.022 | 1.154 ±  0.598 | 0.031 |  |  |
|  | 24hr | 0.052 ±  0.079 | 1.843 ±  0.635 | 0.008 * |  |  |
| *Escherichia* | 0hr | 0.445 ±  0.387 | 0.445 ±  0.387 | 1.000 | 24hr > 0hr,  p = 0.044  24hr > 30min,  p = 0.044  24hr > 8hr,  p = 0.004 | No significant comparisons |
|  | 30m | 0.184 ±  0.316 | 0.085 ±  0.051 | 0.622 |  |  |
|  | 2hr | 0.135 ±  0.167 | 0.805 ±  0.794 | 0.226 |  |  |
|  | 8hr | 0.015 ±  0.015 | 0.364 ±  0.181 | 0.029 |  |  |
|  | 24hr | 0.220 ±  0.381 | 1.124 ±  0.120 | 0.017 * |  |  |
| *Exiguobacterium* | 0hr | 1.990 ±  1.728 | 1.990 ±  1.728 | 1.000 | 24hr > 2hr,  p = 0.003  24hr > 30min,  p = 0.003  24hr > 8hr,  p = 0.044 | No significant comparisons |
|  | 30m | 1.079 ±  1.870 | 0.059 ±  0.065 | 0.398 |  |  |
|  | 2hr | 0.072 ±  0.124 | 0.031 ±  0.039 | 0.615 |  |  |
|  | 8hr | 0.001 ±  0.001 | 0.310 ±  0.322 | 0.171 |  |  |
|  | 24hr | 0.899 ±  1.557 | 0.980 ±  0.240 | 0.933 |  |  |
| *Formosimonas* | 0hr | 0.087 ±  0.076 | 0.087 ±  0.076 | 1.000 | No significant comparisons | No significant comparisons |
|  | 30m | 0.039 ±  0.067 | 0.000 ±  0.000 | 0.374 |  |  |
|  | 2hr | 0.033 ±  0.043 | 0.000 ±  0.000 | 0.260 |  |  |
|  | 8hr | 0.000 ±  0.000 | 0.017 ±  0.024 | 0.272 |  |  |
|  | 24hr | 0.033 ±  0.057 | 0.027 ±  0.017 | 0.864 |  |  |
| *Limnobacter* | 0hr | 0.096 ±  0.084 | 0.096 ±  0.084 | 1.000 | 24hr > 0hr,  p = 0.019  24hr > 2hr,  p = 0.019  24hr > 30min,  p = 0.024 | No significant comparisons |
|  | 30m | 0.052 ±  0.083 | 0.183 ±  0.221 | 0.390 |  |  |
|  | 2hr | 0.142 ±  0.160 | 0.097 ±  0.084 | 0.691 |  |  |
|  | 8hr | 0.020 ±  0.018 | 1.498 ±  1.960 | 0.262 |  |  |
|  | 24hr | 0.045 ±  0.077 | 2.145 ±  0.932 | 0.018 * |  |  |
| *Paenibacillus* | 0hr | 0.460 ±  0.398 | 0.460 ±  0.398 | 1.000 | 24hr > 2hr,  p = 0.002  24hr > 30min,  p = 0.006  8hr > 2hr,  p = 0.042 | No significant comparisons |
|  | 30m | 0.249 ±  0.431 | 0.125 ±  0.121 | 0.657 |  |  |
|  | 2hr | 0.076 ±  0.085 | 0.057 ±  0.099 | 0.811 |  |  |
|  | 8hr | 0.002 ±  0.001 | 0.326 ±  0.123 | 0.010 * |  |  |
|  | 24hr | 0.211 ±  0.350 | 0.548 ±  0.071 | 0.178 |  |  |
| *Phenylobacterium* | 0hr | 0.308 ±  0.267 | 0.308 ±  0.267 | 1.000 | 24hr > 2hr,  p = 0.024  24hr > 30min,  p = 0.019 | No significant comparisons |
|  | 30m | 0.179 ±  0.310 | 0.034 ±  0.031 | 0.466 |  |  |
|  | 2hr | 0.058 ±  0.055 | 0.039 ±  0.050 | 0.681 |  |  |
|  | 8hr | 0.001 ±  0.001 | 0.122 ±  0.119 | 0.153 |  |  |
|  | 24hr | 0.155 ±  0.268 | 0.285 ±  0.109 | 0.478 |  |  |
| *Polynucleobacter* | 0hr | 0.080 ±  0.061 | 0.080 ±  0.061 | 1.000 | 24hr > 0hr,  p = 0.005  24hr > 2hr,  p = 0.000673  24hr > 8hr,  p = 0.015 | No significant comparisons |
|  | 30m | 0.071 ±  0.116 | 0.198 ±  0.191 | 0.382 |  |  |
|  | 2hr | 0.051 ±  0.041 | 0.001 ±  0.002 | 0.101 |  |  |
|  | 8hr | 0.001 ±  0.002 | 0.089 ±  0.101 | 0.206 |  |  |
|  | 24hr | 0.034 ±  0.056 | 0.380 ±  0.069 | 0.002 ** |  |  |
| *Pseudomonas* | 0hr | 0.085 ±  0.060 | 0.085 ±  0.060 | 1.000 | 24hr > 0hr,  p = 0.031 | 0hr > 2hr,  p = 0.029  24hr > 2hr,  p = 0.013  2hr > 30min,  p = 0.036  2hr > 8hr,  p = 0.024 |
|  | 30m | 0.077 ±  0.098 | 0.338 ±  0.237 | 0.153 |  |  |
|  | 2hr | 0.309 ±  0.100 | 1.448 ±  1.047 | 0.134 |  |  |
|  | 8hr | 0.069 ±  0.061 | 0.816 ±  0.926 | 0.236 |  |  |
|  | 24hr | 0.050 ±  0.034 | 0.580 ±  0.257 | 0.024 * |  |  |
| *Pseudotabrizicola* | 0hr | 0.081 ±  0.068 | 0.081 ±  0.068 | 1.000 | No significant comparisons | No significant comparisons |
|  | 30m | 0.036 ±  0.063 | 0.000 ±  0.000 | 0.375 |  |  |
|  | 2hr | 0.005 ±  0.007 | 0.000 ±  0.000 | 0.302 |  |  |
|  | 8hr | 0.001 ±  0.001 | 0.010 ±  0.018 | 0.379 |  |  |
|  | 24hr | 0.081 ±  0.068 | 0.031 ±  0.039 | 0.960 |  |  |
| *Rhizobium* | 0hr | 0.052 ±  0.023 | 0.052 ±  0.023 | 1.000 | No significant comparisons | 0hr > 2hr,  p = 0.029  0hr > 8hr,  p = 0.036 |
|  | 30m | 0.019 ±  0.033 | 0.019 ±  0.032 | 0.989 |  |  |
|  | 2hr | 0.007 ±  0.006 | 0.066 ±  0.075 | 0.247 |  |  |
|  | 8hr | 0.009 ±  0.008 | 0.065 ±  0.064 | 0.206 |  |  |
|  | 24hr | 0.019 ±  0.031 | 0.020 ±  0.007 | 0.968 |  |  |
| *Rhodococcus* | 0hr | 0.590 ±  0.498 | 0.590 ±  0.498 | 1.000 | No significant comparisons | No significant comparisons |
|  | 30m | 0.364 ±  0.580 | 0.629 ±  0.368 | 0.542 |  |  |
|  | 2hr | 0.672 ±  0.590 | 1.826 ±  1.820 | 0.355 |  |  |
|  | 8hr | 0.098 ±  0.025 | 6.605 ±  9.308 | 0.293 |  |  |
|  | 24hr | 0.412 ±  0.0442 | 1.971 ±  1.818 | 0.222 |  |  |
| *Sphingobium* | 0hr | 1.659 ±  1.436 | 1.659 ±  1.436 | 1.000 | No significant comparisons | No significant comparisons |
|  | 30m | 0.751 ±  1.250 | 0.253 ±  0.033 | 0.528 |  |  |
|  | 2hr | 0.299 ±  0.331 | 0.866 ±  0.624 | 0.237 |  |  |
|  | 8hr | 0.030 ±  0.013 | 2.204 ±  2.346 | 0.184 |  |  |
|  | 24hr | 0.893 ±  1.495 | 1.316 ±  1.039 | 0.708 |  |  |
| *Stenotrophomonas* | 0hr | 0.414 ±  0.343 | 0.414 ±  0.343 | 1.000 | No significant comparisons | No significant comparisons |
|  | 30m | 0.339 ±  0.543 | 0.522 ±  0.230 | 0.619 |  |  |
|  | 2hr | 0.434 ±  0.246 | 1.242 ±  1.059 | 0.268 |  |  |
|  | 8hr | 0.084 ±  0.071 | 1.632 ±  0.683 | 0.018 * |  |  |
|  | 24hr | 0.240 ±  0.343 | 0.877 ±  0.163 | 0.041 * |  |  |
| *Streptomyces* | 0hr | 0.402 ±  0.334 | 0.402 ±  0.334 | 1.000 | 24hr > 2hr,  p = 0.018 | No significant comparisons |
|  | 30m | 0.226 ±  0.386 | 0.050 ±  0.065 | 0.480 |  |  |
|  | 2hr | 0.035 ±  0.024 | 0.000 ±  0.000 | 0.068 |  |  |
|  | 8hr | 0.008 ±  0.008 | 0.101 ±  0.083 | 0.127 |  |  |
|  | 24hr | 0.221 ±  0.320 | 0.204 ±  0.091 | 0.932 |  |  |
| **Bloom Decline Alkyl Hydroperoxide Reductase Marker Genes:*rpoB* Expression** | | | | | | |
| **Genus** | **Time** | **Control Ratio** | **H2O2 Ratio** | **T-Test Results (Comparing Treatments)** | **Significant**  **T-Test Results (Comparing H2O2)** | **Significant**  **T-Test Results (Comparing Control)** |
| *Aquirufa* | 0hr | 0.140 ±  0.021 | 0.140 ±  0.021 | 1.000 | No significant comparisons | No significant comparisons |
|  | 30m | 0.122 ±  0.018 | 0.140 ±  0.022 | 0.404 |  |  |
|  | 2hr | 0.140 ±  0.005 | 0.146 ±  0.017 | 0.604 |  |  |
|  | 24hr | 0.144 ±  0.011 | 0.142 ±  0.016 | 0.839 |  |  |
| *Chitinilyticum* | 0hr | 0.539 ±  0.114 | 0.539 ±  0.114 | 1.000 | No significant comparisons | No significant comparisons |
|  | 30m | 0.503 ±  0.034 | 0.553 ±  0.016 | 0.160 |  |  |
|  | 2hr | 0.566 ±  0.032 | 0.589 ±  0.043 | 0.489 |  |  |
|  | 24hr | 0.519 ±  0.068 | 0.606 ±  0.055 | 0.161 |  |  |
| *Chitinimonas* | 0hr | 0.013 ±  0.001 | 0.013 ±  0.001 | 1.000 | No significant comparisons | No significant comparisons |
|  | 30m | 0.014 ±  0.001 | 0.013 ±  0.001 | 0.169 |  |  |
|  | 2hr | 0.013 ±  0.001 | 0.015 ±  0.001 | 0.086 |  |  |
|  | 24hr | 0.014 ±  0.001 | 0.013 ±  0.002 | 0.547 |  |  |
| *Cupriavidus* | 0hr | 0.204 ±  0.024 | 0.204 ±  0.024 | 1.000 | No significant comparisons | 2hr > 24hr,  p = 0.047  30min > 24hr,  p = 0.042 |
|  | 30m | 0.205 ±  0.010 | 0.211 ±  0.021 | 0.703 |  |  |
|  | 2hr | 0.198 ±  0.001 | 0.196 ±  0.014 | 0.869 |  |  |
|  | 24hr | 0.176 ±  0.013 | 0.177 ±  0.018 | 0.930 |  |  |
| *Defluviimonas* | 0hr | 0.095 ±  0.020 | 0.095 ±  0.020 | 1.000 | No significant comparisons | No significant comparisons |
|  | 30m | 0.110 ±  0.008 | 0.100 ±  0.010 | 0.293 |  |  |
|  | 2hr | 0.097 ±  0.010 | 0.102 ±  0.003 | 0.476 |  |  |
|  | 24hr | 0.099 ±  0.007 | 0.100 ±  0.001 | 0.751 |  |  |
| *Deinococcus* | 0hr | 0.232 ±  0.001 | 0.232 ±  0.001 | 1.000 | No significant comparisons | No significant comparisons |
|  | 30m | 0.230 ±  0.006 | 0.229 ±  0.002 | 0.857 |  |  |
|  | 2hr | 0.242 ±  0.008 | 0.323 ±  0.145 | 0.386 |  |  |
|  | 24hr | 0.231 ±  0.001 | 0.229 ±  0.007 | 0.555 |  |  |
| *Enterobacter* | 0hr | 0.170 ±  0.008 | 0.170 ±  0.008 | 1.000 | 24hr > 0hr,  p = 0.049 | 2hr > 30min,  p = 0.047 |
|  | 30m | 0.162 ±  0.003 | 0.171 ±  0.046 | 0.730 |  |  |
|  | 2hr | 0.193 ±  0.014 | 0.182 ±  0.022 | 0.496 |  |  |
|  | 24hr | 0.231 ±  0.094 | 0.217 ±  0.019 | 0.818 |  |  |
| *Escherichia* | 0hr | 0.676 ±  0.012 | 0.676 ±  0.012 | 1.000 | No significant comparisons | No significant comparisons |
|  | 30m | 0.680 ±  0.095 | 0.756 ±  0.194 | 0.585 |  |  |
|  | 2hr | 0.587 ±  0.050 | 0.635 ±  0.062 | 0.356 |  |  |
|  | 24hr | 0.644 ±  0.060 | 0.582 ±  0.052 | 0.251 |  |  |
| *Exiguobacterium* | 0hr | 2.612 ±  0.589 | 2.612 ±  0.589 | 1.000 | No significant comparisons | No significant comparisons |
|  | 30m | 2.187 ±  0.627 | 2.440 ±  1.406 | 0.791 |  |  |
|  | 2hr | 2.812 ±  0.046 | 3.219 ±  0.267 | 0.060 |  |  |
|  | 24hr | 2.457 ±  0.620 | 3.628 ±  0.136 | 0.033 * |  |  |
| *Formosimonas* | 0hr | 0.116 ±  0.015 | 0.116 ±  0.015 | 1.000 | No significant comparisons | No significant comparisons |
|  | 30m | 0.115 ±  0.034 | 0.108 ±  0.013 | 0.821 |  |  |
|  | 2hr | 0.116 ±  0.003 | 0.108 ±  0.008 | 0.151 |  |  |
|  | 24hr | 0.111 ±  0.025 | 0.108 ±  0.011 | 0.848 |  |  |
| *Limnobacter* | 0hr | 0.162 ±  0.004 | 0.162 ±  0.004 | 1.000 | No significant comparisons | No significant comparisons |
|  | 30m | 0.150 ±  0.013 | 0.184 ±  0.054 | 0.338 |  |  |
|  | 2hr | 0.155 ±  0.007 | 0.149 ±  0.008 | 0.361 |  |  |
|  | 24hr | 0.150 ±  0.008 | 0.146 ±  0.014 | 0.708 |  |  |
| *Paenibacillus* | 0hr | 0.679 ±  0.001 | 0.679 ±  0.001 | 1.000 | 24hr > 0hr,  p = 0.034  24hr > 30min,  p = 0.049 | No significant comparisons |
|  | 30m | 0.674 ±  0.067 | 0.662 ±  0.032 | 0.841 |  |  |
|  | 2hr | 0.679 ±  0.016 | 0.710 ±  0.029 | 0.175 |  |  |
|  | 24hr | 0.728 ±  0.100 | 0.734 ±  0.020 | 0.921 |  |  |
| *Phenylobacterium* | 0hr | 0.477 ±  0.014 | 0.477 ±  0.014 | 1.000 | No significant comparisons | No significant comparisons |
|  | 30m | 0.436 ±  0.059 | 0.451 ±  0.134 | 0.865 |  |  |
|  | 2hr | 0.495 ±  0.003 | 0.586 ±  0.149 | 0.812 |  |  |
|  | 24hr | 0.496 ±  0.022 | 0.534 ±  0.043 | 0.244 |  |  |
| *Polynucleobacter* | 0hr | 0.118 ±  0.009 | 0.118 ±  0.009 | 1.000 | 24hr > 0hr,  p = 0.023  24hr > 2hr,  p = 0.048 | 2hr > 30min,  p = 0.047 |
|  | 30m | 0.117 ±  0.003 | 0.118 ±  0.029 | 0.986 |  |  |
|  | 2hr | 0.139 ±  0.013 | 0.121 ±  0.016 | 0.199 |  |  |
|  | 24hr | 0.138 ±  0.016 | 0.150 ±  0.008 | 0.298 |  |  |
| *Pseudomonas* | 0hr | 0.114 ±  0.026 | 0.114 ±  0.026 | 1.000 | No significant comparisons | No significant comparisons |
|  | 30m | 0.099 ±  0.037 | 0.100 ±  0.055 | 0.974 |  |  |
|  | 2hr | 0.127 ±  0.017 | 0.135 ±  0.012 | 0.539 |  |  |
|  | 24hr | 0.110 ±  0.030 | 0.150 ±  0.010 | 0.098 |  |  |
| *Pseudotabrizicola* | 0hr | 0.110 ±  0.010 | 0.110 ±  0.010 | 1.000 | 24hr > 30min,  p = 0.049 | No significant comparisons |
|  | 30m | 0.116 ±  0.004 | 0.100 ±  0.004 | 0.026 * |  |  |
|  | 2hr | 0.113 ±  0.003 | 0.109 ±  0.015 | 0.659 |  |  |
|  | 24hr | 0.110 ±  0.010 | 0.112 ±  0.004 | 0.341 |  |  |
| *Rhizobium* | 0hr | 0.059 ±  0.003 | 0.059 ±  0.003 | 1.000 | No significant comparisons | No significant comparisons |
|  | 30m | 0.060 ±  0.004 | 0.055 ±  0.009 | 0.497 |  |  |
|  | 2hr | 0.064 ±  0.006 | 0.064 ±  0.013 | 1.000 |  |  |
|  | 24hr | 0.060 ±  0.004 | 0.065 ±  0.007 | 0.318 |  |  |
| *Rhodococcus* | 0hr | 0.918 ±  0.063 | 0.918 ±  0.063 | 1.000 | No significant comparisons | No significant comparisons |
|  | 30m | 0.820 ±  0.073 | 0.974 ±  0.002 | 0.065 |  |  |
|  | 2hr | 0.901 ±  0.027 | 0.958 ±  0.018 | 0.037 * |  |  |
|  | 24hr | 0.907 ±  0.101 | 0.967 ±  0.028 | 0.387 |  |  |
| *Sphingobium* | 0hr | 2.411 ±  0.014 | 2.411 ±  0.014 | 1.000 | No significant comparisons | No significant comparisons |
|  | 30m | 2.548 ±  0.119 | 2.596 ±  0.238 | 0.771 |  |  |
|  | 2hr | 2.550 ±  0.109 | 2.431 ±  0.207 | 0.427 |  |  |
|  | 24hr | 2.384 ±  0.049 | 2.264 ±  0.115 | 0.169 |  |  |
| *Stenotrophomonas* | 0hr | 0.664 ±  0.073 | 0.664 ±  0.073 | 1.000 | No significant comparisons | No significant comparisons |
|  | 30m | 0.668 ±  0.055 | 0.653 ±  0.119 | 0.856 |  |  |
|  | 2hr | 0.686 ±  0.019 | 0.718 ±  0.070 | 0.498 |  |  |
|  | 24hr | 0.665 ±  0.032 | 0.751 ±  0.071 | 0.131 |  |  |
| *Streptomyces* | 0hr | 0.645 ±  0.000 | 0.645 ±  0.000 | 1.000 | No significant comparisons | No significant comparisons |
|  | 30m | 0.580 ±  0.029 | 0.585 ±  0.102 | 0.923 |  |  |
|  | 2hr | 0.614 ±  0.022 | 0.648 ±  0.020 | 0.120 |  |  |
|  | 24hr | 0.627 ±  0.042 | 0.507 ±  0.220 | 0.408 |  |  |

# Supplemental References

[Asker, D., Awad, T. S., Beppu, T., & Ueda, K. (2008). *Deinococcus misasensis* and *Deinococcus roseus*, novel members of the genus *Deinococcus*, isolated from a radioactive site in Japan. *Systematic and Applied Microbiology*, *31*(1), 43–49. https://doi.org/10.1016/j.syapm.2007.10.002](https://www.zotero.org/google-docs/?BrCTHq)

[Baek, C., Shin, S.-K., & Yi, H. (2019). Phenylobacterium parvum sp. Nov., isolated from lake water. *International Journal of Systematic and Evolutionary Microbiology*, *69*(4), 1169–1172. https://doi.org/10.1099/ijsem.0.003284](https://www.zotero.org/google-docs/?BrCTHq)

[Baek, K., Chung, E. J., Choi, G.-G., Kim, M.-K., Lim, S., & Choi, A. (2018). Deinococcus koreensis sp. Nov., a gamma radiation-resistant bacterium isolated from river water. *International Journal of Systematic and Evolutionary Microbiology*, *68*(8), 2545–2550. https://doi.org/10.1099/ijsem.0.002872](https://www.zotero.org/google-docs/?BrCTHq)

[Baik, K. S., Lim, C. H., Choe, H. N., Kim, E. M., & Seong, C. N. (2011). Paenibacillus rigui sp. Nov., isolated from a freshwater wetland. *International Journal of Systematic and Evolutionary Microbiology*, *61*(3), 529–534. https://doi.org/10.1099/ijs.0.021485-0](https://www.zotero.org/google-docs/?BrCTHq)

[Chang, S.-C., Wu, M.-C., Chen, W.-M., Tsai, Y.-H., & Lee, T.-M. (2009). Chitinilyticum litopenaei sp. Nov., isolated from a freshwater shrimp pond, and emended description of the genus Chitinilyticum. *International Journal of Systematic and Evolutionary Microbiology*, *59*(11), 2651–2655. https://doi.org/10.1099/ijs.0.005090-0](https://www.zotero.org/google-docs/?BrCTHq)

[Chauhan, D., Srivastava, P. A., Yennamalli, R. M., & Priyadarshini, R. (2017). Draft Genome Sequence of Deinococcus indicus DR1, a Novel Strain Isolated from a Freshwater Wetland. *Genome Announcements*, *5*(31), 10.1128/genomea.00754-17. https://doi.org/10.1128/genomea.00754-17](https://www.zotero.org/google-docs/?BrCTHq)

[Chen, H., Jogler, M., Rohde, M., Klenk, H.-P., Busse, H.-J., Tindall, B. J., Spröer, C., & Overmann, J. (2013). Sphingobium limneticum sp. Nov. And Sphingobium boeckii sp. Nov., two freshwater planktonic members of the family Sphingomonadaceae, and reclassification of Sphingomonas suberifaciens as Sphingobium suberifaciens comb. Nov. *International Journal of Systematic and Evolutionary Microbiology*, *63*(Pt_2), 735–743. https://doi.org/10.1099/ijs.0.040105-0](https://www.zotero.org/google-docs/?BrCTHq)

[Chen, W.-M., Guo, Y.-P., Sheu, C., & Sheu, S.-Y. (2020). Sphingobium algorifonticola sp. Nov., isolated from a cold spring. *International Journal of Systematic and Evolutionary Microbiology*, *70*(1), 309–316. https://doi.org/10.1099/ijsem.0.003755](https://www.zotero.org/google-docs/?BrCTHq)

[Chen, W.-M., Xie, P.-B., Young, C.-C., & Sheu, S.-Y. (2017). Formosimonas limnophila gen. Nov., sp. Nov., a new member of the family Burkholderiaceae isolated from a freshwater lake. *International Journal of Systematic and Evolutionary Microbiology*, *67*(1), 17–24. https://doi.org/10.1099/ijsem.0.001561](https://www.zotero.org/google-docs/?BrCTHq)

[Chen, W.-M., Yang, C.-C., Young, C.-C., Lin, S.-Y., & Sheu, S.-Y. (2021). *Rhizobium lacunae sp. Nov., Isolated From a Freshwater Pond*. Research Square. https://doi.org/10.21203/rs.3.rs-1145264/v1](https://www.zotero.org/google-docs/?BrCTHq)

[Colomer-Lluch, M., Mora, A., López, C., Mamani, R., Dahbi, G., Marzoa, J., Herrera, A., Viso, S., Blanco, J. E., Blanco, M., Alonso, M. P., Jofre, J., Muniesa, M., & Blanco, J. (2013). Detection of quinolone-resistant Escherichia coli isolates belonging to clonal groups O25b:H4-B2-ST131 and O25b:H4-D-ST69 in raw sewage and river water in Barcelona, Spain. *Journal of Antimicrobial Chemotherapy*, *68*(4), 758–765. https://doi.org/10.1093/jac/dks477](https://www.zotero.org/google-docs/?BrCTHq)

[Deng, Y., Han, X.-F., Jiang, Z.-M., Yu, L.-Y., Li, Y., & Zhang, Y.-Q. (2022). Characterization of three Stenotrophomonas strains isolated from different ecosystems and proposal of Stenotrophomonas mori sp. Nov. And Stenotrophomonas lacuserhaii sp. Nov. *Frontiers in Microbiology*, *13*. https://doi.org/10.3389/fmicb.2022.1056762](https://www.zotero.org/google-docs/?BrCTHq)

[Friedrich, I., Hollensteiner, J., Scherf, J., Weyergraf, J., Klassen, A., Poehlein, A., Hertel, R., & Daniel, R. (2021). Complete Genome Sequence of Stenotrophomonas indicatrix DAIF1. *Microbiology Resource Announcements*, *10*(6), 10.1128/mra.01484-20. https://doi.org/10.1128/mra.01484-20](https://www.zotero.org/google-docs/?BrCTHq)

[Hahn, M. W., Karbon, G., Koll, U., Schmidt, J., & Lang, E. (2017). Polynucleobacter sphagniphilus sp. Nov. A planktonic freshwater bacterium isolated from an acidic and humic freshwater habitat. *International Journal of Systematic and Evolutionary Microbiology*, *67*(9), 3261–3267. https://doi.org/10.1099/ijsem.0.002096](https://www.zotero.org/google-docs/?BrCTHq)

[Hahn, M. W., Lang, E., Brandt, U., & Spröer, C. (2011). Polynucleobacter acidiphobus sp. Nov., a representative of an abundant group of planktonic freshwater bacteria. *International Journal of Systematic and Evolutionary Microbiology*, *61*(4), 788–794. https://doi.org/10.1099/ijs.0.023929-0](https://www.zotero.org/google-docs/?BrCTHq)

[Hoke, A. K., Reynoso, G., Smith, M. R., Gardner, M. I., Lockwood, D. J., Gilbert, N. E., Wilhelm, S. W., Becker, I. R., Brennan, G. J., Crider, K. E., Farnan, S. R., Mendoza, V., Poole, A. C., Zimmerman, Z. P., Utz, L. K., Wurch, L. L., & Steffen, M. M. (2021). Genomic signatures of Lake Erie bacteria suggest interaction in the Microcystis phycosphere. *PLOS ONE*, *16*(9), e0257017.](https://www.zotero.org/google-docs/?BrCTHq) <https://doi.org/10.1371/journal.pone.0257017>

Hyun Mi Jin, Hye Kyeong Kang, Sang-Soo Han, Ji Young Jung, Byung-Gon Ryu, & Eu Jin Chung (2019-04-17). Characterization of Novel Mono-aromatic Compounds Degrading Bacteirum, Rhodococcus sp. Strain ABRD24, Using Genomic and Metabolic Analysis. 한국미생물학회 학술대회논문집, 제주.

[Im, W.-T., Jung, H.-M., Ten, L. N., Kim, M. K., Bora, N., Goodfellow, M., Lim, S., Jung, J., & Lee, S.-T. (2008). Deinococcus aquaticus sp. Nov., isolated from fresh water, and Deinococcus caeni sp. Nov., isolated from activated sludge. *International Journal of Systematic and Evolutionary Microbiology*, *58*(10), 2348–2353. https://doi.org/10.1099/ijs.0.64082-0](https://www.zotero.org/google-docs/?BrCTHq)

[Jude, B. A. (2019). Draft Genome Sequence of a Chitinimonas Species from Hudson Valley Waterways That Expresses Violacein Pigment. *Microbiology Resource Announcements*, *8*(35), 10.1128/mra.00683-19. https://doi.org/10.1128/mra.00683-19](https://www.zotero.org/google-docs/?BrCTHq)

[Jung, Y.-T., Park, S., Lee, J.-S., & Yoon, J.-H. (2014). Defluviimonas aquaemixtae sp. Nov., isolated from the junction between a freshwater spring and the ocean. *International Journal of Systematic and Evolutionary Microbiology*, *64*(Pt_12), 4191–4197. https://doi.org/10.1099/ijs.0.068767-0](https://www.zotero.org/google-docs/?BrCTHq)

[Kämpfer, P., Busse, H.-J., McInroy, J. A., Clermont, D., Criscuolo, A., & Glaeser, S. P. (2021). Paenibacillus allorhizosphaerae sp. Nov., from soil of the rhizosphere of Zea mays. *International Journal of Systematic and Evolutionary Microbiology*, *71*(10), 005051. https://doi.org/10.1099/ijsem.0.005051](https://www.zotero.org/google-docs/?BrCTHq)

[Kurth, C., Wasmuth, I., Wichard, T., Pohnert, G., & Nett, M. (2019). Algae induce siderophore biosynthesis in the freshwater bacterium Cupriavidus necator H16. *BioMetals*, *32*(1), 77–88. https://doi.org/10.1007/s10534-018-0159-6](https://www.zotero.org/google-docs/?BrCTHq)

[Le, V. V., Ko, S.-R., Kim, M.-S., Kang, M., Jeong, S., & Ahn, C.-Y. (2024). Sphingobium cyanobacteriorum sp. Nov., isolated from fresh water. *International Journal of Systematic and Evolutionary Microbiology*, *74*(4), 006339. https://doi.org/10.1099/ijsem.0.006339](https://www.zotero.org/google-docs/?BrCTHq)

[Leister, C., & Hügler, M. (2022). Genome Analysis of Enterobacter asburiae and Lelliottia spp. Proliferating in Oligotrophic Drinking Water Reservoirs and Lakes. *Applied and Environmental Microbiology*, *88*(14), e00471-22. https://doi.org/10.1128/aem.00471-22](https://www.zotero.org/google-docs/?BrCTHq)

[Li, X., Blom, J., & Zeng, Y. (2015). Whole genome sequences of a free-living *Pseudomonas* sp. Strain ML96 isolated from a freshwater Maar Lake. *Marine Genomics*, *24*, 219–221. https://doi.org/10.1016/j.margen.2015.05.016](https://www.zotero.org/google-docs/?BrCTHq)

[Ma, T., Xue, H., Piao, C., Liu, C., Yang, M., Bian, D., & Li, Y. (2022). Reclassification of 11 Members of the Family Rhodobacteraceae at Genus and Species Levels and Proposal of Pseudogemmobacter hezensis sp. Nov. *Frontiers in Microbiology*, *13*. https://doi.org/10.3389/fmicb.2022.849695](https://www.zotero.org/google-docs/?BrCTHq)

[Máthé, I., Tóth, E., Mentes, A., Szabó, A., Márialigeti, K., Schumann, P., & Felföldi, T. (2018). A new Rhizobium species isolated from the water of a crater lake, description of Rhizobium aquaticum sp. Nov. *Antonie van Leeuwenhoek*, *111*(11), 2175–2183. https://doi.org/10.1007/s10482-018-1110-0](https://www.zotero.org/google-docs/?BrCTHq)

[Mehdiratta, K., Nain, S., Sharma, M., Singh, S., Srivastava, S., Dhamale, B. D., Mohanty, D., Kamat, S. S., Natarajan, V. T., Sharma, R., & Gokhale, R. S. (2022). Respiratory Quinone Switches from Menaquinone to Polyketide Quinone during the Development Cycle in Streptomyces sp. Strain MNU77. *Microbiology Spectrum*, *11*(1), e02597-22. https://doi.org/10.1128/spectrum.02597-22](https://www.zotero.org/google-docs/?BrCTHq)

[Nicklasson, M., Martín-Rodríguez, A. J., Thorell, K., Higdon, S. M., Neves, L., Mussagy, A., Rydberg, H. A., Hernroth, B., Svensson-Stadler, L., & Sjöling, Å. (2022). Pseudomonas boanensis sp. Nov., a bacterium isolated from river water used for household purposes in Boane District, Mozambique. *International Journal of Systematic and Evolutionary Microbiology*, *72*(7), 005461. https://doi.org/10.1099/ijsem.0.005461](https://www.zotero.org/google-docs/?BrCTHq)

[Ōmura, S., Ikeda, H., Ishikawa, J., Hanamoto, A., Takahashi, C., Shinose, M., Takahashi, Y., Horikawa, H., Nakazawa, H., Osonoe, T., Kikuchi, H., Shiba, T., Sakaki, Y., & Hattori, M. (2001). Genome sequence of an industrial microorganism Streptomyces avermitilis: Deducing the ability of producing secondary metabolites. *Proceedings of the National Academy of Sciences*, *98*(21), 12215–12220. https://doi.org/10.1073/pnas.211433198](https://www.zotero.org/google-docs/?BrCTHq)

[Park, S., Jung, Y.-T., Park, J.-M., & Yoon, J.-H. (2017). Puniceibacterium confluentis sp. Nov., isolated from the junction between the ocean and a freshwater spring, and emended description of the genus Puniceibacterium. *International Journal of Systematic and Evolutionary Microbiology*, *67*(9), 3480–3484. https://doi.org/10.1099/ijsem.0.002145](https://www.zotero.org/google-docs/?BrCTHq)

[Phurbu, D., Wang, H., Tang, Q., Lu, H., Zhu, H., Jiang, S., Xing, P., & Wu, Q. L. (2019). Tabrizicola alkalilacus sp. Nov., isolated from alkaline Lake Dajiaco on the Tibetan Plateau. *International Journal of Systematic and Evolutionary Microbiology*, *69*(11), 3420–3425. https://doi.org/10.1099/ijsem.0.003635](https://www.zotero.org/google-docs/?BrCTHq)

[Pitt, A., Schmidt, J., Koll, U., & Hahn, M. W. (2019). Aquirufa antheringensis gen. Nov., sp. Nov. And Aquirufa nivalisilvae sp. Nov., representing a new genus of widespread freshwater bacteria. *International Journal of Systematic and Evolutionary Microbiology*, *69*(9), 2739–2749. https://doi.org/10.1099/ijsem.0.003554](https://www.zotero.org/google-docs/?BrCTHq)

[Pitt, A., Schmidt, J., Lang, E., Whitman, W. B., Woyke, T., & Hahn, M. W. (2018). Polynucleobacter meluiroseus sp. Nov., a bacterium isolated from a lake located in the mountains of the Mediterranean island of Corsica. *International Journal of Systematic and Evolutionary Microbiology*, *68*(6), 1975–1985. https://doi.org/10.1099/ijsem.0.002777](https://www.zotero.org/google-docs/?BrCTHq)

[Rincón-Rosales, R., Rogel, M. A., Rincón-Molina, C. I., Guerrero, G., Manzano-Gómez, L. A., López-López, A., Rincón Molina, F. A., & Martínez-Romero, E. (2023). Complete genome sequence of Exiguobacterium profundum TSS-3 isolated from an extremely saline-alkaline spring located in Ixtapa, Chiapas-México. *Microbiology Resource Announcements*, *12*(9), e00171-23. https://doi.org/10.1128/MRA.00171-23](https://www.zotero.org/google-docs/?BrCTHq)

[Rodríguez, M., Reina, J. C., Béjar, V., & Llamas, I. (2019). Paenibacillus lutrae sp. Nov., A Chitinolytic Species Isolated from A River Otter in Castril Natural Park, Granada, Spain. *Microorganisms*, *7*(12), Article 12. https://doi.org/10.3390/microorganisms7120637](https://www.zotero.org/google-docs/?BrCTHq)

[Santos, J. D. N. dos, Klimek, D., Calusinska, M., Lobo-da-Cunha, A., Catita, J., Gonçalves, H., González, I., Reyes, F., & Lage, O. M. (2023). Streptomyces meridianus sp. Nov. Isolated from brackish water of the Tagus estuary in Alcochete, Portugal. *International Journal of Systematic and Evolutionary Microbiology*, *73*(7), 005987. https://doi.org/10.1099/ijsem.0.005987](https://www.zotero.org/google-docs/?BrCTHq)

[Sheu, S.-Y., Huang, H.-W., Young, C.-C., & Chen, W.-M. (2015). Rhizobium alvei sp. Nov., isolated from a freshwater river. *International Journal of Systematic and Evolutionary Microbiology*, *65*(Pt_2), 472–478. https://doi.org/10.1099/ijs.0.065706-0](https://www.zotero.org/google-docs/?BrCTHq)

[Sheu, S.-Y., Shiau, Y.-W., & Chen, W.-M. (2013). Sphingobiumsufflavum sp. Nov., isolated from a freshwater lake. *International Journal of Systematic and Evolutionary Microbiology*, *63*(Pt_9), 3444–3450. https://doi.org/10.1099/ijs.0.051219-0](https://www.zotero.org/google-docs/?BrCTHq)

[Szuróczki, S., Abbaszade, G., Bóka, K., Schumann, P., Neumann-Schaal, M., & Tóth, E. (2022). Szabonella alba gen. Nov., sp. Nov., a motile alkaliphilic bacterium of the family Rhodobacteraceae isolated from a soda lake. *International Journal of Systematic and Evolutionary Microbiology*, *72*(1), 005219. https://doi.org/10.1099/ijsem.0.005219](https://www.zotero.org/google-docs/?BrCTHq)

[Thomas, F. A., Sinha, R. K., Hatha, A. A. M., & Krishnan, K. P. (2022). Phenylobacterium glaciei sp. Nov., isolated from Vestrebroggerbreen, a valley glacier in Svalbard, Arctic. *International Journal of Systematic and Evolutionary Microbiology*, *72*(5), 005375. https://doi.org/10.1099/ijsem.0.005375](https://www.zotero.org/google-docs/?BrCTHq)

[Wu, Y.-F., Wu, Q.-L., & Liu, S.-J. (2013). Paenibacillustaihuensis sp. Nov., isolated from an eutrophic lake. *International Journal of Systematic and Evolutionary Microbiology*, *63*(Pt_10), 3652–3658. https://doi.org/10.1099/ijs.0.050419-0](https://www.zotero.org/google-docs/?BrCTHq)

[Xamxidin, M., Huang, X., Yang, X., Wang, T., Chen, C., & Wu, M. (2022). Limnobacter parvus sp. Nov., a Thiosulfate-Oxidizing Bacterium Isolated from Lake Water. *Current Microbiology*, *80*(1), 39. https://doi.org/10.1007/s00284-022-03128-5](https://www.zotero.org/google-docs/?BrCTHq)

[Xu, D. Y., Leung, K. M., Lai, G. K. K., Leung, F. C. C., & Griffin, S. D. J. (2023). Complete Genome Sequence of Enterobacter roggenkampii RX.G5M56, a C1-Metabolizing Strain from a Freshwater Stream in Hong Kong. *Microbiology Resource Announcements*, *12*(4), e00015-23. https://doi.org/10.1128/mra.00015-23](https://www.zotero.org/google-docs/?BrCTHq)

[Yadav, S., & Dubey, S. K. (2018). Cellulose degradation potential of *Paenibacillus lautus* strain BHU3 and its whole genome sequence. *Bioresource Technology*, *262*, 124–131. https://doi.org/10.1016/j.biortech.2018.04.067](https://www.zotero.org/google-docs/?BrCTHq)

[Yan, L., Shi, J., Cao, J., Zhang, M., Chen, L., Wang, L., & Bao, Z. (2023). Paenibacillus sedimenti sp. Nov., isolated from freshwater wetland sediment. *International Journal of Systematic and Evolutionary Microbiology*, *73*(5), 005860. https://doi.org/10.1099/ijsem.0.005860](https://www.zotero.org/google-docs/?BrCTHq)

[Yin, L.-Z., Li, J.-L., Liu, Z.-T., Fang, B.-Z., Wang, P., Luo, X.-Q., Dong, L., Duan, L., Li, S.-H., & Li, W.-J. (2022). Deinococcus aestuarii sp. Nov. And Deinococcus aquaedulcis sp. Nov., two novel resistant bacteria isolated from pearl river estuary. *Antonie van Leeuwenhoek*, *115*(1), 59–68. https://doi.org/10.1007/s10482-021-01680-x](https://www.zotero.org/google-docs/?BrCTHq)

[Zothanpuia, Passari, A. K., Deka, P., Rajput, V., Priya, L. P. M., Dharne, M., Dastager, S., Mathew, O. K., Hashem, A., Abd_ Allah, E. F., & Singh, B. P. (2019). Draft Genome Sequence of Freshwater-Derived Streptomyces sp. Strain BPSDS2, Isolated from Damte Stream, Northeast India. *Microbiology Resource Announcements*, *8*(43), 10.1128/mra.00874-19. https://doi.org/10.1128/mra.00874-19](https://www.zotero.org/google-docs/?BrCTHq)
